# Alterations in the placental methylome with maternal obesity and evidence for metabolic regulation

**DOI:** 10.1371/journal.pone.0186115

**Published:** 2017-10-18

**Authors:** Kohzoh Mitsuya, Ashley N. Parker, Lu Liu, Jianhua Ruan, Margreet C. M. Vissers, Leslie Myatt

**Affiliations:** 1 Center for Pregnancy and Newborn Research, Department of Obstetrics and Gynecology, University of Texas Health Science Center at San Antonio, San Antonio, Texas, United States of America; 2 Department of Computer Science, University of Texas at San Antonio, San Antonio, Texas, United States of America; 3 Centre for Free Radical Research, Department of Pathology, University of Otago, Christchurch, New Zealand; Inc, UNITED STATES

## Abstract

The inflammatory and metabolic derangements of obesity in pregnant women generate an adverse intrauterine environment, increase pregnancy complications and adverse fetal outcomes and program the fetus for obesity and metabolic syndrome in later life. We hypothesized that epigenetic modifications in placenta including altered DNA methylation/hydroxymethylation may mediate these effects. Term placental villous tissue was collected following cesarean section from lean (prepregnancy BMI<25) or obese (BMI>30) women. Genomic DNA was isolated, methylated and hydroxymethylated DNA immunoprecipitated and hybridized to the NimbleGen 2.1M human DNA methylation array. Intermediate metabolites in placental tissues were measured by HPLC-ESI-MS, ascorbate levels by reverse phase HPLC and gene expression by RT-PCR. Differentially methylated and hydroxymethylated regions occurred across the genome, with a 21% increase in methylated but a 31% decrease in hydroxymethylated regions in obese vs lean groups. Whereas increased methylation and decreased methylation was evident around transcription start sites of multiple genes in the *GH/CSH* and *PSG* gene clusters on chromosomes 17 and 19 in other areas there was no relationship. Increased methylation was associated with decreased expression only for some genes in these clusters. Biological pathway analysis revealed the 262 genes which showed reciprocal differential methylation/ hydroxymethylation were enriched for pregnancy, immune response and cell adhesion-linked processes. We found a negative relationship for maternal BMI but a positive relationship for ascorbate with α-ketoglutarate a metabolite that regulates ten eleven translocase (TET) which mediates DNA methylation. We provide evidence for the obese maternal metabolic milieu being linked to an altered DNA methylome that may affect placental gene expression in relation to adverse outcomes.

## Introduction

Obesity is a growing challenge to public health affecting both adults and children. Obesity in pregnant women increases the number and severity of pregnancy complications and adverse fetal outcomes but critically also programs the fetus for obesity and metabolic syndrome in later life [1, 2]. Pregnancies in obese mothers generate an adverse intrauterine environment via their inflammatory milieu and metabolic derangements [3, 4]. By virtue of its roles at the maternal-fetal interface, and as the ‘director’ of pregnancy secreting peptides and hormones that regulate maternal metabolism, the placenta directs fetal growth and differentiation and both transduces and mediates the effects of the intrauterine environment on the fetus [5]. Hence, the placenta is central to the communication of the maternal environment to the developing fetus. Alterations in placental function are seen in association with adverse pregnancy outcomes including intrauterine growth restriction, preeclampsia, diabetes and obesity. There is currently a great deal of interest in epigenetic modifications (DNA methylation, histone acetylation) [6] that may mediate these changes in placental function. Indeed differences in placental DNA methylation have been described with preeclampsia [7–9] growth restriction [10] and gestational diabetes [11, 12].

The pathophysiology of the development of obesity is multifactorial and is not confined to a single phenotype. The rapid increase in the prevalence and transgenerational cycle of obesity suggests interactions of genes and environment factors involving epigenetic modifications of the genome including DNA methylation. In a recent mouse study expression of epigenetic machinery genes in placenta has been shown to be sensitive to obesity [13] suggesting obesity may lead to epigenetic changes. Past studies on placental epigenetics have focused on DNA methylation (5-methylcytosine, 5mC) at gene promoters which is usually is associated with gene repression. DNA hydroxymethylation (5-hydroxymethylcytosine, 5hmC) is a newly discovered epigenetic modification and appears to be linked to high levels of gene transcription [14]. While 5mC has been studied extensively, relatively little is known about the distribution and function of 5hmC. Following the discovery of ten eleven translocation (TET) proteins [15] that convert 5-methylcytosine (5mC) to 5-hydroxymethylcytosine (5hmC), it has recently been found that this TET-mediated active DNA demethylation requires α-ketoglutarate (αKG) and ascorbate (also known as vitamin C) as co-factors [16–18]. Since αKG is produced from isocitrate by isocitrate dehydrogenase (IDH) enzymes in the tricarboxylic acid (TCA) cycle, a potential link between intracellular metabolism and epigenetic modifications is suggested [19, 20]. We hypothesized that there would be differences in DNA methylation, both 5mC and 5hmC, in the placenta of pregnancies from obese women compared to healthy weight women. We have generated the genome-scale high-resolution maps of 5mC and 5hmC in human placentas of obese and healthy weight women, and have also investigated metabolic pathways that regulate the active DNA demethylation pathway.

## Materials and methods

### Collection of placental tissue

All tissue used in this study was collected in the period 2011–2013 with written informed consent under a protocol approved by the Institutional Review Board of The University of Texas Health Science Center San Antonio. Placental villous tissue was collected after delivery by elective cesarean section at term in the absence of labor from women with a range of adiposity (pre-pregnancy or first trimester BMI with a range of 18.5–45.0). All women were normotensive with no other medical complications of pregnancy and neonatal outcomes were normal. The placenta was placed basal plate downwards, obscuring the view of the villous tissue to remove collection bias, and 5 full thickness samples (2.5 × 2.5 cm) were dissected with sharp sterile scissors from the chorionic plate downwards to the basal plate. All samples were taken randomly from an area in a circle around the placenta at least 2.5 cm from the periphery. Basal plate and chorionic plate were then dissected away and the individual samples of villous tissue were rinsed in saline to remove excess blood, flash frozen in liquid nitrogen and stored at -80°C.

### Isolation of genomic DNA

Genomic DNA was extracted from each tissue sample using DNeasy Tissue and Blood Kit (Qiagen) according to the manufacturer’s instructions, followed by ethanol precipitation. The quality and concentration of isolated genomic DNA was evaluated using NanoDrop 2000 (Thermo Fisher Scientific) and each DNA sample was routinely assessed by agarose gel electrophoresis with ethidium bromide staining to ascertain the absence of contaminating RNA and to determine the extent of degradation of genomic DNA preparations. Villous placental genomic DNA samples derived from 10 obese (pre-pregnancy or first trimester BMI with a mean and SD values of 34.0 ± 2.9) and 10 healthy BMI (23.4 ± 2.3) women with uncomplicated pregnancy were combined, resulting in two pools of DNA (each 5 male and 5 female placentas) that were subjected to methylated/hydroxymethylated DNA immunoprecipitation (MeDIP/hMeDIP) assays.

### MeDIP and hMeDIP assays

MeDIP and hMeDIP assays were conducted according to the Roche NimbleGen methodology as previously described [21–23]. Essentially, fragmented genomic DNA (10 μg) was denatured and immunoprecipitated using the mouse monoclonal antibody against 5-methylcytosine (clone 33D3, Eurogentec) or rabbit polyclonal antibody against 5-hydroxymethylcytosine (Diagenode) respectively. A portion of the fragmented DNA (20%) was left untreated to serve as input control. Immunoprecipitated and input DNA fragments were amplified performed using GenomePlex Complete Whole Genome Amplification (WGA2) Kit (Sigma-Aldrich). After cleanup with QIAquick PCR Purification columns (Qiagen), immunoprecipitated and input DNA samples (1 μg) were denatured and differentially labeled with fluorescent Cy5 and Cy3 dyes, respectively, using Dual-Color DNA Labeling Kit (Roche NimbleGen). Sample concentrations were quantified by OD260 and equal amounts (34 μg) of Cy5- and Cy3-labeled DNA samples were combined and dried down using Savant DNA SpeedVac (Thermo Fisher Scientific). Two-color array hybridization was performed following the manufacturer’s instructions for the 2.1M Human DNA Methylation v2 genome tiling array (Roche NimbleGen). This array contains 2.1 million probes and has approximately 26,210 promoter regions from -8 to +3 kb of transcriptional start sites (TSS) and 27,867 CpG islands and 730 microRNA promoters (15 kb upstream to mature microRNAs) tiled on it. The array also contained positive and negative controls and non-CpG array controls in triplicate.

### Tiling array data analysis

Raw DNA methylation array data was processed using NimbleScan (Roche NimbleGen) to obtain the statistical significance (*P*-value) of the differential methylation between the subjects (lean or obese) and their controls for each probe on the array. Probes that overlap with a certain gene feature (e.g., TSS1500 regions defined as between -1,500 bp and +500 bp to TSS) were assigned to the nearest gene using BEDTools [24]. The *P*-values for all probes associated with a particular gene in the lean and obese samples were used to derive the statistical significance (*P*-value) of the differential methylation between lean and obese using the Fisher’s combined probability test [25]. Finally, genes with a *P*-value ≤ 0.05 in the Fisher’s combined probability test were deemed differentially methylated between lean and obese. These genes were further divided into two groups, representing genes that are either hypermethylated in obese or hypomethylated in obese. Functional enrichment of known pathways and Gene Ontology (GO) terms were determined using the Database for Annotation, Visualization and Integrated Discovery (DAVID) web tool [26].

### Mass spectrometry of metabolites

Frozen placental tissue (n = 84 subjects, early pregnancy BMI 18.5–45.0) was homogenized to a fine powder in liquid nitrogen and homogenates (~50 mg) were extracted with ice-cold 80% aqueous methanol containing isotope labeled standard [1,2,3,4-13C4]α-ketoglutaric acid (Cambridge Isotope Laboratories) and maintained at -20°C for 1 h. Subsequently, the extracts were centrifuged at 13,800 × g for 10 minutes and the supernatants were transferred to glass autosampler vials for HPLC electrospray ionization-mass spectrometry (HPLC-ESI-MS) analysis on a Thermo Fisher Q Exactive mass spectrometer with on-line separation by a Thermo Fisher/Dionex Ultimate 3000 HPLC. HPLC conditions were: column, Synergi Polar-RP, 4 μm, 2 × 150 mm (Phenomenex); mobile phase A, 0.1% formic acid in water; mobile phase B, 0.1% formic acid in acetonitrile; flow rate, 250 μl/min; gradient, 1% B to 5% B over 5 minutes, 5% B to 95% B over 1 minutes and held at 95% B for 2 minutes. Metabolite identification was based on the metabolite accurate mass (± 5 ppm) and agreement with the HPLC retention time of authentic standards. Quantification was made by integration of extracted ion chromatograms of αKG followed by comparison with the corresponding standard curves.

### Ascorbate measurement

Frozen placental tissue (n = 63 subjects, early BMI 18.5–45.0) was ground to a fine powder in liquid nitrogen. The ground powder was weighed, suspended in 5 mM phosphate buffer, pH 7.4 and the ascorbate fraction extracted with 0.54 M perchloric acid containing diethylenetriaminepentaacetic acid (DTPA) as described previously (Kuiper et al., 2010). The protein precipitate was removed by centrifugation and ascorbate was analyzed in the supernatant by reverse-phase HPLC with electro-chemical detection (mobile phase: 80 mM sodium acetate, pH 4.8, with 0.54 mM DTPA) as previously described using a fresh standard curve of sodium-l-ascorbate for each run.

### Real-time RT-PCR

Total RNA was isolated from tissue (n = 63 subjects, early BMI 18.5–45.0) using RNeasy Mini Kit (Qiagen) coupled with on-column DNA digestion following the manufacturer’s standard protocol. RNA concentrations were determined using NanoDrop 2000. First strand cDNA was generated from 0.5 μg total RNA per sample using QuantiTect Reverse Transcriptase (Qiagen) with a mixture of random and oligo(dT) primers following the supplied protocol. cDNA samples were diluted 1:10 with DNase/RNase-free water, aliquoted to prevent freeze thawing and stored at -80°C prior to use. Real-time PCR was performed on StepOne Plus (Life Technologies) using SYBR Green PCR Master Mix (Life Technologies) according to the manufacturer's recommended protocol. Quantification was performed and target gene expression normalized to 18S rRNA, the expression of which did not differ between the groups. A list of all predesigned PCR primers (Integrated DNA Technologies) used in the qRT-PCR assay is provided in S5 Table.

### Pyrosequencing

Prior to pyrosequencing reactions, TET-assisted bisulphite (TAB) treatment was carried out as essentially described previously [27, 28]. Briefly, purified genomic DNA was glucoslyated and oxidized using 5hmC TAB-Seq Kit (WiseGene) as described by the manufacturer, except for the WiseGene T4 phage β-glucosyltransferase (T4-BGT) was replaced with the equivalent product from New England Biolabs. After bisulfite conversion using EpiTect Fast Bisulfite Kit (Qiagen), pyrosequencing was performed using PyroMark Q96 MD with PyroMark CpG assays (PM00061215 and PM00026768) as essentially described previously [29].

## Results and discussion

### Alterations in the placental epigenome with maternal adiposity

**Fig 1:**
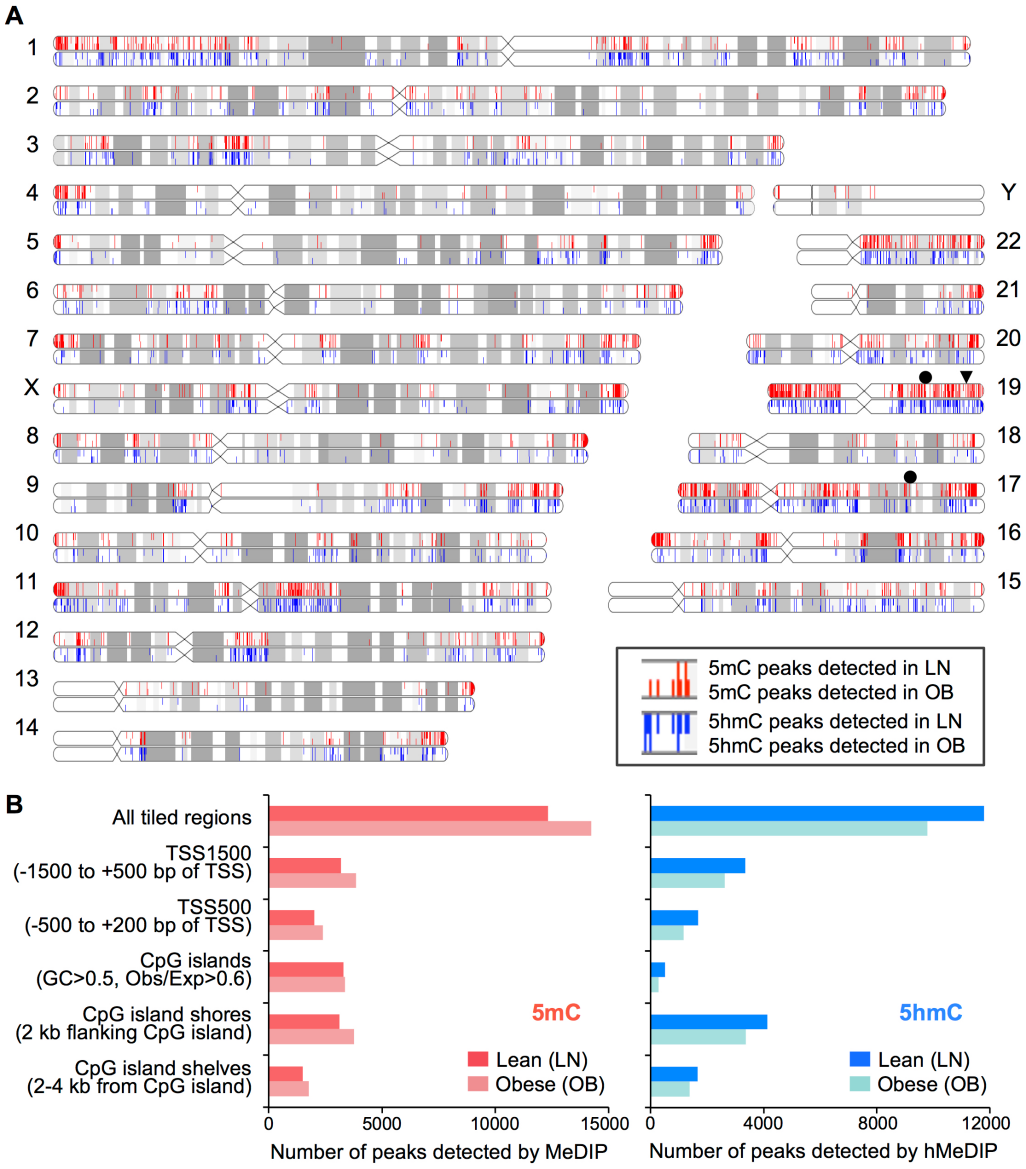
Widespread alterations to the placental epigenome identified throughout the genome in the setting of maternal obesity. (A) Chromosome ideograms showing the genome-wide distributions of methylated and hydroxymethylated peak regions. Differences in 5mC and 5hmC distributions between placentas of obese vs. lean pregnancies (n = 10 placentas combined each group) were found widespread across the genome. Red and blue vertical lines indicate locations of methylated and hydroxymethylated genomic regions, respectively. Adjacent upper and lower lines represent peak regions detected in lean and obese pregnancies, respectively. The locations of the *GH-CSH* gene cluster on chromosome 17q24 and *PSG* gene cluster on chromosome 19q13 are indicated by closed circles. The pregnancy-associated miRNA cluster is also located on chromosome 19q13 (closed triangle). (B) Number of methylated (5mC) and hydroxymethylated (5hmC) peak regions identified by MeDIP and hMeDIP assays. Increased numbers of methylated peaks (right panel) and decreased numbers of hydroxymethylated peaks (left panel) were consistently detected at various parts of the genome including CpG islands, CpG island shores and shelves.

**Fig 2:**
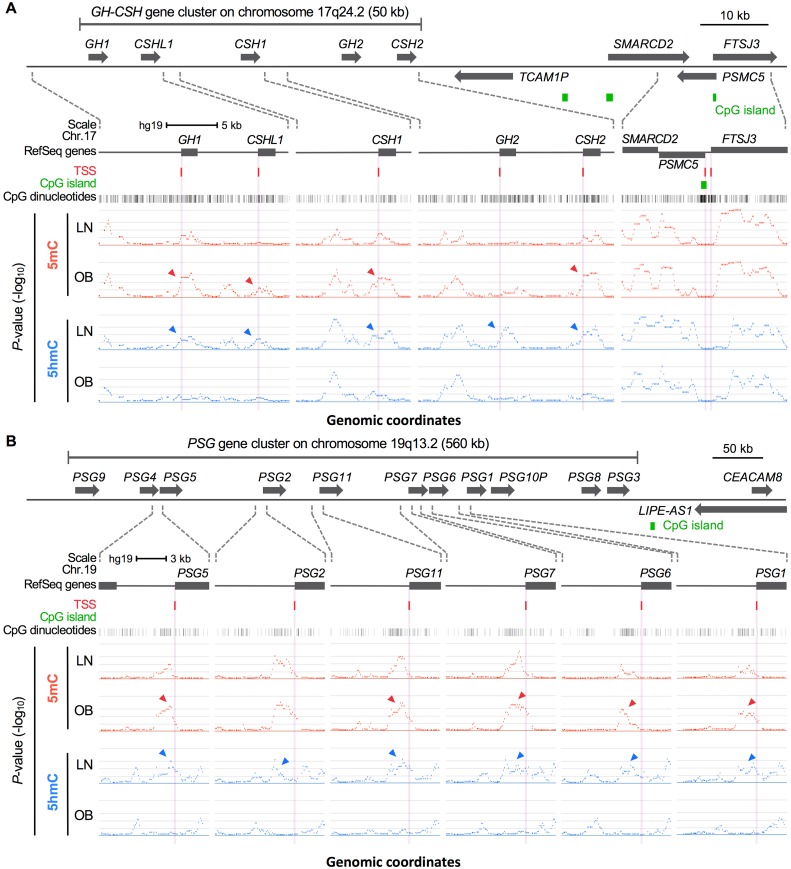
Reciprocal increase in 5mC and decrease in 5hmC at the pregnancy-associated gene clusters in placentas of obese compared to lean mothers. (A) Reciprocal increase in 5mC and decrease in 5hmC at the *GH-CSH* gene cluster on human chromosome 17q24. Differential DNA methylation (5mC, red) and hydroxymethylation (5hmC, blue) was evident at the human growth hormone-chorionic somatomammotropin (hGH/hCS/hPL) gene cluster between lean (LN) and obese (OB) epigenomes and this contrasted with the sharp resemblance in 5mC and 5hmC profiles outside the *GH-CSH* gene cluster as indicated in the rightmost panel. Marked differences in 5mC and 5hmC distributions are indicated by arrowheads. (B) Reciprocal increase in 5mC and decease in 5hmC at the *PSG* gene cluster on human chromosome 19q13. Genomic features are viewed as custom tracks in the UCSC genome browser. TSS, transcriptional start sites.

To evaluate the effect of maternal adiposity on the placental epigenome, we determined the genome-scale distribution of 5mC and 5hmC by antibody-based MeDIP and hMeDIP assays comparing placentas of obese vs. healthy weight (lean) women, each with uncomplicated pregnancies. The specificity of the methylation array for 5mC was shown using the positive and negative controls (S1A Fig and S1 Table). No hydroxymethylation was shown in these controls or within the 4 imprinting control regions in placenta. Chromosome plots of the methylated and hydroxymethylated peaks indicated that differences in 5mC and 5hmC between obese and lean groups were widely distributed throughout the genome (Fig 1A). Analysis of the data in the entire tiling array showed increases of up to 21% in the numbers of methylated regions and decreases of up to 31% in the numbers of hydroxymethylated regions across the discrete regions of the genome, including around transcription start sites, CpG islands, island shores and island shelves, in obese compared to lean groups (Fig 1B and S1B Fig). We then examined differentially methylated and hydroxymethylated regions located within a 100-bp window on either side of transcription start sites (TSS). Based on the ‘bump hunting’ strategy [30], we identified eight genes and two gene clusters with increased methylation, one gene with decreased methylation, and three genes with decreased hydroxymethylation in this 100-bp window comparing obese vs. lean groups (S2 Table), which were subjected to further expression analysis in a separate group of placentas from 21 early pregnancy obese and 21 early pregnancy lean women (S3 Table and see below). Genes with increased methylation located within the 100-bp window spanning TSS included the pregnancy-specific glycoprotein (*PSG*) gene cluster on chromosome 19q13 and growth hormone-chorionic somatommamotropin hormone (*GH-CSH*) gene cluster on chromosome 17q24. The latter encodes hormones, which are the most abundant fetal proteins in maternal circulation, are synthesized by placental syncytiotrophoblasts, and have vital roles in the control of maternal metabolism, fetal growth and placental development in pregnancy. As exemplified in Fig 2, increased methylation (5mC) and reciprocally decreased hydroxymethylation (5hmC) in placental tissue of obese vs. lean pregnancies was evident around TSS of the multiple genes on these two pregnancy-associated gene clusters including *GH1*, *CSHL1*, *CSH1*, *CSH2*, *PSG5*, *PSG11*, *PSG7*, *PSG6*, and *PSG1* genes. Note however, that there are areas of increased methylation that do not correspond to decreased hydroxymethylation and vice versa.

Genes with decreased methylation within the 100-bp window in the setting of obesity included *CMTM1* (S2 Table). Bisulfite treatment alone does not distinguish 5mC from 5hmC, therefore to confirm the validity of tiling array data, we used TET-assisted bisulfite (TAB)-pyrosequencing to evaluate the degree of DNA methylation and hydroxymethylation at the 5' CpG island of the *CMTM1* gene. Using placentas of 21 early pregnancy obese and 21 early pregnancy lean women (S3 Table), decreased 5mC accompanied by increased 5hmC was found in a 189-bp region spanning 4 CpG dinucleotides within the *CMTM1* CpG island, which was in agreement with the tiling array data (S2 Fig).

### Potential functional significance of reciprocal increase in 5mC and decrease in 5hmC associated with maternal adiposity

**Fig 3:**
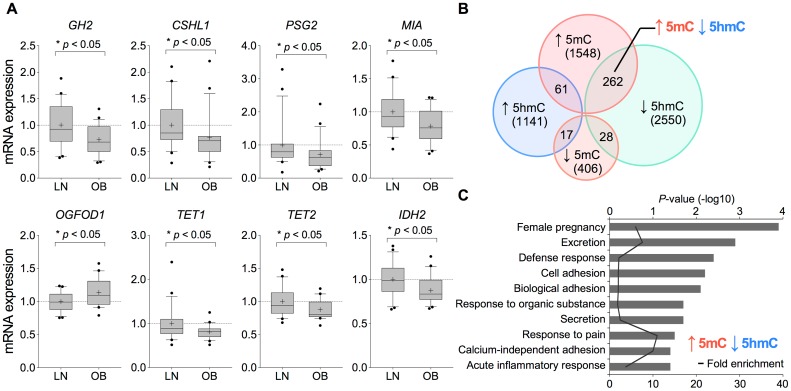
Relationship of differential distribution in 5mC and 5hmC between the obese and lean placental epigenomes to gene expression. (A) qRT-PCR analysis of genes found to be differentially methylated and/or hydroxymethylated between placentas of obese (n = 21) and lean (n = 21) mothers (S3 Table). In each box-and-whisker plot, the lower and upper edges of the box indicate the 10th and 90th percentiles; the horizontal line within the box represents median; the plus sign indicates mean; outliers from the range of 10–90 percentile are shown as closed circles. Statistical analyses were performed using Mann-Whitney *U*-test (*P < 0.05). (B) Venn diagram showing overlaps of differentially methylated and hydroxymethylated loci. (C) Functional enrichment analysis of biological process for the significant overlap between genes with increased 5mC and reciprocally decreased 5hmC. The graph shows GO biological process terms associated with 262 common genes exhibited reciprocal increase in 5mC and decrease in 5hmC in placentas of the obese compared to lean mothers.

To relate differences we observed in 5mC and 5hmC distributions with gene transcription, we used real-time reverse transcription PCR (RT-PCR) to quantify mRNA expression of the genes that were differentially methylated or hydroxymethylated in the 100-bp window between obese and lean groups (S2 Table). This was carried out in full-term placental tissue of the 21 obese and 21 lean women that was utilized for TAB-pyrosequencing (S3 Table). As shown in Fig 3A, increased 5mC seen with maternal obesity was associated with decreased mRNA expression of three genes in the *GH-CSH* gene cluster (*GH2* and *CSHL1*) and *PSG* gene cluster (*PSG2*) as well as the *MIA* gene that is also located on human chromosome 19q13. However expression of CSH and PSG proteins were not measured in placental tissue. In contrast, increased 5hmC seen with maternal obesity was associated with increased mRNA expression of the *OGFOD1* (2-oxoglutarate and iron-dependent oxygenase domain containing 1) gene.

As mentioned earlier, analysis of the data in the entire tiling array indicated increased 5mC accompanied by decreased 5hmC with maternal obesity, suggesting a potential decrease in conversion efficiency of 5mC to 5hmC by TET proteins. In this context, TET dioxygenases require αKG (also referred as 2-oxoglutarate), which is generated from isocitrate in the TCA cycle by IDH enzymes, as an essential co-factor [19]. Mutually exclusive defects in the TET-mediated DNA demethylation pathway in cancer cells leads to increased levels of 5mC and concomitant depletion of 5hmC [31, 32]. In addition, mutated IDH enzymes produce a competitive inhibitor of αKG, 2-hydroxyglutarate (2HG), the addition of which can increase 5mC and also decrease 5hmC [33–35]. We therefore included three TET (*TET1*, *TET2* and *TET3*) and five IDH family genes (*IDH1*, *IDH2*, *IDH3A*, *IDH3B*, *IDH3G*) into expression analysis. As shown in Fig 3A, placental mRNA levels of *TET1*, *TET2* and *IDH2* were decreased with increased maternal obesity. Changes in mRNA levels of the remaining genes were not evident (S3A Fig). We did not measure protein expression for any genes.

We subsequently extended the analysis to MeDIP and hMeDIP data sets using a 2.0-kb window from -1.5 to +0.5 kb from TSS and identified a total of 5,645 differentially methylated or hydroxymethylated genes across the entire genome (1548 loci with increased 5mC, 406 loci with decreased 5mC, 1141 loci with increased 5hmC, and 2550 loci with decreased 5hmC in obese vs. lean pregnancies, Fig 3B). Interestingly we observed increased methylation but no difference in hydroxymethylation of TET3 within this window nor any changes in methylation or hydroxymethylation of TET1, TET2 and IDH2. GO biological pathway analysis indicated that differentially methylated genes in placentas of obese compared to lean groups were enriched for metabolism-related excretion, secretion, pregnancy, placental development, decidualization and embryonic development ending in birth (S3B Fig and S4 Table). In parallel, genes with differential hydroxymethylation were involved in pregnancy and hormone secretion as well as in lipid/cholesterol storage, glucose transport, defense/inflammatory response, response to wounding and detection of stimulus (S3C Fig and S4 Table). Interestingly, genes acquiring 5mC with increased maternal adiposity showed partial but significant overlap (262 common genes) with genes that exhibited a decrease in 5hmC. In addition to pathways related to pregnancy and immune response, cell adhesion-linked biological processes were among the most enriched for the 262 common genes showing increase in 5mC and reciprocal decrease in 5hmC (Fig 3C and S4 Table). These observations suggest that obesity-associated alterations to the placental epigenome may influence a wide range of feto-placental functions throughout human pregnancy. The findings also extend previous observations that obesity is associated with subclinical chronic inflammation and point to novel molecular targets for further characterization of obesity and associated comorbidities such as type 2 diabetes (T2DM).

### Correlations between placental metabolites and gene expression and maternal BMI

**Fig 4:**
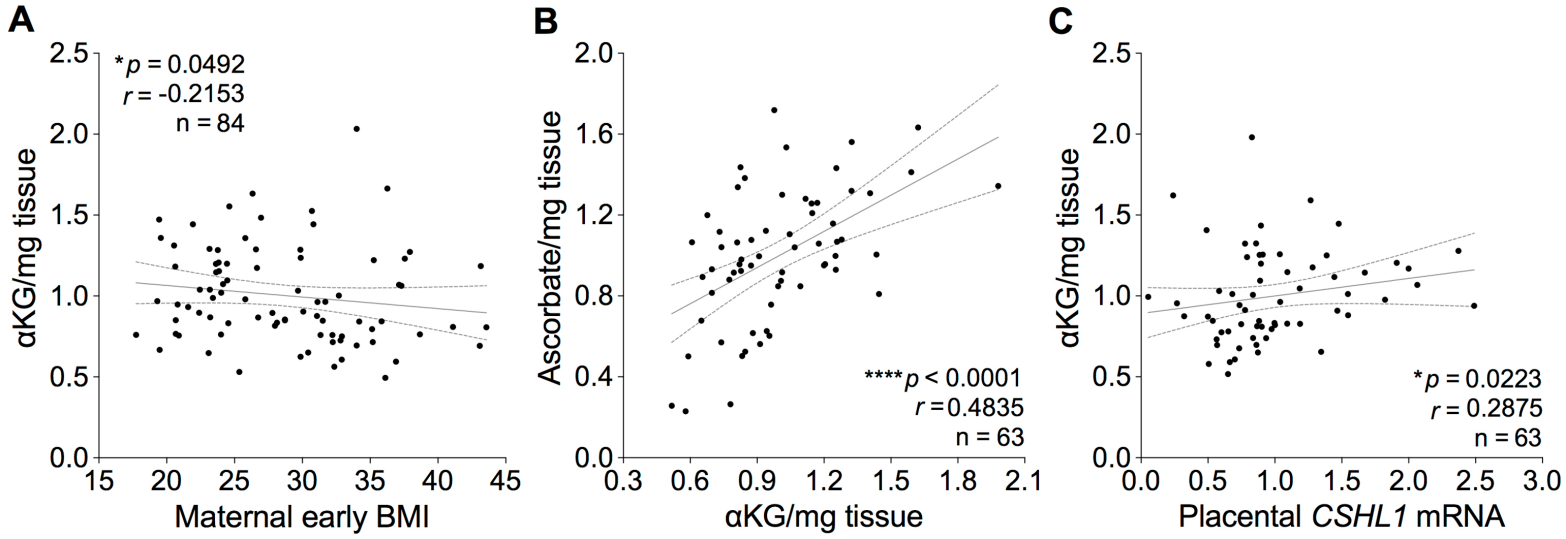
Relationship between maternal adiposity, intermediary metabolites and placenta-specific gene expression. (A-C) Correlations between placental levels of αKG and maternal BMI (A) or ascorbate (B), and between αKG and *CSHL1* mRNA levels (C). The relations between continuous variables were evaluated by Spearman correlations (*P < 0.05). Pre-pregnancy or first trimester BMI was used as maternal early BMI.

Since the genome-scale profiling of 5mC and 5hmC suggests that maternal adiposity modulates the placental epigenome during pregnancy, in which involvement of an oxidative DNA demethylation process is implicated, we asked whether changes in metabolite composition were associated with the obesity-linked remodeling of the placental epigenome. As shown in Fig 4A, a weak but significant negative correlation was found between maternal early BMI and αKG levels in placentas of women. No associations with maternal BMI were observed for any of the other metabolites examined in this study (S4 Fig). No alteration in 2HG levels was noted despite the slight reduction in IDH2 mRNA expression suggesting that this is not responsible for the decrease in 5hmC. It has recently reported that ascorbate (vitamin C) facilitates the TET-mediated DNA demethylation process [16–18]. We therefore determined ascorbate levels and found no significant correlation with maternal BMI (data not shown) but a close positive association of ascorbate with αKG levels (*P* < 0.0001, Fig 4B). Notably, these findings are consistent with previous clinical and experimental studies. Comprehensive metabolomic profiling has revealed that αKG levels are considerably decreased in the obese population [36] and increased in response to weight loss intervention [37]. It has also been well documented that a substantial decrease in ascorbate levels is closely associated with obesity as well as metabolic syndrome, and multiple lines of evidence indicate that both ascorbate and αKG levels are decreased in T2DM patients and animal models of obesity and T2DM [38–41]. Increasing maternal BMI was also associated with decreased ratios of αKG to glucose (*P = 0.0100), pyruvate (*P = 0.0250), glutamate (*P = 0.0241) and glutamine (*P = 0.0401) but not to succinate (*P* = 0.8383, S5 Fig), which have been implicated in the regulation of αKG-dependent DNA demethylation by TET dioxygenases [42].

Finally we asked whether there was any correlation with gene expression in placentas of women over a range of adiposity. A small but positive association was observed between levels of *CSHL1* mRNA and αKG (Fig 4C), and more substantial correlations between levels of *CSHL1*, *TET2* and *IDH2* mRNA (S6A Fig). In addition, levels of placental *IDH2* mRNA were negatively correlated with maternal BMI (S6B Fig). The CSHL1 (chorionic somatomammotropin hormone-like 1) protein is a member of the somatotropin/prolactin family of hormones and is located on the *GH-CSH* gene cluster on chromosome 17q24. *GH-CSH* gene cluster encodes human chorionic somatomammotropins (hCS or human placental lactogen, hPL) and growth hormones (hGH) that regulate maternal metabolism by stimulation of lipolysis to supply fatty acids as maternal fuel and cause maternal insulin resistance promoting glucose availability for the fetus. Although there is little data on the effect of maternal adiposity on gene expression of the hCS/hGH cluster or on hormone levels in pregnancy, our data suggest that the gene cluster can be epigenetically regulated and that these both may be related to the maternal metabolic milieu, e.g., obesity but, via the actions of hCS and hGH on maternal metabolism, may be part of a feedback loop controlling maternal and placental metabolism and ultimately fetal growth.

## Conclusions

In summary, we provide a high-resolution map of the human epigenome associated with obesity and evidence for environmental regulation of the placental epigenome that may relate to the adverse pregnancy outcomes. Remodeling of the placental epigenome with maternal adiposity presented in this study may also define a nutrient-sensing network that links intracellular metabolic pathways and epigenetic regulation, and highlights the idea that the TET-mediated active DNA demethylation pathway can sense metabolic derangements and plays a role in subsequent cellular adaptation [19, 20]. This is supported by *in vitro* and *in vivo* studies. Supplementation of cell cultures with a cell-permeable form of αKG reduces global levels of 5mC [42]. In parallel, a rapid increase in αKG seen following administration of glucose is accompanied with a gain of 5hmC both at global and gene-specific levels in multiple mouse tissues and conversely, this concomitant increase in αKG and 5hmC is abrogated to basal levels upon glucose withdrawal [43]. Since αKG and ascorbate serve as co-substrates for a large family of dioxygenases including HIF hydroxylases, RNA and histone demethylases [44–47], it is of interest to investigate the role of αKG-dependent dioxygenases in the development of obesity and associated comorbidities.

## Supporting information

S1 Fig **A: Selective enrichment of methylated DNA fragments in the MeDIP preparations**. Top panel: whole genome amplified DNA spreads (100–1,000 bp) of placental villous tissue from obese and healthy weight (lean) pregnancies that were resolved on a 2.0% agarose gel and visualized using GelRed staining (top panel). The negative control (NTC) was run by addition of water in place of DNA templates, resulting in no detectable signals. S, DNA size marker; NTC, no template control. Bottom Panel: Specific enrichment on the positive locus (*TSH2B*) comparing no enrichment on the negative locus (*GAPDH*). The whole genome amplified products from immunoprecipitated (MeDIP) and non-immunoprecipitated (input) DNA fragments were subjected to PCR amplification with primer pairs specific to the 5' region of the *TSH2B* and *GAPDH* genes. The *TSH2B/HIST1H2BA* (*testis-specific histone 2B*) gene is expressed exclusively in testis but not in somatic tissues. The region amplified with the *TSH2B* primer pair corresponds to the genomic locus that is unmethylated in testis but is highly methylated in somatic cells was used as a control gene representing inactive methylated regions. The *GAPDH* primer pair that is designed from the constitutively active promoter region was used as an unmethylated negative control gene. Preferential amplification was detected for the methylated 5' region of the *TSH2B* gene in the MeDIP preparations and for the unmethylated 5' region of the *GAPDH* gene in the input DNA. A 100-bp ladder was used as a DNA size marker (lane S). No amplification was detected in the no template controls (NTC). **B:. Differences in the degrees of DNA methylation and hydroxymethylation detected in the placental epigenomes between lean and obese pregnancies**. Differences in number of peaks detected by MeDIP (top panel) and hMeDIP (bottom panel) were evident particularly at higher significance thresholds.
(TIF)

S2 Fig Verification of the MeDIP data by TAB-pyrosequencing. (A) Decreased 5mC at the *CMTM1* CpG island detected in obese (OB) compared to lean (LN) pregnancies by MeDIP assay. A marked decrease in 5mC within the 5' CpG island of the *CMTM1* gene is indicated by arrowhead. A 189-bp genomic region spanning four CpG dinucleotides analyzed by TAB-pyrosequencing is indicated by gray horizontal bar. (B) TAB-pyrosequencing analysis of the *CMTM1* CpG island using placentas of 21 obese and 21 lean women. No statistically significant differences were detected by conventional bisulphite sequencing. Vertical dashed lines represent mean. For group comparison, the Mann-Whitney U test was used (*P < 0.05).
(TIF)

S3 Fig **A: mRNA expression levels of differentially methylated and/or hydroxymethylated genes using full-term placentas of obese and lean mothers**. qRT-PCR analysis was carried out using placental villous tissue of 21 lean (LN) and 21 obese (OB) mothers (S3 Table). The statistical significance was assessed as indicated in the legend to Fig 4A. No statistically significant difference was detected in the mRNA levels of the *IDAX/CXXC4* gene which is transcribed in the opposite direction of the *TET2* gene and regulates its expression level [48]. The *DSCR4* gene is transcribed through a transposon-derived promoter that is unmethylated in human placenta but is highly methylated in maternal blood cells [49] and was used as an unmethylated control gene in this study (S1 and S2 Tables). **B and C: Functional enrichment analysis of biological process for differentially methylated (B) and hydroxymethylated (C) genes**.
(TIF)

S4 Fig Relationship between maternal adiposity and intermediary metabolites. Correlations between placental levels of A. Glucose, B. Lactate, C. Citrate + isocitrate, D. Succinate, E. Glutamate, F. Glutamine, G. Aconitate, H. 2-hydroxyglutarate, I. Fumarate 1, J. Fumarate 2, K. Malate, L. Oxaloacetate, M. Glucose-6-phosphate, N. Fructose-1,6-biphosphate, O. Glycerate-3-phosphate, P. Phosphoenolpyruvate, Q. D-Ribose 5-phosphate and maternal BMI. The relations between continuous variables were evaluated by Spearman correlations.
(TIF)

S5 Fig Relationship between maternal adiposity and intermediary metabolites implicated in the regulation of αKG-dependent DNA demethylation by TET dioxygenases. Correlations between placental levels of A. αKG/glucose, B. αKG/pyruvate, C. αKG/glutamate, D. αKG/glutamine, E. αKG/succinate and maternal BMI. The relations between continuous variables were evaluated by Spearman correlations.
(TIF)

S6 Fig A and B: Relationship between maternal adiposity, placental metabolites and mRNA expression. Correlations between *TET2*, *IDH2*, and *CSHL1* mRNA levels (A) and between placental *IDH2* levels and maternal BMI (B). Pre-pregnancy or first trimester BMI was used as maternal early BMI. The statistical significance was evaluated as indicated in the legend to Fig 4.
(TIF)

S1 Table DNA methylation and hydroxymethylation at control genes detected in placentas of obese and lean pregnancies by MeDIP and hMeDIP assays. (TIF)

S2 Table Genes that were subjected to mRNA expression analysis. (TIF)

S3 Table Demographic and clinical characteristics of pregnancies used in mRNA expression and pyrosequencing analyses. (TIF)

S4 Table Pathway enrichment analysis of placental genes in the setting of maternal adiposity. (PDF)

S5 Table List of primers used for qPCR assays. (TIF)
