# Supplementary figures and images for: Alterations in the placental methylome with maternal obesity and evidence for metabolic regulation
Source: PLoS One. 2017 Oct 18;12(10):e0186115. doi: 10.1371/journal.pone.0186115 (PMC5646778; doi:10.1371/journal.pone.0186115)

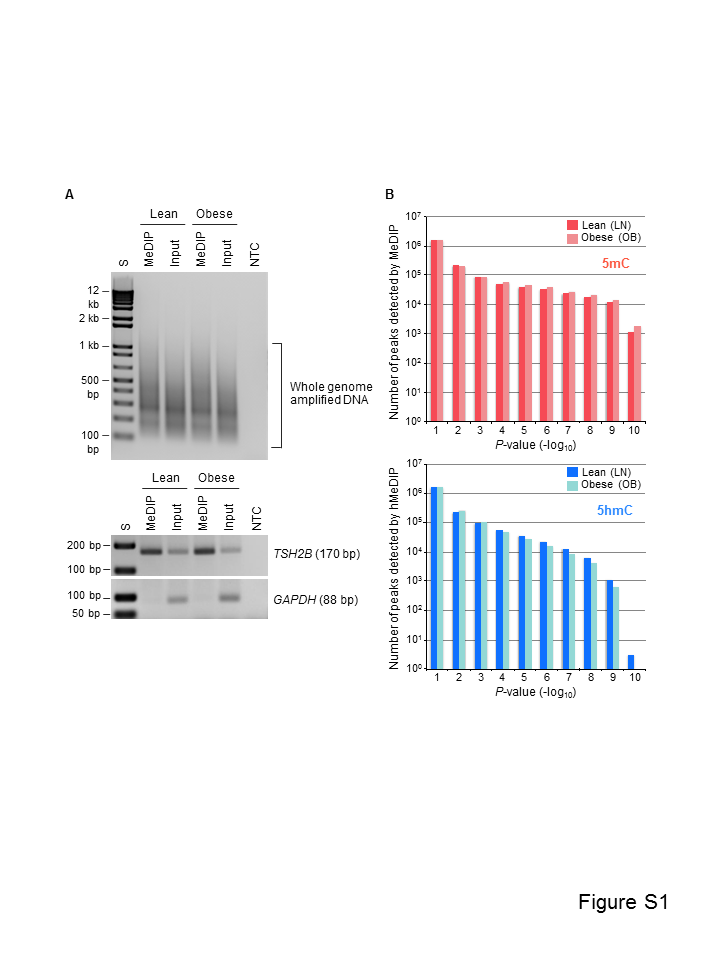

Supplement: S1 Fig — A: Selective enrichment of methylated DNA fragments in the MeDIP preparations. Top panel: whole genome amplified DNA spreads (100–1,000 bp) of placental villous tissue from obese and healthy weight (lean) pregnancies that were resolved on a 2.0% agarose gel and visualized using GelRed staining (top panel). The negative control (NTC) was run by addition of water in place of DNA templates, resulting in no detectable signals. S, DNA size marker; NTC, no template control. Bottom Panel: Specific enrichment on the positive locus (TSH2B) comparing no enrichment on the negative locus (GAPDH). The whole genome amplified products from immunoprecipitated (MeDIP) and non-immunoprecipitated (input) DNA fragments were subjected to PCR amplification with primer pairs specific to the 5' region of the TSH2B and GAPDH genes. The TSH2B/HIST1H2BA (testis-specific histone 2B) gene is expressed exclusively in testis but not in somatic tissues. The region amplified with the TSH2B primer pair corresponds to the genomic locus that is unmethylated in testis but is highly methylated in somatic cells was used as a control gene representing inactive methylated regions. The GAPDH primer pair that is designed from the constitutively active promoter region was used as an unmethylated negative control gene. Preferential amplification was detected for the methylated 5' region of the TSH2B gene in the MeDIP preparations and for the unmethylated 5' region of the GAPDH gene in the input DNA. A 100-bp ladder was used as a DNA size marker (lane S). No amplification was detected in the no template controls (NTC). B:. Differences in the degrees of DNA methylation and hydroxymethylation detected in the placental epigenomes between lean and obese pregnancies. Differences in number of peaks detected by MeDIP (top panel) and hMeDIP (bottom panel) were evident particularly at higher significance thresholds. (TIF) [file pone.0186115.s001.tif]

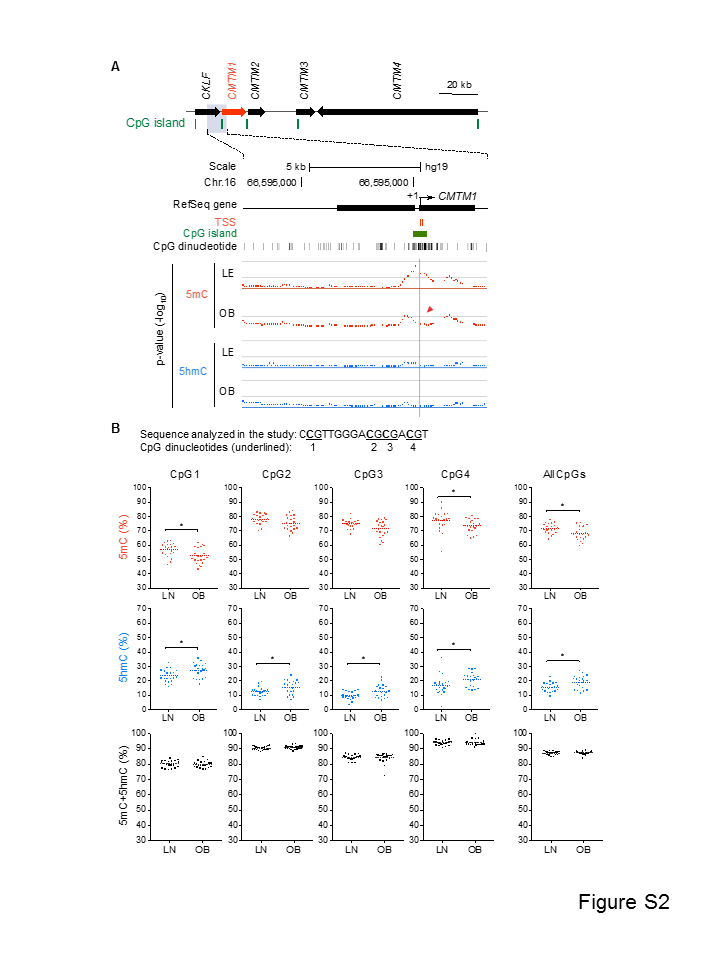

Supplement: S2 Fig — (A) Decreased 5mC at the CMTM1 CpG island detected in obese (OB) compared to lean (LN) pregnancies by MeDIP assay. A marked decrease in 5mC within the 5' CpG island of the CMTM1 gene is indicated by arrowhead. A 189-bp genomic region spanning four CpG dinucleotides analyzed by TAB-pyrosequencing is indicated by gray horizontal bar. (B) TAB-pyrosequencing analysis of the CMTM1 CpG island using placentas of 21 obese and 21 lean women. No statistically significant differences were detected by conventional bisulphite sequencing. Vertical dashed lines represent mean. For group comparison, the Mann-Whitney U test was used (*P < 0.05). (TIF) [file pone.0186115.s002.tif]

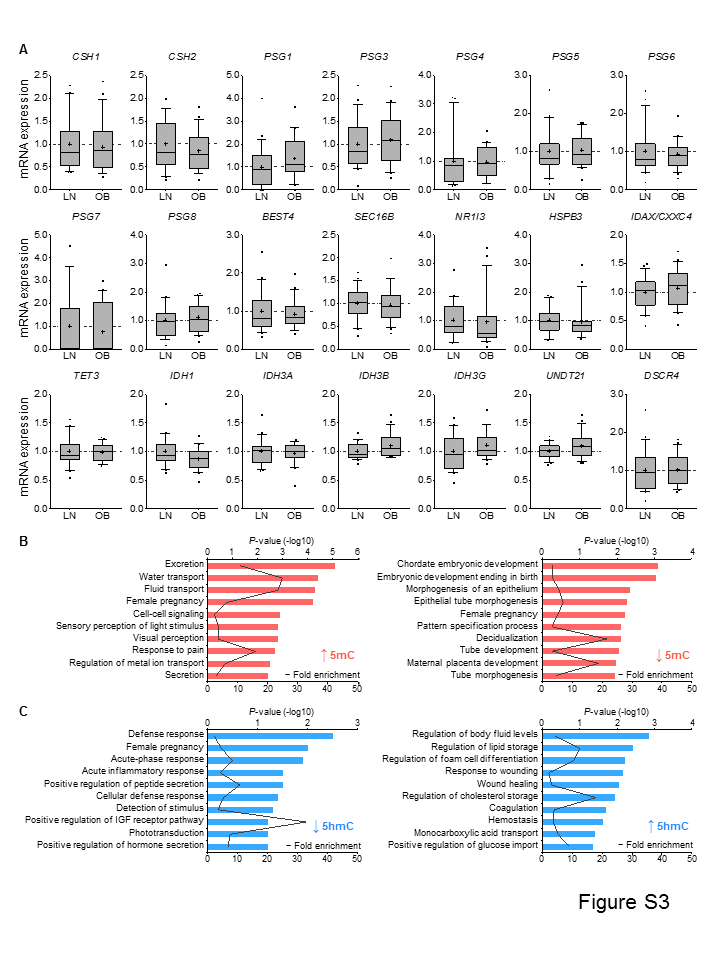

Supplement: S3 Fig — A: mRNA expression levels of differentially methylated and/or hydroxymethylated genes using full-term placentas of obese and lean mothers. qRT-PCR analysis was carried out using placental villous tissue of 21 lean (LN) and 21 obese (OB) mothers (S3 Table). The statistical significance was assessed as indicated in the legend to Fig 4A. No statistically significant difference was detected in the mRNA levels of the IDAX/CXXC4 gene which is transcribed in the opposite direction of the TET2 gene and regulates its expression level [48]. The DSCR4 gene is transcribed through a transposon-derived promoter that is unmethylated in human placenta but is highly methylated in maternal blood cells [49] and was used as an unmethylated control gene in this study (S1 and S2 Tables). B and C: Functional enrichment analysis of biological process for differentially methylated (B) and hydroxymethylated (C) genes. (TIF) [file pone.0186115.s003.tif]

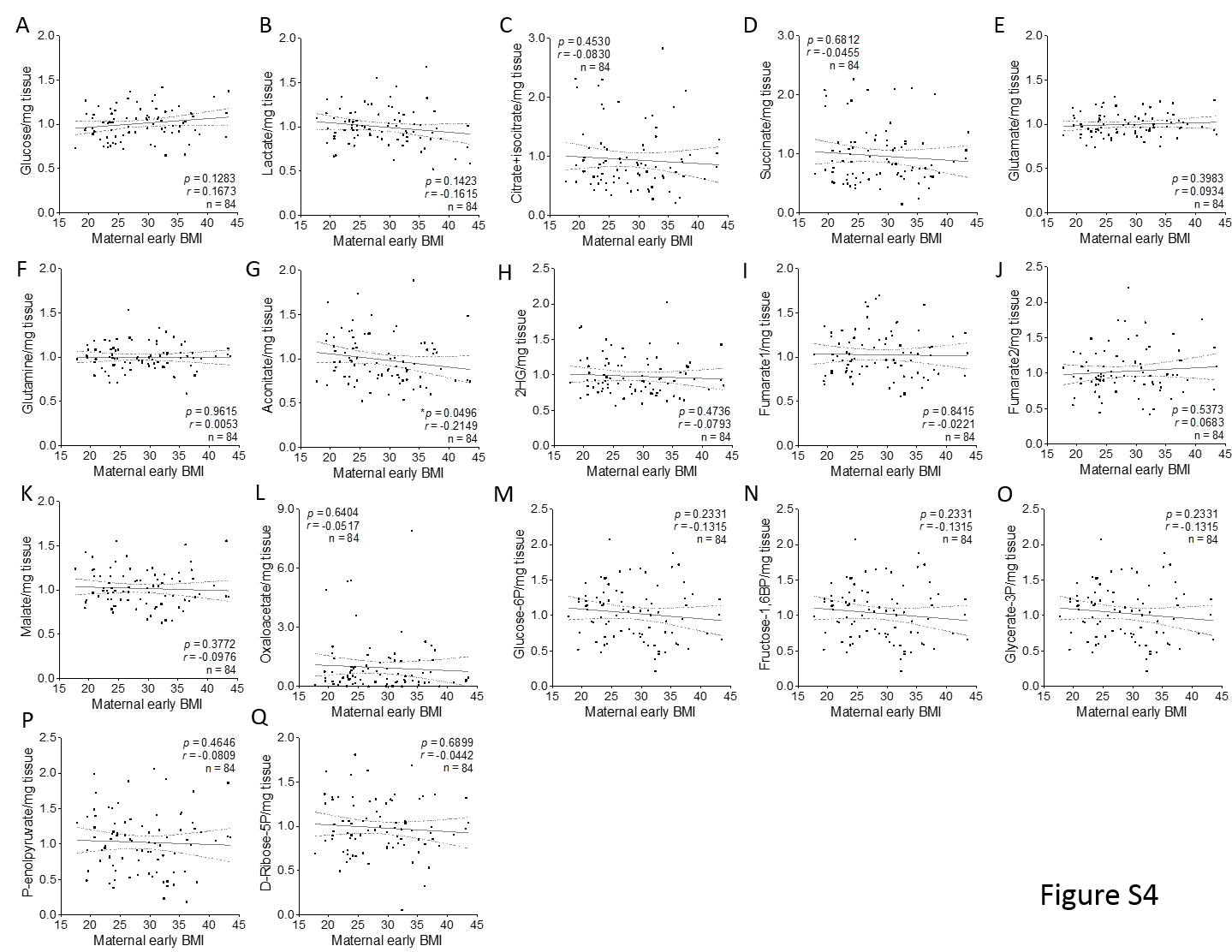

Supplement: S4 Fig — Correlations between placental levels of A. Glucose, B. Lactate, C. Citrate + isocitrate, D. Succinate, E. Glutamate, F. Glutamine, G. Aconitate, H. 2-hydroxyglutarate, I. Fumarate 1, J. Fumarate 2, K. Malate, L. Oxaloacetate, M. Glucose-6-phosphate, N. Fructose-1,6-biphosphate, O. Glycerate-3-phosphate, P. Phosphoenolpyruvate, Q. D-Ribose 5-phosphate and maternal BMI. The relations between continuous variables were evaluated by Spearman correlations. (TIF) [file pone.0186115.s004.tif]

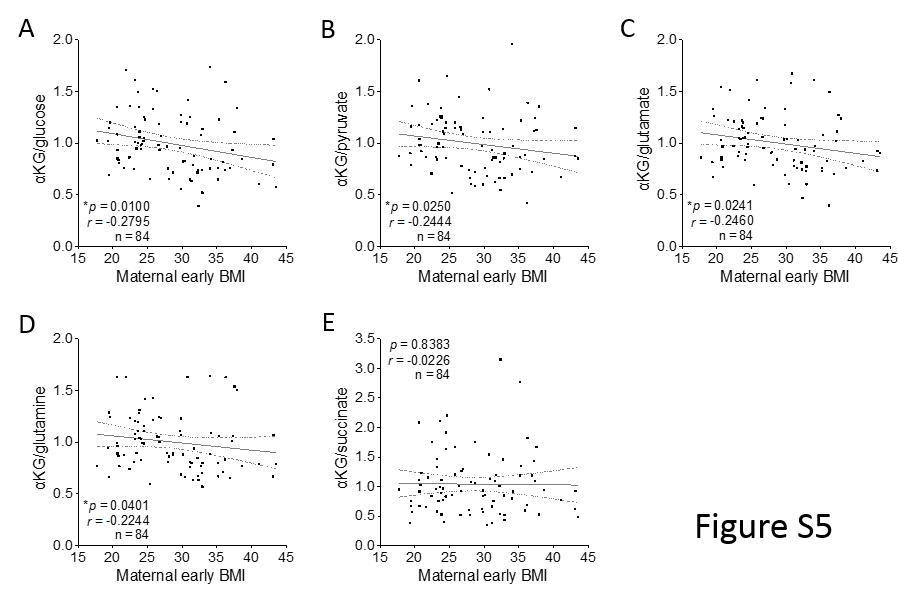

Supplement: S5 Fig — Correlations between placental levels of A. αKG/glucose, B. αKG/pyruvate, C. αKG/glutamate, D. αKG/glutamine, E. αKG/succinate and maternal BMI. The relations between continuous variables were evaluated by Spearman correlations. (TIF) [file pone.0186115.s005.tif]

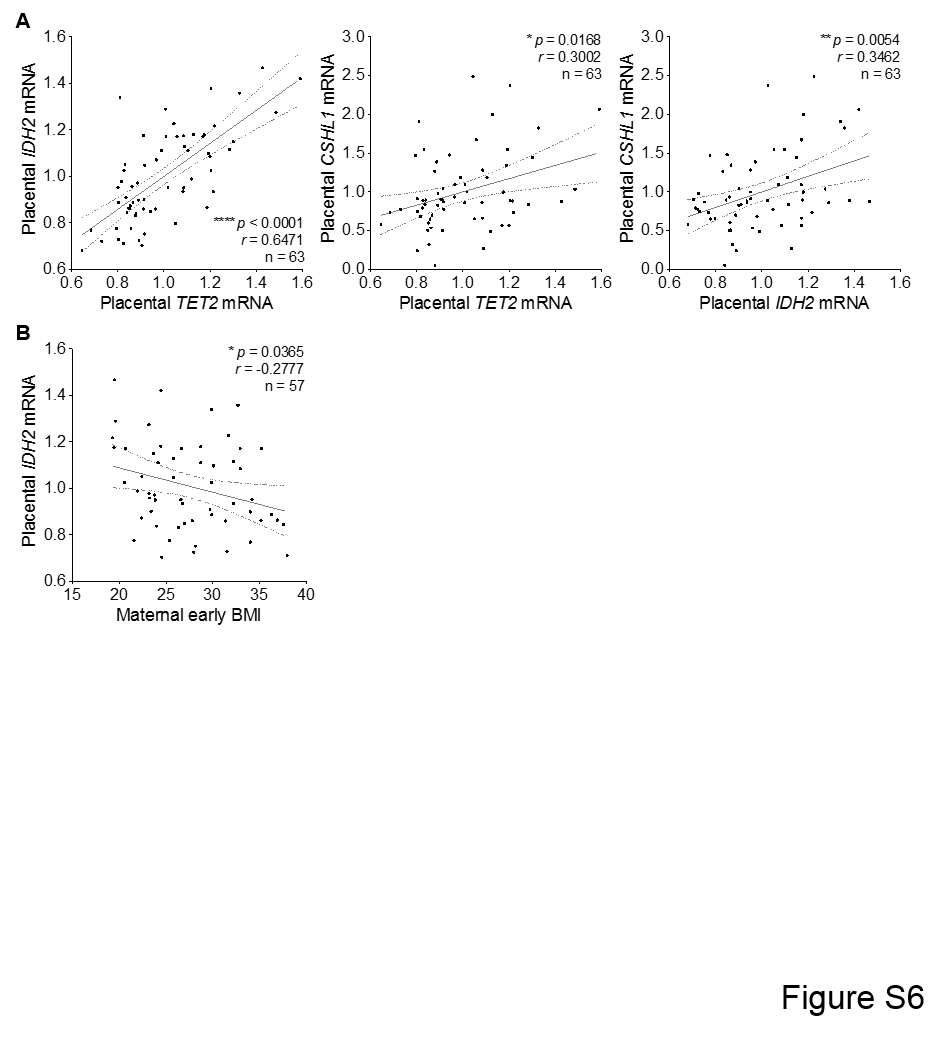

Supplement: S6 Fig — Correlations between TET2, IDH2, and CSHL1 mRNA levels (A) and between placental IDH2 levels and maternal BMI (B). Pre-pregnancy or first trimester BMI was used as maternal early BMI. The statistical significance was evaluated as indicated in the legend to Fig 4. (TIF) [file pone.0186115.s006.tif]

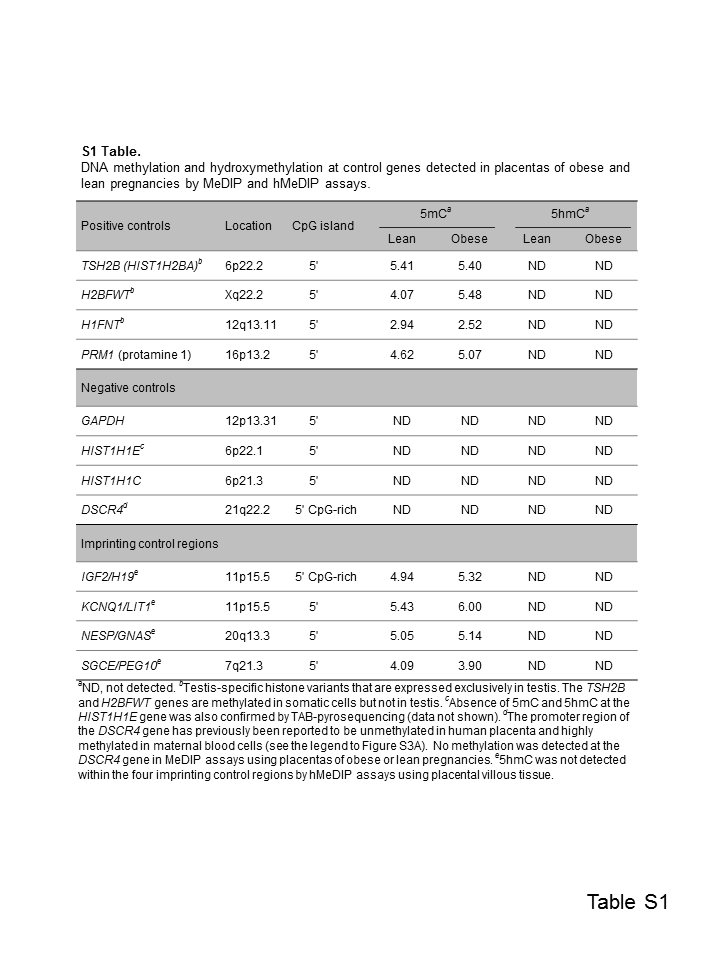

Supplement: S1 Table — (TIF) [file pone.0186115.s007.tif]

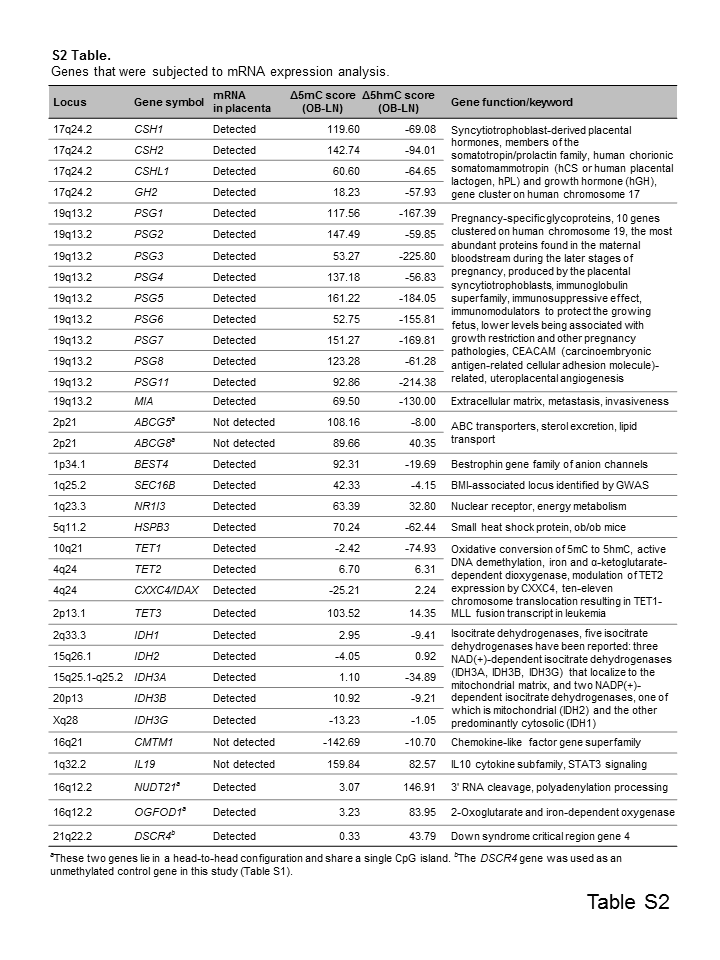

Supplement: S2 Table — (TIF) [file pone.0186115.s008.tif]

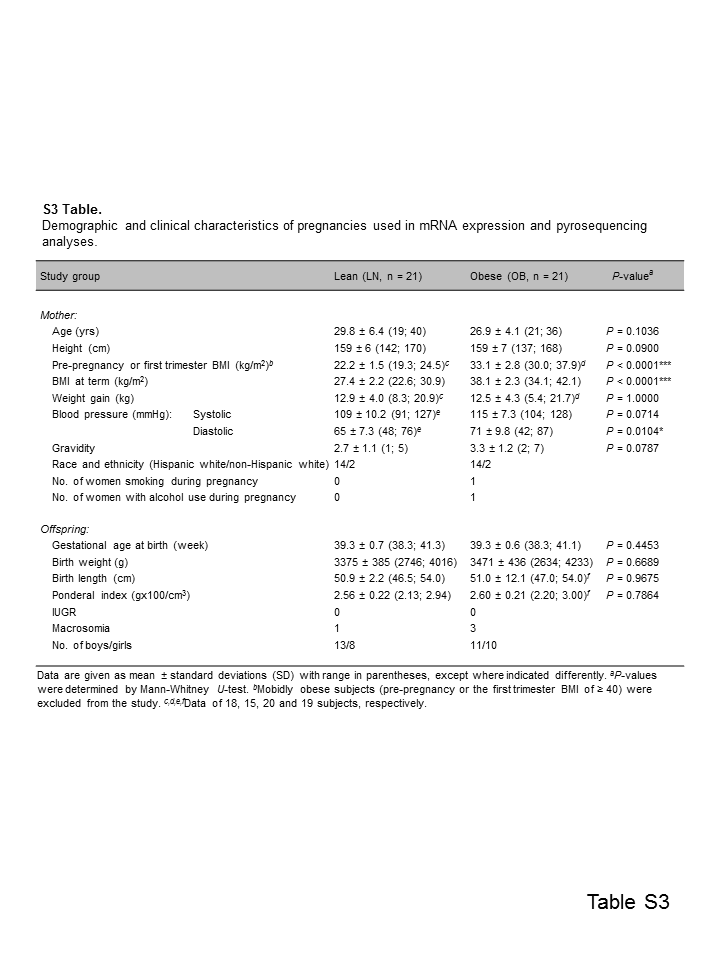

Supplement: S3 Table — (TIF) [file pone.0186115.s009.tif]

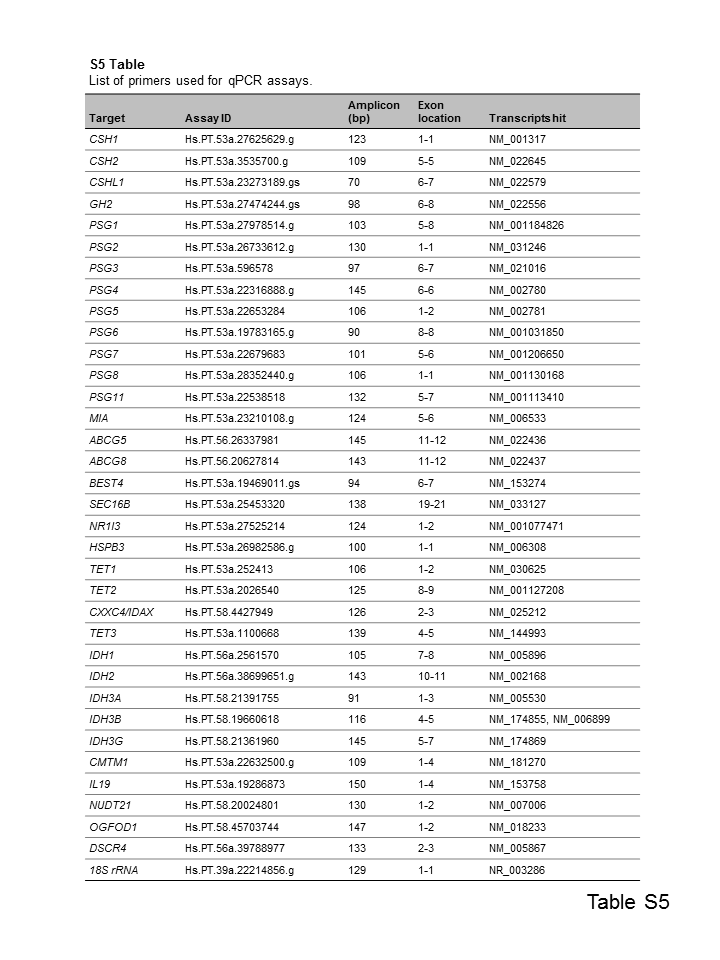

Supplement: S5 Table — (TIF) [file pone.0186115.s011.tif]
