# Supplementary material for: Alterations in the placental methylome with maternal obesity and evidence for metabolic regulation
Source: PLoS One. 2017 Oct 18;12(10):e0186115. doi: 10.1371/journal.pone.0186115 (PMC5646778; doi:10.1371/journal.pone.0186115)
Supplement: S4 Table — (PDF) [file pone.0186115.s010.pdf]

Annotation Cluster 1 Enrichment Score: 3.5186073788569745

Category	Term	Count	%	Fold Enrich	PValue	Genes	List Total	Pop Hits	Pop Total	Bonferroni	Benjamini	FDR
SP_PIR_KEYWORDS	Secreted	44	17.67068	2.03695	8.74E-06	MIA, CSH1, PPY, CSH2	246	1689	19235	0.00300	0.00150	0.01192
SP_PIR_KEYWORDS	disulfide bond	62	24.89960	1.65795	4.69E-05	CSH1, CSH2, CUZD1, A	246	2924	19235	0.01600	0.00536	0.06395
GOTERM_CC_FAT	GO:0005576~extracellular region	47	18.87550	1.77906	6.39E-05	CSH1, CSH2, IL16, LY8	168	2010	12782	0.01295	0.01295	0.08032
UP_SEQ_FEATURE	disulfide bond	57	22.89157	1.57099	4.47E-04	CSH1, CSH2, CUZD1, A	246	2819	19113	0.31534	0.17256	0.68860
SP_PIR_KEYWORDS	signal	62	24.89960	1.49164	8.73E-04	CSH1, CSH2, LY86, CU	246	3250	19235	0.25958	0.04886	1.18472
UP_SEQ_FEATURE	signal peptide	62	24.89960	1.48218	1.03E-03	CSH1, CSH2, LY86, CU	246	3250	19113	0.58130	0.15980	1.57543
UP_SEQ_FEATURE	glycosylation site:N-linked (GlcNAc...)	74	29.71888	1.39245	1.55E-03	SLC45A2, MCHR1, TRP	246	4129	19113	0.73246	0.15195	2.37622
SP_PIR_KEYWORDS	glycoprotein	74	29.71888	1.34000	4.36E-03	SLC45A2, MCHR1, TRP	246	4318	19235	0.77755	0.12772	5.78626

Annotation Cluster 2 Enrichment Score: 2.6263280765243415

Category	Term	Count	%	Fold Enrich	PValue	Genes	List Total	Pop Hits	Pop Total	Bonferroni	Benjamini	FDR
INTERPRO	IPR003598:Immunoglobulin subtype 2	12	4.81928	4.33405	1.06E-04	CILP, PSG2, MYBPHL, I	225	205	16659	0.05020	0.05020	0.15242
GOTERM_BP_FAT	GO:0007565~female pregnancy	9	3.61446	5.98290	1.26E-04	CSH1, CSH2, PSG8, PS	185	110	13528	0.13751	0.13751	0.20356
SMART	SM00408:IGc2	12	4.81928	4.05690	1.65E-04	CILP, PSG2, MYBPHL, I	131	205	9079	0.01899	0.01899	0.18837
SP_PIR_KEYWORDS	Immunoglobulin domain	17	6.82731	2.82819	3.65E-04	PSG2, CILP, SLAMF8, M	246	470	19235	0.11816	0.02484	0.49741
UP_SEQ_FEATURE	domain:Ig-like C2-type 1	10	4.01606	4.08922	7.94E-04	PSG8, PSG7, PSG6, PS	246	190	19113	0.49024	0.20117	1.22153
UP_SEQ_FEATURE	domain:Ig-like C2-type 2	10	4.01606	4.06781	8.23E-04	PSG8, PSG7, PSG6, PS	246	191	19113	0.50239	0.16011	1.26496
UP_SEQ_FEATURE	domain:Ig-like V-type	8	3.21285	5.01259	1.10E-03	PSG8, PSG7, PSG6, PS	246	124	19113	0.60744	0.14431	1.69111
INTERPRO	IPR007110:Immunoglobulin-like	17	6.82731	2.51234	1.24E-03	PSG2, CILP, SLAMF8, M	225	501	16659	0.45229	0.25992	1.76715
INTERPRO	IPR003599:Immunoglobulin subtype	13	5.22088	2.91673	1.76E-03	PSG2, MYBPHL, PSG1,	225	330	16659	0.57319	0.24708	2.49008
SMART	SM00409:IG	13	5.22088	2.73021	2.68E-03	PSG2, MYBPHL, PSG1,	131	330	9079	0.26745	0.14411	3.01462
INTERPRO	IPR013151:Immunoglobulin	9	3.61446	3.29881	6.09E-03	PSG8, PSG7, PSG6, PS	225	202	16659	0.94806	0.38918	8.38714
INTERPRO	IPR013106:Immunoglobulin V-set	10	4.01606	2.97349	6.67E-03	PSG8, PSG7, PSG6, PS	225	249	16659	0.96076	0.37034	9.14459
INTERPRO	IPR013783:Immunoglobulin-like fold	15	6.02410	2.00832	1.76E-02	PSG2, CILP, MYBPHL, I	225	553	16659	0.99982	0.65929	22.51702
UP_SEQ_FEATURE	short sequence motif:Cell attachment site	5	2.00803	4.41450	2.66E-02	PSG8, PSG7, PSG6, PS	246	88	19113	1.00000	0.78220	34.09875
SP_PIR_KEYWORDS	tandem repeat	4	1.60643	4.17019	7.07E-02	PSG8, PSG7, PSG6, PS	246	75	19235	1.00000	0.59389	63.23359
UP_SEQ_FEATURE	domain:Ig-like C2-type 3	4	1.60643	2.54738	2.06E-01	PSG8, PSG7, PSG6, PS	246	122	19113	1.00000	0.99685	97.19209

Annotation Cluster 3 Enrichment Score: 2.253436664848397

Category	Term	Count	%	Fold Enrich	PValue	Genes	List Total	Pop Hits	Pop Total	Bonferroni	Benjamini	FDR
SP_PIR_KEYWORDS	hormone	8	3.21285	7.18998	1.23E-04	CSH1, CSHL1, PPY, CS	246	87	19235	0.04133	0.01050	0.16724
INTERPRO	IPR001400:Somatotropin hormone	3	1.20482	37.02000	2.61E-03	CSH1, CSHL1, CSH2	225	6	16659	0.71711	0.27071	3.67070
GOTERM_MF_FAT	GO:0005179~hormone activity	7	2.81124	4.89239	3.02E-03	CSH1, CSHL1, PPY, CS	172	108	12983	0.69770	0.25850	4.12905
PIR_SUPERFAMILY	PIRSF001825:prolactin/lactogen/growth horm	3	1.20482	31.60684	3.51E-03	CSH1, CSHL1, CSH2	117	6	7396	0.36248	0.36248	4.00201
INTERPRO	IPR018116:Somatotropin hormone, conserve	3	1.20482	31.73143	3.62E-03	CSH1, CSHL1, CSH2	225	7	16659	0.82675	0.29574	5.05950
SP_PIR_KEYWORDS	amidation	3	1.20482	5.33121	1.08E-01	PPY, GNRH2, GAST	246	44	19235	1.00000	0.70721	78.95943
INTERPRO	IPR012351:Four-helical cytokine, core	3	1.20482	4.82870	1.27E-01	CSH1, CSHL1, CSH2	225	46	16659	1.00000	0.92786	85.72490

Annotation Cluster 4 Enrichment Score: 1.715342444632135

Category	Term	Count	%	Fold Enrich	PValue	Genes	List Total	Pop Hits	Pop Total	Bonferroni	Benjamini	FDR
GOTERM_MF_FAT	GO:0005529~sugar binding	11	4.41767	4.25799	2.58E-04	ASGR2, LGALS7B, GCK	172	195	12983	0.09673	0.09673	0.35793
SP_PIR_KEYWORDS	Lectin	9	3.61446	4.42591	1.00E-03	ASGR2, LGALS7B, FCN	246	159	19235	0.29220	0.04817	1.36113
GOTERM_MF_FAT	GO:0030246~carbohydrate binding	13	5.22088	2.77196	2.54E-03	LGALS9C, ITLN2, LGAL	172	354	12983	0.63419	0.28481	3.48246
INTERPRO	IPR008160:Collagen triple helix repeat	5	2.00803	4.40714	2.66E-02	FCN2, SFTPA2, COL12	225	84	16659	1.00000	0.72925	32.08816
SP_PIR_KEYWORDS	collagen	5	2.00803	4.11532	3.33E-02	FCN2, SFTPA2, COL12	246	95	19235	0.99999	0.42533	36.95493
UP_SEQ_FEATURE	domain:C-type lectin	4	1.60643	3.93393	8.11E-02	ASGR2, SFTPA2, COLE	246	79	19113	1.00000	0.92972	72.95527
INTERPRO	IPR001304:C-type lectin	4	1.60643	3.61171	9.84E-02	ASGR2, SFTPA2, COLE	225	82	16659	1.00000	0.88698	77.35280
INTERPRO	IPR018378:C-type lectin, conserved site	4	1.60643	3.61171	9.84E-02	ASGR2, SFTPA2, COLE	225	82	16659	1.00000	0.88698	77.35280
SMART	SM00034:CLECT	4	1.60643	3.38075	1.13E-01	ASGR2, SFTPA2, COLE	131	82	9079	1.00000	0.86255	74.49397
INTERPRO	IPR016186:C-type lectin-like	4	1.60643	3.11747	1.36E-01	ASGR2, SFTPA2, COLE	225	95	16659	1.00000	0.92740	87.72133

Annotation Cluster 5 Enrichment Score: 1.7127623933184326

Category	Term	Count	%	Fold Enrich	PValue	Genes	List Total	Pop Hits	Pop Total	Bonferroni	Benjamini	FDR
GOTERM_CC_FAT	GO:0016529~sarcoplasmic reticulum	4	1.60643	7.80342	1.42E-02	ANK1, PYGM, SRL, ART	168	39	12782	0.94579	0.62154	16.44613
GOTERM_CC_FAT	GO:0016528~sarcoplasm	4	1.60643	7.42276	1.62E-02	ANK1, PYGM, SRL, ART	168	41	12782	0.96456	0.56611	18.60616

SP_PIR_KEYWORDS	sarcoplasmic reticulum	3	1.20482	10.66242	3.16E-02	ANK1, SRL, ART1	246	22	19235	0.99998	0.44054	35.44134

Annotation Cluster 6 Enrichment Score: 1.5960334291609053

Category	Term	Count	%	Fold Enrich	PValue	Genes	List Total	Pop Hits	Pop Total	Bonferroni	Benjamini	FDR
SP_PIR_KEYWORDS	sh3 domain	8	3.21285	2.97871	1.83E-02	MIA, FGR, GRAPL, ARH	246	210	19235	0.99827	0.36516	22.29585
INTERPRO	IPR001452:Src homology-3 domain	8	3.21285	2.78085	2.54E-02	MIA, FGR, GRAPL, ARH	225	213	16659	1.00000	0.74890	30.81255
UP_SEQ_FEATURE	domain:SH3	7	2.81124	3.07269	2.68E-02	MIA, FGR, GRAPL, ARH	246	177	19113	1.00000	0.76296	34.30240
SMART	SM00326:SH3	8	3.21285	2.60302	3.32E-02	MIA, FGR, GRAPL, ARH	131	213	9079	0.97998	0.72846	31.93122

Annotation Cluster 7 Enrichment Score: 1.5958504189132505

Category	Term	Count	%	Fold Enrich	PValue	Genes	List Total	Pop Hits	Pop Total	Bonferroni	Benjamini	FDR
UP_SEQ_FEATURE	domain:EF-hand 3	7	2.81124	5.78581	1.33E-03	CIB4, MYL4, CALML4, C	246	94	19113	0.67568	0.14859	2.03291
PIR_SUPERFAMILY	PIRSF002350:calmodulin	4	1.60643	10.53561	5.98E-03	MYL4, CALML4, CALML	117	24	7396	0.53620	0.31897	6.73338
UP_SEQ_FEATURE	domain:EF-hand 4	5	2.00803	6.58433	6.91E-03	CALML4, CALML3, CET	246	59	19113	0.99720	0.41396	10.16679
UP_SEQ_FEATURE	domain:EF-hand 1	8	3.21285	3.41517	9.19E-03	CIB4, MYL4, CALML4, C	246	182	19113	0.99960	0.47918	13.30598
UP_SEQ_FEATURE	calcium-binding region:2	6	2.40964	4.05366	1.63E-02	CIB4, CALML3, CETN2,	246	115	19113	1.00000	0.65819	22.47345
UP_SEQ_FEATURE	calcium-binding region:1	6	2.40964	3.69977	2.32E-02	CIB4, CALML3, CETN2,	246	126	19113	1.00000	0.75914	30.47721
UP_SEQ_FEATURE	domain:EF-hand 2	7	2.81124	2.98827	3.01E-02	CIB4, MYL4, CALML4, C	246	182	19113	1.00000	0.76333	37.69596
SP_PIR_KEYWORDS	EF hand	4	1.60643	5.21274	4.11E-02	CALML3, CETN2, CAPN	246	60	19235	1.00000	0.45185	43.57146
INTERPRO	IPR011992:EF-Hand type	8	3.21285	2.50983	4.11E-02	CIB4, MYL4, CALML4, C	225	236	16659	1.00000	0.79022	45.18925
SP_PIR_KEYWORDS	calcium binding	5	2.00803	3.79568	4.27E-02	CALML3, CETN2, C1S,	246	103	19235	1.00000	0.45178	44.89398
INTERPRO	IPR002048:Calcium-binding EF-hand	6	2.40964	3.10657	4.40E-02	CIB4, CALML4, CALML3	225	143	16659	1.00000	0.78932	47.57330
SMART	SM00054:EFh	6	2.40964	2.90792	5.43E-02	CIB4, CALML4, CALML3	131	143	9079	0.99845	0.80168	47.08492
INTERPRO	IPR018249:EF-HAND 2	7	2.81124	2.41060	7.07E-02	CIB4, MYL4, CALML4, C	225	215	16659	1.00000	0.87616	65.06496
INTERPRO	IPR018248:EF hand	5	2.00803	2.84769	9.84E-02	CIB4, CALML3, CETN2,	225	130	16659	1.00000	0.89764	77.35139
INTERPRO	IPR018247:EF-HAND 1	6	2.40964	2.04719	1.67E-01	CIB4, CALML3, CETN2,	225	217	16659	1.00000	0.95754	92.71785

Annotation Cluster 8 Enrichment Score: 1.5464909536406366

Category	Term	Count	%	Fold Enrich	PValue	Genes	List Total	Pop Hits	Pop Total	Bonferroni	Benjamini	FDR
GOTERM_CC_FAT	GO:0005887~integral to plasma membrane	25	10.04016	1.60108	2.07E-02	KCNMB3, MCHR1, CLD	168	1188	12782	0.98609	0.57476	23.16745
GOTERM_CC_FAT	GO:0031226~intrinsic to plasma membrane	25	10.04016	1.56550	2.63E-02	KCNMB3, MCHR1, CLD	168	1215	12782	0.99567	0.59626	28.49747
GOTERM_CC_FAT	GO:0005886~plasma membrane	62	24.89960	1.24892	3.02E-02	MCHR1, CLDN4, MYO7	168	3777	12782	0.99807	0.54223	31.97678
GOTERM_CC_FAT	GO:0044459~plasma membrane part	39	15.66265	1.34691	3.96E-02	KCNMB3, MCHR1, CLD	168	2203	12782	0.99973	0.52690	39.79922

Annotation Cluster 9 Enrichment Score: 1.5333981715143548

Category	Term	Count	%	Fold Enrich	PValue	Genes	List Total	Pop Hits	Pop Total	Bonferroni	Benjamini	FDR
GOTERM_BP_FAT	GO:0007588~excretion	6	2.40964	7.56459	1.13E-03	TRPV1, CLCNKA, CLCN	185	58	13528	0.73275	0.48304	1.80117
GOTERM_BP_FAT	GO:0046903~secretion	10	4.01606	2.43748	2.16E-02	PPY, ANK1, TRPV1, LA	185	300	13528	1.00000	0.97421	29.71863
SP_PIR_KEYWORDS	ion transport	12	4.81928	1.62334	1.22E-01	KCNMB3, TTYH3, TRPV	246	578	19235	1.00000	0.70135	83.02044
GOTERM_BP_FAT	GO:0055085~transmembrane transport	11	4.41767	1.41365	2.47E-01	SLC2A9, FOLR2, TRPV1	185	569	13528	1.00000	0.99529	98.97092

Annotation Cluster 10 Enrichment Score: 1.4544487344066226

Category	Term	Count	%	Fold Enrich	PValue	Genes	List Total	Pop Hits	Pop Total	Bonferroni	Benjamini	FDR
GOTERM_BP_FAT	GO:0007155~cell adhesion	19	7.63052	1.98480	7.05E-03	MIA, CLDN18, THRA, CI	185	700	13528	0.99975	0.87391	10.78228
GOTERM_BP_FAT	GO:0022610~biological adhesion	19	7.63052	1.98197	7.16E-03	MIA, CLDN18, THRA, CI	185	701	13528	0.99978	0.81369	10.92803
SP_PIR_KEYWORDS	cell adhesion	9	3.61446	1.66758	1.73E-01	APP, SIGLEC8, ITGAE,	246	422	19235	1.00000	0.81218	92.47044
GOTERM_BP_FAT	GO:0016337~cell-cell adhesion	7	2.81124	1.85460	1.75E-01	LGALS7B, CLDN18, THI	185	276	13528	1.00000	0.99683	95.45285

Annotation Cluster 11 Enrichment Score: 1.3475161599045595

Category	Term	Count	%	Fold Enrich	PValue	Genes	List Total	Pop Hits	Pop Total	Bonferroni	Benjamini	FDR
UP_SEQ_FEATURE	topological domain:Extracellular	52	20.88353	1.48589	2.97E-03	MCHR1, SLC45A2, CLD	246	2719	19113	0.91978	0.24447	4.49769
UP_SEQ_FEATURE	topological domain:Cytoplasmic	60	24.09639	1.38166	6.44E-03	MCHR1, SLC45A2, CLD	246	3374	19113	0.99582	0.42171	9.50679
GOTERM_CC_FAT	GO:0005886~plasma membrane	62	24.89960	1.24892	3.02E-02	MCHR1, CLDN4, MYO7	168	3777	12782	0.99807	0.54223	31.97678
UP_SEQ_FEATURE	transmembrane region	75	30.12048	1.18655	6.21E-02	SLC45A2, MCHR1, CLD	246	4911	19113	1.00000	0.91544	62.89011
SP_PIR_KEYWORDS	transmembrane	75	30.12048	1.17923	6.96E-02	SLC45A2, MCHR1, CLD	246	4973	19235	1.00000	0.60093	62.60305
GOTERM_CC_FAT	GO:0016021~integral to membrane	78	31.32530	1.12035	1.25E-01	SLC45A2, MCHR1, CLD	168	5297	12782	1.00000	0.74284	81.25432
GOTERM_CC_FAT	GO:0031224~intrinsic to membrane	79	31.72691	1.09582	1.79E-01	SLC45A2, MCHR1, CLD	168	5485	12782	1.00000	0.77470	91.62848

SP_PIR_KEYWORDS	membrane	85	34.13655	1.06238	2.98E-01	SLC45A2, MCHR1, CLD	246	6256	19235	1.00000	0.87324	99.20332

Annotation Cluster 12 Enrichment Score: 1.3474421130431482

Category	Term	Count	%	Fold Enrich	PValue	Genes	List Total	Pop Hits	Pop Total	Bonferroni	Benjamini	FDR
GOTERM_CC_FAT	GO:0005929~cilium	6	2.40964	3.53876	2.70E-02	GNAT1, MCHR1, APP, (	168	129	12782	0.99627	0.55010	29.15228
GOTERM_CC_FAT	GO:0044463~cell projection part	8	3.21285	2.60114	3.40E-02	GNAT1, APP, DNALI1, T	168	234	12782	0.99915	0.54393	35.30901
GOTERM_CC_FAT	GO:0042995~cell projection	16	6.42570	1.74653	3.94E-02	FGD2, GNAT1, MCHR1,	168	697	12782	0.99972	0.55912	39.63906
GOTERM_CC_FAT	GO:0044441~cilium part	3	1.20482	5.18750	1.13E-01	GNAT1, APP, CROCC	168	44	12782	1.00000	0.74156	77.71535

Annotation Cluster 13 Enrichment Score: 1.1965039354208902

Category	Term	Count	%	Fold Enrich	PValue	Genes	List Total	Pop Hits	Pop Total	Bonferroni	Benjamini	FDR
SP_PIR_KEYWORDS	hydroxyproline	4	1.60643	8.45309	1.15E-02	FCN2, SFTPA2, COL12/	246	37	19235	0.98148	0.28280	14.63053
SP_PIR_KEYWORDS	hydroxylation	5	2.00803	5.50641	1.28E-02	FCN2, SFTPA2, COL12/	246	71	19235	0.98820	0.28928	16.14188
INTERPRO	IPR008160:Collagen triple helix repeat	5	2.00803	4.40714	2.66E-02	FCN2, SFTPA2, COL12/	225	84	16659	1.00000	0.72925	32.08816
SP_PIR_KEYWORDS	collagen	5	2.00803	4.11532	3.33E-02	FCN2, SFTPA2, COL12/	246	95	19235	0.99999	0.42533	36.95493
UP_SEQ_FEATURE	domain:Collagen-like	3	1.20482	6.47459	7.76E-02	FCN2, SFTPA2, SFTPA:	246	36	19113	1.00000	0.94235	71.32277
GOTERM_CC_FAT	GO:0005578~proteinaceous extracellular mat	7	2.81124	1.66432	2.41E-01	MMP21, NAV2, SFTPA2	168	320	12782	1.00000	0.79974	96.88639
GOTERM_CC_FAT	GO:0031012~extracellular matrix	7	2.81124	1.54372	2.97E-01	MMP21, NAV2, SFTPA2	168	345	12782	1.00000	0.84122	98.80123
SP_PIR_KEYWORDS	extracellular matrix	5	2.00803	1.62222	3.69E-01	SFTPA2, CILP, COL12A	246	241	19235	1.00000	0.91247	99.81236

Annotation Cluster 14 Enrichment Score: 1.128278685377582

Category	Term	Count	%	Fold Enrich	PValue	Genes	List Total	Pop Hits	Pop Total	Bonferroni	Benjamini	FDR
GOTERM_BP_FAT	GO:0006952~defense response	18	7.22892	2.14022	4.35E-03	SPACA3, SP100, FGR,	185	615	13528	0.99391	0.81738	6.78515
GOTERM_BP_FAT	GO:0002526~acute inflammatory response	5	2.00803	3.73083	4.46E-02	ORM1, TRPV1, FCN2, S	185	98	13528	1.00000	0.99522	52.09416
SP_PIR_KEYWORDS	acute phase	3	1.20482	8.08873	5.24E-02	ORM1, SERPINA1, C1S	246	29	19235	1.00000	0.50919	51.99156
GOTERM_BP_FAT	GO:0006953~acute-phase response	3	1.20482	5.48432	1.03E-01	ORM1, TRPV1, SERPIN	185	40	13528	1.00000	0.99685	82.53733
GOTERM_BP_FAT	GO:0006954~inflammatory response	7	2.81124	1.57499	2.78E-01	ORM1, TRPV1, FCN2, L	185	325	13528	1.00000	0.99632	99.47573
GOTERM_BP_FAT	GO:0009611~response to wounding	8	3.21285	1.10376	5.86E-01	ORM1, TRPV1, FCN2, L	185	530	13528	1.00000	0.99905	99.99993

Annotation Cluster 15 Enrichment Score: 1.1233807915335263

Category	Term	Count	%	Fold Enrich	PValue	Genes	List Total	Pop Hits	Pop Total	Bonferroni	Benjamini	FDR
UP_SEQ_FEATURE	domain:MAGE	3	1.20482	7.28392	6.31E-02	MAGEB1, MAGED4, MA	246	32	19113	1.00000	0.90964	63.52409
INTERPRO	IPR002190:MAGE protein	3	1.20482	6.34629	8.02E-02	MAGEB1, MAGED4, MA	225	35	16659	1.00000	0.88110	69.82859
SP_PIR_KEYWORDS	tumor antigen	3	1.20482	6.17298	8.42E-02	MAGEB1, MAGED4, MA	246	38	19235	1.00000	0.63545	69.89587

Annotation Cluster 16 Enrichment Score: 1.0994139339883393

Category	Term	Count	%	Fold Enrich	PValue	Genes	List Total	Pop Hits	Pop Total	Bonferroni	Benjamini	FDR
UP_SEQ_FEATURE	repeat:ANK 5	6	2.40964	3.55856	2.69E-02	ANK1, POTEH, TRPV1,	246	131	19113	1.00000	0.74349	34.41942
UP_SEQ_FEATURE	repeat:ANK 3	7	2.81124	2.80343	3.93E-02	ANK1, POTEH, TRPV1,	246	194	19113	1.00000	0.81707	46.18734
UP_SEQ_FEATURE	repeat:ANK 4	6	2.40964	2.95045	5.31E-02	ANK1, POTEH, TRPV1,	246	158	19113	1.00000	0.88940	56.97540
UP_SEQ_FEATURE	repeat:ANK 1	7	2.81124	2.35440	7.76E-02	ANK1, POTEH, TRPV1,	246	231	19113	1.00000	0.93540	71.32783
UP_SEQ_FEATURE	repeat:ANK 2	7	2.81124	2.34425	7.88E-02	ANK1, POTEH, TRPV1,	246	232	19113	1.00000	0.93135	71.92679
SP_PIR_KEYWORDS	ank repeat	7	2.81124	2.28057	8.72E-02	ANK1, POTEH, TRPV1,	246	240	19235	1.00000	0.63662	71.19064
INTERPRO	IPR002110:Ankyrin	7	2.81124	2.17765	1.02E-01	ANK1, POTEH, TRPV1,	225	238	16659	1.00000	0.88605	78.64508
SMART	SM00248:ANK	7	2.81124	2.03839	1.26E-01	ANK1, POTEH, TRPV1,	131	238	9079	1.00000	0.85876	78.56281
UP_SEQ_FEATURE	repeat:ANK 6	3	1.20482	2.53354	3.30E-01	ANK1, POTEH, TRPV1	246	92	19113	1.00000	0.99956	99.79799

Annotation Cluster 17 Enrichment Score: 1.0887988776191155

Category	Term	Count	%	Fold Enrich	PValue	Genes	List Total	Pop Hits	Pop Total	Bonferroni	Benjamini	FDR
INTERPRO	IPR001715:Calponin-like actin-binding	4	1.60643	4.23086	6.82E-02	SYNE2, ARHGEF7, NAV	225	70	16659	1.00000	0.88190	63.66170
SMART	SM00033:CH	4	1.60643	3.96031	7.87E-02	SYNE2, ARHGEF7, NAV	131	70	9079	0.99993	0.85059	60.73573
UP_SEQ_FEATURE	domain:CH	3	1.20482	5.54965	1.01E-01	ARHGEF7, NAV2, SMTN	246	42	19113	1.00000	0.96016	80.71698

Annotation Cluster 18 Enrichment Score: 0.9847830836704512

Category	Term	Count	%	Fold Enrich	PValue	Genes	List Total	Pop Hits	Pop Total	Bonferroni	Benjamini	FDR
GOTERM_MF_FAT	GO:0003774~motor activity	6	2.40964	3.18940	3.97E-02	KIF1A, DNALI1, MYL4, N	172	142	12983	1.00000	0.89827	43.10029
GOTERM_CC_FAT	GO:0030286~dynein complex	3	1.20482	6.71324	7.25E-02	DNALI1, DNHD1, DCTN	168	34	12782	1.00000	0.64079	61.19590

GOTERM_CC_FAT	GO:0005875~microtubule associated comple:	3	1.20482	2.23775	3.86E-01	DNALI1, DNHD1, DCTN	168	102	12782	1.00000	0.88497	99.78273

Annotation Cluster 19 Enrichment Score: 0.9171960463158848

Category	Term	Count	%	Fold Enrich	PValue	Genes	List Total	Pop Hits	Pop Total	Bonferroni	Benjamini	FDR
INTERPRO	IPR002035:von Willebrand factor, type A	4	1.60643	3.74886	9.04E-02	ITIH1, ITGAE, COL12A1	225	79	16659	1.00000	0.88743	74.29394
SMART	SM00327:VWA	4	1.60643	3.50913	1.04E-01	ITIH1, ITGAE, COL12A1	131	79	9079	1.00000	0.87993	71.37410
UP_SEQ_FEATURE	domain:VWFA	3	1.20482	3.75944	1.89E-01	ITIH1, ITGAE, CACNA2I	246	62	19113	1.00000	0.99608	96.06283

Annotation Cluster 20 Enrichment Score: 0.866276959238014

Category	Term	Count	%	Fold Enrich	PValue	Genes	List Total	Pop Hits	Pop Total	Bonferroni	Benjamini	FDR
GOTERM_BP_FAT	GO:0010038~response to metal ion	5	2.00803	2.83428	9.93E-02	KCNMB3, TNNT2, SERF	185	129	13528	1.00000	0.99704	81.44738
GOTERM_BP_FAT	GO:0010035~response to inorganic substanc	6	2.40964	2.14022	1.48E-01	KCNMB3, TNNT2, SERF	185	205	13528	1.00000	0.99803	92.38241
GOTERM_BP_FAT	GO:0051592~response to calcium ion	3	1.20482	3.98860	1.72E-01	KCNMB3, TNNT2, CAPN	185	55	13528	1.00000	0.99743	95.21212

Annotation Cluster 21 Enrichment Score: 0.829324157367664

Category	Term	Count	%	Fold Enrich	PValue	Genes	List Total	Pop Hits	Pop Total	Bonferroni	Benjamini	FDR
INTERPRO	IPR000832:GPCR, family 2, secretin-like	3	1.20482	4.82870	1.27E-01	GPR133, GPR123, GPR	225	46	16659	1.00000	0.92786	85.72490
INTERPRO	IPR017983:GPCR, family 2, secretin-like, con	3	1.20482	4.62750	1.36E-01	GPR133, GPR123, GPR	225	48	16659	1.00000	0.93430	87.71243
INTERPRO	IPR017981:GPCR, family 2-like	3	1.20482	3.76475	1.88E-01	GPR133, GPR123, GPR	225	59	16659	1.00000	0.95727	94.96138

Annotation Cluster 22 Enrichment Score: 0.8241206941362258

Category	Term	Count	%	Fold Enrich	PValue	Genes	List Total	Pop Hits	Pop Total	Bonferroni	Benjamini	FDR
SP_PIR_KEYWORDS	muscle protein	4	1.60643	5.21274	4.11E-02	TNNT2, MYL4, MYBPH,	246	60	19235	1.00000	0.45185	43.57146
GOTERM_BP_FAT	GO:0006936~muscle contraction	5	2.00803	2.38968	1.54E-01	TNNT2, MYL4, TRPV1, I	185	153	13528	1.00000	0.99783	93.30075
GOTERM_BP_FAT	GO:0003012~muscle system process	5	2.00803	2.17632	1.94E-01	TNNT2, MYL4, TRPV1, I	185	168	13528	1.00000	0.99676	96.89889
GOTERM_BP_FAT	GO:0044057~regulation of system process	6	2.40964	1.41989	4.11E-01	TNNT2, MYL4, THRA, P	185	309	13528	1.00000	0.99740	99.98017

Annotation Cluster 23 Enrichment Score: 0.8089374355690231

Category	Term	Count	%	Fold Enrich	PValue	Genes	List Total	Pop Hits	Pop Total	Bonferroni	Benjamini	FDR
GOTERM_BP_FAT	GO:0051606~detection of stimulus	5	2.00803	3.09849	7.73E-02	KCNMB3, GNAT1, GCK	185	118	13528	1.00000	0.99812	72.66338
GOTERM_BP_FAT	GO:0050906~detection of stimulus involved ir	3	1.20482	6.09369	8.58E-02	GNAT1, TRPV1, RHO	185	36	13528	1.00000	0.99859	76.44622
GOTERM_BP_FAT	GO:0009628~response to abiotic stimulus	6	2.40964	1.19224	5.64E-01	GNAT1, APP, TRPV1, P	185	368	13528	1.00000	0.99908	99.99985

Annotation Cluster 24 Enrichment Score: 0.808827839676675

Category	Term	Count	%	Fold Enrich	PValue	Genes	List Total	Pop Hits	Pop Total	Bonferroni	Benjamini	FDR
GOTERM_MF_FAT	GO:0008092~cytoskeletal protein binding	11	4.41767	1.64744	1.29E-01	TNS4, TNNT2, SHROON	172	504	12983	1.00000	0.99301	85.40633
GOTERM_MF_FAT	GO:0003779~actin binding	8	3.21285	1.85233	1.40E-01	TNS4, TNNT2, SHROON	172	326	12983	1.00000	0.98959	87.65439
SP_PIR_KEYWORDS	actin-binding	6	2.40964	1.89938	2.08E-01	TNS4, SHROOM1, DNA	246	247	19235	1.00000	0.83173	95.84303

Annotation Cluster 25 Enrichment Score: 0.7396954150941644

Category	Term	Count	%	Fold Enrich	PValue	Genes	List Total	Pop Hits	Pop Total	Bonferroni	Benjamini	FDR
GOTERM_BP_FAT	GO:0051928~positive regulation of calcium io	3	1.20482	6.64767	7.38E-02	MCHR1, P2RX3, LACRT	185	33	13528	1.00000	0.99835	70.94046
GOTERM_BP_FAT	GO:0010959~regulation of metal ion transpor	4	1.60643	3.61108	9.81E-02	MCHR1, GCK, P2RX3, L	185	81	13528	1.00000	0.99827	81.07149
GOTERM_BP_FAT	GO:0043270~positive regulation of ion transp	3	1.20482	5.22317	1.11E-01	MCHR1, P2RX3, LACRT	185	42	13528	1.00000	0.99683	85.08033
GOTERM_BP_FAT	GO:0043269~regulation of ion transport	4	1.60643	2.95452	1.52E-01	MCHR1, GCK, P2RX3, L	185	99	13528	1.00000	0.99802	92.99689
GOTERM_BP_FAT	GO:0051924~regulation of calcium ion transp	3	1.20482	3.17932	2.41E-01	MCHR1, P2RX3, LACRT	185	69	13528	1.00000	0.99620	98.83496
GOTERM_BP_FAT	GO:0051050~positive regulation of transport	5	2.00803	1.63956	3.60E-01	MCHR1, SPACA3, GCK	185	223	13528	1.00000	0.99676	99.92429
GOTERM_BP_FAT	GO:0019932~second-messenger-mediated si	4	1.60643	1.24467	6.23E-01	MCHR1, PTGIR, GCK, L	185	235	13528	1.00000	0.99943	99.99999

Annotation Cluster 26 Enrichment Score: 0.7006199217622314

Category	Term	Count	%	Fold Enrich	PValue	Genes	List Total	Pop Hits	Pop Total	Bonferroni	Benjamini	FDR
GOTERM_CC_FAT	GO:0005774~vacuolar membrane	3	1.20482	4.07589	1.66E-01	ATP6V1B1, ATP6V0A4,	168	56	12782	1.00000	0.75938	89.80240
GOTERM_CC_FAT	GO:0044437~vacuolar part	3	1.20482	3.56641	2.04E-01	ATP6V1B1, ATP6V0A4,	168	64	12782	1.00000	0.77664	94.29782
GOTERM_CC_FAT	GO:0005773~vacuole	6	2.40964	1.81151	2.34E-01	SPACA3, PLA2G4F, ATI	168	252	12782	1.00000	0.79776	96.49053

Annotation Cluster 27 Enrichment Score: 0.6819235678949702

Category	Term	Count	%	Fold Enrich	PValue	Genes	List Total	Pop Hits	Pop Total	Bonferroni	Benjamini	FDR

GOTERM_BP_FAT	GO:0016052~carbohydrate catabolic process	5	2.00803	3.35433	6.14E-02	SPACA3, GCK, LDHAL6	185	109	13528	1.00000	0.99793	64.00286
GOTERM_BP_FAT	GO:0046164~alcohol catabolic process	4	1.60643	3.61108	9.81E-02	GCK, LDHAL6B, TKTL1,	185	81	13528	1.00000	0.99827	81.07149
GOTERM_BP_FAT	GO:0044275~cellular carbohydrate catabolic	4	1.60643	3.44114	1.09E-01	GCK, LDHAL6B, PYGM,	185	85	13528	1.00000	0.99724	84.52911
GOTERM_BP_FAT	GO:0006007~glucose catabolic process	3	1.20482	3.78229	1.87E-01	GCK, LDHAL6B, TKTL1	185	58	13528	1.00000	0.99724	96.40902
GOTERM_BP_FAT	GO:0019320~hexose catabolic process	3	1.20482	3.17932	2.41E-01	GCK, LDHAL6B, TKTL1	185	69	13528	1.00000	0.99620	98.83496
GOTERM_BP_FAT	GO:0046365~monosaccharide catabolic proc	3	1.20482	3.08976	2.51E-01	GCK, LDHAL6B, TKTL1	185	71	13528	1.00000	0.99539	99.06105
GOTERM_BP_FAT	GO:0006006~glucose metabolic process	4	1.60643	1.91175	3.42E-01	GCK, LDHAL6B, PYGM,	185	153	13528	1.00000	0.99620	99.88331
GOTERM_BP_FAT	GO:0019318~hexose metabolic process	4	1.60643	1.52342	4.88E-01	GCK, LDHAL6B, PYGM,	185	192	13528	1.00000	0.99810	99.99794
GOTERM_BP_FAT	GO:0005996~monosaccharide metabolic proc	4	1.60643	1.31756	5.85E-01	GCK, LDHAL6B, PYGM,	185	222	13528	1.00000	0.99908	99.99993

Annotation Cluster 28 Enrichment Score: 0.6771027625792073

Category	Term	Count	%	Fold Enrich	PValue	Genes	List Total	Pop Hits	Pop Total	Bonferroni	Benjamini	FDR
INTERPRO	IPR006187:Claudin	3	1.20482	11.10600	2.92E-02	CLDN18, CLDN4, CLDN	225	20	16659	1.00000	0.72815	34.57964
INTERPRO	IPR017974:Claudin, conserved site	3	1.20482	10.57714	3.20E-02	CLDN18, CLDN4, CLDN	225	21	16659	1.00000	0.73019	37.22319
GOTERM_BP_FAT	GO:0016338~calcium-independent cell-cell ad	3	1.20482	9.97150	3.56E-02	CLDN18, CLDN4, CLDN	185	22	13528	1.00000	0.99097	44.20888
INTERPRO	IPR004031:PMP-22/EMP/MP20/Claudin	3	1.20482	6.17000	8.42E-02	CLDN18, CLDN4, CLDN	225	36	16659	1.00000	0.88095	71.65163
KEGG_PATHWAY	hsa04530:Tight junction	5	2.00803	2.91906	8.73E-02	CLDN18, EPB41L1, CLD	65	134	5085	0.99920	0.99920	61.95984
GOTERM_BP_FAT	GO:0016337~cell-cell adhesion	7	2.81124	1.85460	1.75E-01	LGALS7B, CLDN18, THI	185	276	13528	1.00000	0.99683	95.45285
SP_PIR_KEYWORDS	Tight junction	3	1.20482	3.84546	1.82E-01	CLDN18, CLDN4, CLDN	246	61	19235	1.00000	0.81523	93.57851
GOTERM_CC_FAT	GO:0070160~occluding junction	3	1.20482	3.12671	2.47E-01	CLDN18, CLDN4, CLDN	168	73	12782	1.00000	0.79971	97.17942
GOTERM_CC_FAT	GO:0005923~tight junction	3	1.20482	3.12671	2.47E-01	CLDN18, CLDN4, CLDN	168	73	12782	1.00000	0.79971	97.17942
GOTERM_CC_FAT	GO:0043296~apical junction complex	3	1.20482	2.30556	3.72E-01	CLDN18, CLDN4, CLDN	168	99	12782	1.00000	0.88426	99.71157
GOTERM_CC_FAT	GO:0016327~apicolateral plasma membrane	3	1.20482	2.23775	3.86E-01	CLDN18, CLDN4, CLDN	168	102	12782	1.00000	0.88497	99.78273
KEGG_PATHWAY	hsa04670:Leukocyte transendothelial migratic	3	1.20482	1.98892	4.40E-01	CLDN18, CLDN4, CLDN	65	118	5085	1.00000	0.99988	99.78346
KEGG_PATHWAY	hsa04514:Cell adhesion molecules (CAMs)	3	1.20482	1.77797	4.99E-01	CLDN18, CLDN4, CLDN	65	132	5085	1.00000	0.99987	99.93274
SP_PIR_KEYWORDS	cell junction	6	2.40964	1.17581	5.77E-01	TNS4, CLDN18, LIMS2,	246	399	19235	1.00000	0.97292	99.99920
GOTERM_CC_FAT	GO:0030054~cell junction	7	2.81124	1.02815	6.75E-01	TNS4, CLDN18, LIMS2,	168	518	12782	1.00000	0.98213	99.99993
GOTERM_CC_FAT	GO:0005911~cell-cell junction	3	1.20482	1.20132	7.13E-01	CLDN18, CLDN4, CLDN	168	190	12782	1.00000	0.98572	99.99999
GOTERM_MF_FAT	GO:0042802~identical protein binding	8	3.21285	0.94353	7.45E-01	APP, CLDN18, SP100, T	172	640	12983	1.00000	0.99968	########

Annotation Cluster 29 Enrichment Score: 0.6657870485863008

Category	Term	Count	%	Fold Enrich	PValue	Genes	List Total	Pop Hits	Pop Total	Bonferroni	Benjamini	FDR
INTERPRO	IPR003054:Type II keratin	3	1.20482	7.93286	5.41E-02	KRT82, KRT74, FCN2	225	28	16659	1.00000	0.83394	54.95957
INTERPRO	IPR016044:Filament	4	1.60643	4.11333	7.29E-02	KRT82, KRT37, KRT74,	225	72	16659	1.00000	0.86930	66.20205
SP_PIR_KEYWORDS	Intermediate filament	4	1.60643	4.00980	7.75E-02	KRT82, KRT37, KRT74,	246	78	19235	1.00000	0.61615	66.75139
UP_SEQ_FEATURE	region of interest:Linker 12	3	1.20482	3.64196	1.98E-01	KRT82, KRT37, KRT74	246	64	19113	1.00000	0.99655	96.70591
UP_SEQ_FEATURE	region of interest:Coil 2	3	1.20482	3.64196	1.98E-01	KRT82, KRT37, KRT74	246	64	19113	1.00000	0.99655	96.70591
UP_SEQ_FEATURE	region of interest:Coil 1A	3	1.20482	3.32979	2.26E-01	KRT82, KRT37, KRT74	246	70	19113	1.00000	0.99720	98.10610
UP_SEQ_FEATURE	region of interest:Linker 1	3	1.20482	3.32979	2.26E-01	KRT82, KRT37, KRT74	246	70	19113	1.00000	0.99720	98.10610
UP_SEQ_FEATURE	region of interest:Coil 1B	3	1.20482	3.32979	2.26E-01	KRT82, KRT37, KRT74	246	70	19113	1.00000	0.99720	98.10610
UP_SEQ_FEATURE	region of interest:Rod	3	1.20482	3.28289	2.31E-01	KRT82, KRT37, KRT74	246	71	19113	1.00000	0.99715	98.27746
UP_SEQ_FEATURE	region of interest:Head	3	1.20482	3.19295	2.40E-01	KRT82, KRT37, KRT74	246	73	19113	1.00000	0.99747	98.57811
PIR_SUPERFAMILY	PIRSF002282:cytoskeletal keratin	3	1.20482	3.10887	2.48E-01	KRT82, KRT37, KRT74	117	61	7396	1.00000	0.99999	96.35422
UP_SEQ_FEATURE	region of interest:Tail	3	1.20482	3.10780	2.50E-01	KRT82, KRT37, KRT74	246	75	19113	1.00000	0.99699	98.82953
INTERPRO	IPR018039:Intermediate filament protein, con	3	1.20482	3.08500	2.52E-01	KRT82, KRT37, KRT74	225	72	16659	1.00000	0.97997	98.45421
INTERPRO	IPR001664:Intermediate filament protein	3	1.20482	3.04274	2.57E-01	KRT82, KRT37, FCN2	225	73	16659	1.00000	0.97961	98.59568
GOTERM_CC_FAT	GO:0045095~keratin filament	3	1.20482	2.59375	3.20E-01	KRT82, KRT74, FCN2	168	88	12782	1.00000	0.85287	99.21193
GOTERM_CC_FAT	GO:0005882~intermediate filament	4	1.60643	1.66302	4.29E-01	KRT82, KRT37, KRT74,	168	183	12782	1.00000	0.90319	99.91348
GOTERM_CC_FAT	GO:0045111~intermediate filament cytoskelet	4	1.60643	1.62745	4.43E-01	KRT82, KRT37, KRT74,	168	187	12782	1.00000	0.90369	99.93617
SP_PIR_KEYWORDS	keratin	3	1.20482	1.62898	5.50E-01	KRT82, KRT37, KRT74	246	144	19235	1.00000	0.96902	99.99813

Annotation Cluster 30 Enrichment Score: 0.6519596368796196

Category	Term	Count	%	Fold Enrich	PValue	Genes	List Total	Pop Hits	Pop Total	Bonferroni	Benjamini	FDR
GOTERM_MF_FAT	GO:0005509~calcium ion binding	26	10.44177	2.13552	4.15E-04	MYL4, TRPV1, ITGAE, S	172	919	12983	0.15116	0.07867	0.57598
SP_PIR_KEYWORDS	calcium	22	8.83534	2.14222	1.50E-03	TRPV1, ITGAE, SRL, CE	246	803	19235	0.40250	0.05562	2.02160
GOTERM_MF_FAT	GO:0043167~ion binding	53	21.28514	0.94331	7.61E-01	APOBEC2, THRA, TRPV	172	4241	12983	1.00000	0.99973	########
GOTERM_MF_FAT	GO:0046872~metal ion binding	51	20.48193	0.92986	7.96E-01	APOBEC2, THRA, TRPV	172	4140	12983	1.00000	0.99982	########

GOTERM_MF_FAT	GO:0043169~cation binding	51	20.48193	0.92118	8.19E-01	APOBEC2, THRA, TRPV	172	4179	12983	1.00000	0.99988	########
SP_PIR_KEYWORDS	metal-binding	28	11.24498	0.73666	9.82E-01	FGD2, APOBEC2, LIMS	246	2972	19235	1.00000	1.00000	########
SP_PIR_KEYWORDS	zinc	14	5.62249	0.50008	1.00E+00	APOBEC2, FGD2, BRD1	246	2189	19235	1.00000	1.00000	########
GOTERM_MF_FAT	GO:0046914~transition metal ion binding	20	8.03213	0.54207	1.00E+00	FGD2, APOBEC2, BRD1	172	2785	12983	1.00000	1.00000	########
GOTERM_MF_FAT	GO:0008270~zinc ion binding	14	5.62249	0.45727	1.00E+00	APOBEC2, FGD2, BRD1	172	2311	12983	1.00000	1.00000	########
SP_PIR_KEYWORDS	zinc-finger	7	2.81124	0.31859	1.00E+00	FGD2, BRD1, TCF20, TI	246	1718	19235	1.00000	1.00000	########

Annotation Cluster 31	Enrichment Score: 0.6390877892113517											
Category	Term	Count	%	Fold Enrich	PValue	Genes	List Total	Pop Hits	Pop Total	Bonferroni	Benjamini	FDR
GOTERM_MF_FAT	GO:0022838~substrate specific channel activ	11	4.41767	2.08620	3.79E-02	KCNMB3, TTYH3, TRPV	172	398	12983	1.00000	0.92146	41.61014
GOTERM_MF_FAT	GO:0015267~channel activity	11	4.41767	2.01531	4.61E-02	KCNMB3, TTYH3, TRPV	172	412	12983	1.00000	0.90266	48.15240
GOTERM_MF_FAT	GO:0022803~passive transmembrane transp	11	4.41767	2.01043	4.67E-02	KCNMB3, TTYH3, TRPV	172	413	12983	1.00000	0.87748	48.62444
SP_PIR_KEYWORDS	ionic channel	8	3.21285	1.96707	1.13E-01	KCNMB3, TTYH3, TRPV	246	318	19235	1.00000	0.71319	80.49342
SP_PIR_KEYWORDS	transport	28	11.24498	1.31099	1.18E-01	RBP5, KCNMB3, AQP8,	246	1670	19235	1.00000	0.70971	82.03470
SP_PIR_KEYWORDS	ion transport	12	4.81928	1.62334	1.22E-01	KCNMB3, TTYH3, TRPV	246	578	19235	1.00000	0.70135	83.02044
GOTERM_MF_FAT	GO:0005216~ion channel activity	9	3.61446	1.75996	1.38E-01	KCNMB3, TTYH3, TRPV	172	386	12983	1.00000	0.99240	87.30087
SP_PIR_KEYWORDS	chloride channel	3	1.20482	4.18881	1.60E-01	TTYH3, CLCNKA, CLCN	246	56	19235	1.00000	0.79286	90.67494
GOTERM_CC_FAT	GO:0034707~chloride channel complex	3	1.20482	4.15000	1.61E-01	TTYH3, CLCNKA, CLCN	168	55	12782	1.00000	0.76223	89.06883
GOTERM_MF_FAT	GO:0005254~chloride channel activity	3	1.20482	3.14511	2.45E-01	TTYH3, CLCNKA, CLCN	172	72	12983	1.00000	0.99791	98.00282
SP_PIR_KEYWORDS	chloride	3	1.20482	3.12764	2.48E-01	TTYH3, CLCNKA, CLCN	246	75	19235	1.00000	0.85329	97.93842
GOTERM_MF_FAT	GO:0031404~chloride ion binding	3	1.20482	2.94088	2.69E-01	TTYH3, CLCNKA, CLCN	172	77	12983	1.00000	0.99797	98.73707
GOTERM_MF_FAT	GO:0005253~anion channel activity	3	1.20482	2.90318	2.74E-01	TTYH3, CLCNKA, CLCN	172	78	12983	1.00000	0.99760	98.84984
GOTERM_CC_FAT	GO:0034702~ion channel complex	5	2.00803	1.85569	2.83E-01	KCNMB3, TTYH3, KCTD	168	205	12782	1.00000	0.83253	98.47873
GOTERM_MF_FAT	GO:0005261~cation channel activity	6	2.40964	1.64689	2.96E-01	KCNMB3, TRPV1, P2RX	172	275	12983	1.00000	0.99693	99.25231
GOTERM_BP_FAT	GO:0006812~cation transport	10	4.01606	1.32232	3.39E-01	KCNMB3, TRPV1, SLC3	185	553	13528	1.00000	0.99643	99.87368
GOTERM_MF_FAT	GO:0043168~anion binding	3	1.20482	2.46139	3.42E-01	TTYH3, CLCNKA, CLCN	172	92	12983	1.00000	0.99828	99.70769
GOTERM_BP_FAT	GO:0006811~ion transport	13	5.22088	1.23778	3.52E-01	KCNMB3, SLC38A8, TR	185	768	13528	1.00000	0.99648	99.90904
GOTERM_MF_FAT	GO:0022836~gated channel activity	6	2.40964	1.46095	3.88E-01	KCNMB3, P2RX3, KCTD	172	310	12983	1.00000	0.99843	99.89171
GOTERM_MF_FAT	GO:0046873~metal ion transmembrane trans	6	2.40964	1.38078	4.35E-01	KCNMB3, TRPV1, P2RX	172	328	12983	1.00000	0.99912	99.96435
GOTERM_BP_FAT	GO:0015672~monovalent inorganic cation tra	6	2.40964	1.37970	4.35E-01	KCNMB3, SLC38A8, KC	185	318	13528	1.00000	0.99772	99.99004
GOTERM_MF_FAT	GO:0005244~voltage-gated ion channel activ	4	1.60643	1.54836	4.74E-01	KCTD1, CLCNKA, CLCN	172	195	12983	1.00000	0.99943	99.98692
GOTERM_MF_FAT	GO:0022832~voltage-gated channel activity	4	1.60643	1.54836	4.74E-01	KCTD1, CLCNKA, CLCN	172	195	12983	1.00000	0.99943	99.98692
SP_PIR_KEYWORDS	voltage-gated channel	3	1.20482	1.56382	5.72E-01	CLCNKA, CLCNKB, CAC	246	150	19235	1.00000	0.97396	99.99906
GOTERM_MF_FAT	GO:0008509~anion transmembrane transport	3	1.20482	1.54046	5.80E-01	TTYH3, CLCNKA, CLCN	172	147	12983	1.00000	0.99941	99.99943
GOTERM_BP_FAT	GO:0030001~metal ion transport	7	2.81124	1.10080	6.10E-01	KCNMB3, TRPV1, SLC3	185	465	13528	1.00000	0.99929	99.99997

Annotation Cluster 32	Enrichment Score: 0.6328372494847919											
Category	Term	Count	%	Fold Enrich	PValue	Genes	List Total	Pop Hits	Pop Total	Bonferroni	Benjamini	FDR
GOTERM_CC_FAT	GO:0044430~cytoskeletal part	19	7.63052	1.51847	7.20E-02	SHROOM1, MYL4, CRC	168	952	12782	1.00000	0.66351	60.93444
SP_PIR_KEYWORDS	cytoskeleton	13	5.22088	1.59824	1.14E-01	FGD2, SHROOM1, SEP	246	636	19235	1.00000	0.70752	80.93941
GOTERM_CC_FAT	GO:0005856~cytoskeleton	24	9.63855	1.32223	1.33E-01	FGD2, SHROOM1, MYL	168	1381	12782	1.00000	0.73354	83.36141
GOTERM_CC_FAT	GO:0015630~microtubule cytoskeleton	11	4.41767	1.52444	1.82E-01	SHROOM1, APP, KIF1A	168	549	12782	1.00000	0.75626	91.98152
GOTERM_CC_FAT	GO:0043228~non-membrane-bounded organ	29	11.64659	0.84993	8.95E-01	FGD2, MEAF6, SHROO	168	2596	12782	1.00000	0.99860	########
GOTERM_CC_FAT	GO:0043232~intracellular non-membrane-bou	29	11.64659	0.84993	8.95E-01	FGD2, MEAF6, SHROO	168	2596	12782	1.00000	0.99860	########

Annotation Cluster 33	Enrichment Score: 0.6327495045537525											
Category	Term	Count	%	Fold Enrich	PValue	Genes	List Total	Pop Hits	Pop Total	Bonferroni	Benjamini	FDR
GOTERM_BP_FAT	GO:0006915~apoptosis	12	4.81928	1.45763	1.98E-01	FGD2, LY86, LGALS7, D	185	602	13528	1.00000	0.99532	97.11794
GOTERM_BP_FAT	GO:0012501~programmed cell death	12	4.81928	1.43616	2.11E-01	FGD2, LY86, LGALS7, D	185	611	13528	1.00000	0.99559	97.78674
SP_PIR_KEYWORDS	Apoptosis	8	3.21285	1.64181	2.14E-01	TNS4, DNASE1, APP, T	246	381	19235	1.00000	0.82849	96.26038
GOTERM_BP_FAT	GO:0008219~cell death	13	5.22088	1.32214	2.74E-01	FGD2, LY86, LGALS7, D	185	719	13528	1.00000	0.99655	99.42177
GOTERM_BP_FAT	GO:0016265~death	13	5.22088	1.31301	2.81E-01	FGD2, LY86, LGALS7, D	185	724	13528	1.00000	0.99632	99.51383

Annotation Cluster 34	Enrichment Score: 0.6161177437785431											
Category	Term	Count	%	Fold Enrich	PValue	Genes	List Total	Pop Hits	Pop Total	Bonferroni	Benjamini	FDR
GOTERM_BP_FAT	GO:0006959~humoral immune response	4	1.60643	3.70250	9.27E-02	CCR6, FCN2, LY86, C1S	185	79	13528	1.00000	0.99821	79.15167
GOTERM_BP_FAT	GO:0045087~innate immune response	5	2.00803	2.64943	1.19E-01	TMEM173, SP100, FCN	185	138	13528	1.00000	0.99735	87.04522

SP_PIR_KEYWORDS	innate immunity	3	1.20482	3.00735	2.62E-01	TMEM173, FCN2, C1S	246	78	19235	1.00000	0.85023	98.40941
GOTERM_BP_FAT	GO:0006954~inflammatory response	7	2.81124	1.57499	2.78E-01	ORM1, TRPV1, FCN2, L	185	325	13528	1.00000	0.99632	99.47573
GOTERM_BP_FAT	GO:0006955~immune response	11	4.41767	1.16575	4.65E-01	TMEM173, CCR6, IL18E	185	690	13528	1.00000	0.99797	99.99578
SP_PIR_KEYWORDS	immune response	4	1.60643	1.39627	5.38E-01	TMEM173, FCN2, LY86,	246	224	19235	1.00000	0.96684	99.99735

Annotation Cluster 35 Enrichment Score: 0.6096154397284032

Category	Term	Count	%	Fold Enrich	PValue	Genes	List Total	Pop Hits	Pop Total	Bonferroni	Benjamini	FDR
GOTERM_BP_FAT	GO:0010033~response to organic substance	18	7.22892	1.82557	1.92E-02	SP100, THRA, IL1RN, C	185	721	13528	1.00000	0.97716	26.82695
GOTERM_BP_FAT	GO:0009755~hormone-mediated signaling	3	1.20482	4.21871	1.57E-01	THRA, PRKAR1B, CUZD	185	52	13528	1.00000	0.99770	93.67357
GOTERM_BP_FAT	GO:0009725~response to hormone stimulus	7	2.81124	1.39474	3.83E-01	THRA, PRKAR1B, IL1RN	185	367	13528	1.00000	0.99702	99.95775
GOTERM_BP_FAT	GO:0009719~response to endogenous stimul	7	2.81124	1.26388	4.74E-01	THRA, PRKAR1B, IL1RN	185	405	13528	1.00000	0.99801	99.99686
GOTERM_BP_FAT	GO:0032870~cellular response to hormone st	3	1.20482	1.64942	5.43E-01	THRA, PRKAR1B, CUZD	185	133	13528	1.00000	0.99892	99.99967
GOTERM_BP_FAT	GO:0048545~response to steroid hormone st	3	1.20482	1.14257	7.39E-01	IL1RN, SERPINA1, TFF1	185	192	13528	1.00000	0.99989	########

Annotation Cluster 36 Enrichment Score: 0.5697520389297343

Category	Term	Count	%	Fold Enrich	PValue	Genes	List Total	Pop Hits	Pop Total	Bonferroni	Benjamini	FDR
GOTERM_CC_FAT	GO:0016323~basolateral plasma membrane	6	2.40964	2.24877	1.27E-01	TNS4, LIMS2, ANK1, AF	168	203	12782	1.00000	0.73325	81.92283
GOTERM_CC_FAT	GO:0005925~focal adhesion	4	1.60643	2.98366	1.49E-01	TNS4, LIMS2, ARHGEF	168	102	12782	1.00000	0.76043	86.81111
GOTERM_CC_FAT	GO:0005924~cell-substrate adherens junction	4	1.60643	2.87107	1.61E-01	TNS4, LIMS2, ARHGEF	168	106	12782	1.00000	0.77569	89.04386
GOTERM_CC_FAT	GO:0030055~cell-substrate junction	4	1.60643	2.71726	1.81E-01	TNS4, LIMS2, ARHGEF	168	112	12782	1.00000	0.76553	91.81874
GOTERM_CC_FAT	GO:0005912~adherens junction	4	1.60643	1.96344	3.30E-01	TNS4, LIMS2, ARHGEF	168	155	12782	1.00000	0.85703	99.34987
GOTERM_CC_FAT	GO:0070161~anchoring junction	4	1.60643	1.76938	3.91E-01	TNS4, LIMS2, ARHGEF	168	172	12782	1.00000	0.88357	99.80308
SP_PIR_KEYWORDS	cell junction	6	2.40964	1.17581	5.77E-01	TNS4, CLDN18, LIMS2,	246	399	19235	1.00000	0.97292	99.99920
GOTERM_CC_FAT	GO:0030054~cell junction	7	2.81124	1.02815	6.75E-01	TNS4, CLDN18, LIMS2,	168	518	12782	1.00000	0.98213	99.99993

Annotation Cluster 37 Enrichment Score: 0.5640589805343672

Category	Term	Count	%	Fold Enrich	PValue	Genes	List Total	Pop Hits	Pop Total	Bonferroni	Benjamini	FDR
SP_PIR_KEYWORDS	receptor	31	12.44980	1.53122	1.85E-02	MCHR1, THRA, OR2T2,	246	1583	19235	0.99836	0.34783	22.45104
SP_PIR_KEYWORDS	transducer	19	7.63052	1.69981	3.11E-02	GNAT1, MCHR1, OR2A5	246	874	19235	0.99998	0.45281	34.97515
SP_PIR_KEYWORDS	g-protein coupled receptor	18	7.22892	1.72480	3.24E-02	MCHR1, OR2A5, OR2T2	246	816	19235	0.99999	0.43283	36.22259
GOTERM_BP_FAT	GO:0050890~cognition	17	6.82731	1.36756	1.74E-01	GNAT1, SLC45A2, OR2	185	909	13528	1.00000	0.99725	95.43323
INTERPRO	IPR017452:GPCR, rhodopsin-like superfamily	14	5.62249	1.42777	1.81E-01	MCHR1, OR2A5, OR2T2	225	726	16659	1.00000	0.95979	94.24649
INTERPRO	IPR000276:7TM GPCR, rhodopsin-like	14	5.62249	1.42580	1.82E-01	MCHR1, OR2A5, OR2T2	225	727	16659	1.00000	0.95646	94.37199
GOTERM_BP_FAT	GO:0007186~G-protein coupled receptor prot	20	8.03213	1.30230	1.90E-01	GNAT1, MCHR1, OR2A5	185	1123	13528	1.00000	0.99718	96.65087
SP_PIR_KEYWORDS	cell membrane	33	13.25301	1.17607	2.32E-01	MCHR1, CLDN18, LIMS	246	2194	19235	1.00000	0.84367	97.28413
GOTERM_BP_FAT	GO:0007600~sensory perception	14	5.62249	1.26388	3.09E-01	GNAT1, SLC45A2, OR2	185	810	13528	1.00000	0.99662	99.74091
GOTERM_BP_FAT	GO:0050877~neurological system process	19	7.63052	1.14823	3.81E-01	GNAT1, KCNMB3, SLC4	185	1210	13528	1.00000	0.99728	99.95597
PIR_SUPERFAMILY	PIRSF800006:rhodopsin-like G protein-couple	13	5.22088	1.19793	3.90E-01	MCHR1, OR2A5, OR2T2	117	686	7396	1.00000	1.00000	99.67970
GOTERM_BP_FAT	GO:0007606~sensory perception of chemical	8	3.21285	1.22384	4.76E-01	GNAT1, OR2A5, OR2T2	185	478	13528	1.00000	0.99795	99.99697
SP_PIR_KEYWORDS	sensory transduction	9	3.61446	1.18272	4.88E-01	GNAT1, SLC45A2, OR2	246	595	19235	1.00000	0.95549	99.98919
KEGG_PATHWAY	hsa04740:Olfactory transduction	6	2.40964	1.23848	5.25E-01	OR2A5, OR2T2, OR6B3	65	379	5085	1.00000	0.99975	99.96162
GOTERM_BP_FAT	GO:0007166~cell surface receptor linked sigr	26	10.44177	1.02437	5.54E-01	MCHR1, OR2T2, TRPV1	185	1856	13528	1.00000	0.99899	99.99978
SP_PIR_KEYWORDS	olfaction	5	2.00803	0.95588	7.68E-01	OR2A5, OR2T2, OR6B3	246	409	19235	1.00000	0.99524	########
PIR_SUPERFAMILY	PIRSF003152:G protein-coupled olfactory rec	5	2.00803	0.89538	8.11E-01	OR2A5, OR2T2, OR6B3	117	353	7396	1.00000	1.00000	########
GOTERM_MF_FAT	GO:0004984~olfactory receptor activity	5	2.00803	0.87567	8.24E-01	OR2A5, OR2T2, OR6B3	172	431	12983	1.00000	0.99985	########
INTERPRO	IPR000725:Olfactory receptor	5	2.00803	0.86495	8.31E-01	OR2A5, OR2T2, OR6B3	225	428	16659	1.00000	1.00000	########
GOTERM_BP_FAT	GO:0007608~sensory perception of smell	5	2.00803	0.84831	8.42E-01	OR2A5, OR2T2, OR6B3	185	431	13528	1.00000	0.99999	########

Annotation Cluster 38 Enrichment Score: 0.5465034385059866

Category	Term	Count	%	Fold Enrich	PValue	Genes	List Total	Pop Hits	Pop Total	Bonferroni	Benjamini	FDR
GOTERM_CC_FAT	GO:0005929~cilium	6	2.40964	3.53876	2.70E-02	GNAT1, MCHR1, APP, (	168	129	12782	0.99627	0.55010	29.15228
GOTERM_BP_FAT	GO:0009416~response to light stimulus	3	1.20482	1.58966	5.63E-01	GNAT1, APP, RHO	185	138	13528	1.00000	0.99910	99.99984
GOTERM_BP_FAT	GO:0009628~response to abiotic stimulus	6	2.40964	1.19224	5.64E-01	GNAT1, APP, TRPV1, P	185	368	13528	1.00000	0.99908	99.99985
GOTERM_BP_FAT	GO:0009314~response to radiation	3	1.20482	1.09686	7.59E-01	GNAT1, APP, RHO	185	200	13528	1.00000	0.99993	########

Annotation Cluster 39 Enrichment Score: 0.5253630073748161

Category	Term	Count	%	Fold Enrich	PValue	Genes	List Total	Pop Hits	Pop Total	Bonferroni	Benjamini	FDR

GOTERM_BP_FAT	GO:0006164~purine nucleotide biosynthetic p	5	2.00803	2.47042	1.43E-01	ATP9A, ATP6V1B1, ATF	185	148	13528	1.00000	0.99842	91.68253
GOTERM_MF_FAT	GO:0042626~ATPase activity, coupled to trar	4	1.60643	2.77000	1.74E-01	ATP9A, ATP6V1B1, ATF	172	109	12983	1.00000	0.99540	92.97440
GOTERM_MF_FAT	GO:0043492~ATPase activity, coupled to mov	4	1.60643	2.74482	1.77E-01	ATP9A, ATP6V1B1, ATF	172	110	12983	1.00000	0.99407	93.35063
GOTERM_MF_FAT	GO:0016820~hydrolase activity, acting on aci	4	1.60643	2.72009	1.80E-01	ATP9A, ATP6V1B1, ATF	172	111	12983	1.00000	0.99259	93.70990
GOTERM_BP_FAT	GO:0009152~purine ribonucleotide biosynthe	4	1.60643	2.49998	2.13E-01	ATP9A, ATP6V1B1, ATF	185	117	13528	1.00000	0.99498	97.90239
GOTERM_MF_FAT	GO:0015399~primary active transmembrane	4	1.60643	2.47484	2.17E-01	ATP9A, ATP6V1B1, ATF	172	122	12983	1.00000	0.99662	96.69568
GOTERM_MF_FAT	GO:0015405~P-P-bond-hydrolysis-driven trar	4	1.60643	2.47484	2.17E-01	ATP9A, ATP6V1B1, ATF	172	122	12983	1.00000	0.99662	96.69568
GOTERM_CC_FAT	GO:0005773~vacuole	6	2.40964	1.81151	2.34E-01	SPACA3, PLA2G4F, ATI	168	252	12782	1.00000	0.79776	96.49053
GOTERM_BP_FAT	GO:0009260~ribonucleotide biosynthetic proc	4	1.60643	2.35885	2.38E-01	ATP9A, ATP6V1B1, ATF	185	124	13528	1.00000	0.99625	98.75493
GOTERM_BP_FAT	GO:0006163~purine nucleotide metabolic pro	5	2.00803	1.96571	2.47E-01	ATP9A, ATP6V1B1, ATF	185	186	13528	1.00000	0.99569	98.97084
GOTERM_BP_FAT	GO:0009165~nucleotide biosynthetic process	5	2.00803	1.96571	2.47E-01	ATP9A, ATP6V1B1, ATF	185	186	13528	1.00000	0.99569	98.97084
GOTERM_MF_FAT	GO:0042625~ATPase activity, coupled to trar	3	1.20482	3.06010	2.55E-01	ATP9A, ATP6V1B1, ATF	172	74	12983	1.00000	0.99779	98.33417
GOTERM_BP_FAT	GO:0034404~nucleobase, nucleoside and nu	5	2.00803	1.89441	2.68E-01	ATP9A, ATP6V1B1, ATF	185	193	13528	1.00000	0.99638	99.34870
GOTERM_BP_FAT	GO:0034654~nucleobase, nucleoside, nucleo	5	2.00803	1.89441	2.68E-01	ATP9A, ATP6V1B1, ATF	185	193	13528	1.00000	0.99638	99.34870
GOTERM_BP_FAT	GO:0044271~nitrogen compound biosynthetic	7	2.81124	1.57499	2.78E-01	ATP9A, ALDH4A1, SUL	185	325	13528	1.00000	0.99632	99.47573
GOTERM_BP_FAT	GO:0009150~purine ribonucleotide metabolic	4	1.60643	2.11955	2.90E-01	ATP9A, ATP6V1B1, ATF	185	138	13528	1.00000	0.99643	99.59599
GOTERM_BP_FAT	GO:0009259~ribonucleotide metabolic proces	4	1.60643	1.98978	3.23E-01	ATP9A, ATP6V1B1, ATF	185	147	13528	1.00000	0.99644	99.81447
GOTERM_BP_FAT	GO:0006754~ATP biosynthetic process	3	1.20482	2.46486	3.42E-01	ATP9A, ATP6V1B1, ATF	185	89	13528	1.00000	0.99640	99.88214
GOTERM_BP_FAT	GO:0009206~purine ribonucleoside triphosph	3	1.20482	2.23850	3.86E-01	ATP9A, ATP6V1B1, ATF	185	98	13528	1.00000	0.99704	99.96145
GOTERM_BP_FAT	GO:0009145~purine nucleoside triphosphate	3	1.20482	2.21589	3.91E-01	ATP9A, ATP6V1B1, ATF	185	99	13528	1.00000	0.99697	99.96605
GOTERM_BP_FAT	GO:0009201~ribonucleoside triphosphate bio	3	1.20482	2.21589	3.91E-01	ATP9A, ATP6V1B1, ATF	185	99	13528	1.00000	0.99697	99.96605
GOTERM_BP_FAT	GO:0009142~nucleoside triphosphate biosynt	3	1.20482	2.15072	4.05E-01	ATP9A, ATP6V1B1, ATF	185	102	13528	1.00000	0.99757	99.97689
GOTERM_BP_FAT	GO:0046034~ATP metabolic process	3	1.20482	2.08927	4.19E-01	ATP9A, ATP6V1B1, ATF	185	105	13528	1.00000	0.99708	99.98435
GOTERM_MF_FAT	GO:0016887~ATPase activity	6	2.40964	1.35597	4.50E-01	VCP, ATP9A, CETN2, A	172	334	12983	1.00000	0.99922	99.97580
GOTERM_BP_FAT	GO:0009205~purine ribonucleoside triphosph	3	1.20482	1.87498	4.74E-01	ATP9A, ATP6V1B1, ATF	185	117	13528	1.00000	0.99811	99.99685
GOTERM_BP_FAT	GO:0009199~ribonucleoside triphosphate me	3	1.20482	1.85909	4.79E-01	ATP9A, ATP6V1B1, ATF	185	118	13528	1.00000	0.99786	99.99725
GOTERM_MF_FAT	GO:0042623~ATPase activity, coupled	5	2.00803	1.38755	4.83E-01	ATP9A, CETN2, ATP6V1	172	272	12983	1.00000	0.99928	99.98979
GOTERM_BP_FAT	GO:0009144~purine nucleoside triphosphate	3	1.20482	1.79814	4.96E-01	ATP9A, ATP6V1B1, ATF	185	122	13528	1.00000	0.99801	99.99842
GOTERM_BP_FAT	GO:0009141~nucleoside triphosphate metabo	3	1.20482	1.67460	5.34E-01	ATP9A, ATP6V1B1, ATF	185	131	13528	1.00000	0.99880	99.99955

Annotation Cluster 40 Enrichment Score: 0.5151783203813677

Category	Term	Count	%	Fold Enrich	PValue	Genes	List Total	Pop Hits	Pop Total	Bonferroni	Benjamini	FDR
GOTERM_BP_FAT	GO:0030036~actin cytoskeleton organization	6	2.40964	1.94135	1.95E-01	FGD2, TNNT2, SHROOI	185	226	13528	1.00000	0.99596	96.95668
GOTERM_BP_FAT	GO:0030029~actin filament-based process	6	2.40964	1.82052	2.32E-01	FGD2, TNNT2, SHROOI	185	241	13528	1.00000	0.99632	98.56871
GOTERM_BP_FAT	GO:0007010~cytoskeleton organization	9	3.61446	1.50945	2.43E-01	FGD2, TNNT2, SHROOI	185	436	13528	1.00000	0.99600	98.87431
GOTERM_BP_FAT	GO:0032989~cellular component morphogen	5	2.00803	0.92096	7.93E-01	TNNT2, SHROOM1, API	185	397	13528	1.00000	0.99995	########

Annotation Cluster 41 Enrichment Score: 0.5043738741571876

Category	Term	Count	%	Fold Enrich	PValue	Genes	List Total	Pop Hits	Pop Total	Bonferroni	Benjamini	FDR
GOTERM_BP_FAT	GO:0001503~ossification	4	1.60643	2.54345	2.06E-01	SOST, THRA, ATP6V1B	185	115	13528	1.00000	0.99549	97.57807
GOTERM_BP_FAT	GO:0060348~bone development	4	1.60643	2.37803	2.35E-01	SOST, THRA, ATP6V1B	185	123	13528	1.00000	0.99625	98.65629
GOTERM_BP_FAT	GO:0001501~skeletal system development	5	2.00803	1.14615	6.34E-01	SOST, THRA, COL12A1	185	319	13528	1.00000	0.99950	99.99999

Annotation Cluster 42 Enrichment Score: 0.5024081725428344

Category	Term	Count	%	Fold Enrich	PValue	Genes	List Total	Pop Hits	Pop Total	Bonferroni	Benjamini	FDR
GOTERM_BP_FAT	GO:0009408~response to heat	3	1.20482	3.91737	1.77E-01	TRPV1, P2RX3, HSPB3	185	56	13528	1.00000	0.99661	95.64562
GOTERM_BP_FAT	GO:0009266~response to temperature stimul	3	1.20482	2.64305	3.12E-01	TRPV1, P2RX3, HSPB3	185	83	13528	1.00000	0.99634	99.75862
GOTERM_BP_FAT	GO:0009628~response to abiotic stimulus	6	2.40964	1.19224	5.64E-01	GNAT1, APP, TRPV1, P	185	368	13528	1.00000	0.99908	99.99985

Annotation Cluster 43 Enrichment Score: 0.4871029828279582

Category	Term	Count	%	Fold Enrich	PValue	Genes	List Total	Pop Hits	Pop Total	Bonferroni	Benjamini	FDR
GOTERM_BP_FAT	GO:0051090~regulation of transcription factor	4	1.60643	2.83978	1.65E-01	SP100, THRA, RIPK1, T	185	103	13528	1.00000	0.99761	94.54783
GOTERM_BP_FAT	GO:0051091~positive regulation of transcripti	3	1.20482	3.65622	1.96E-01	SP100, RIPK1, TSSK4	185	60	13528	1.00000	0.99567	97.04989
GOTERM_BP_FAT	GO:0051101~regulation of DNA binding	4	1.60643	2.41733	2.27E-01	SP100, THRA, RIPK1, T	185	121	13528	1.00000	0.99627	98.43762
GOTERM_BP_FAT	GO:0043388~positive regulation of DNA bindi	3	1.20482	3.13390	2.46E-01	SP100, RIPK1, TSSK4	185	70	13528	1.00000	0.99598	98.95367
GOTERM_BP_FAT	GO:0051099~positive regulation of binding	3	1.20482	2.81247	2.87E-01	SP100, RIPK1, TSSK4	185	78	13528	1.00000	0.99648	99.56941
GOTERM_BP_FAT	GO:0051098~regulation of binding	4	1.60643	1.91175	3.42E-01	SP100, THRA, RIPK1, T	185	153	13528	1.00000	0.99620	99.88331

GOTERM_BP_FAT	GO:0044093~positive regulation of molecular	7	2.81124	0.87350	8.14E-01	TNNT2, MYL4, SP100, V	185	586	13528	1.00000	0.99997	########
SP_PIR_KEYWORDS	ubl conjugation	6	2.40964	0.79787	8.73E-01	SP100, THRA, RIPK1, K	246	588	19235	1.00000	0.99891	########

Annotation Cluster 44 Enrichment Score: 0.4746520824760008

Category	Term	Count	%	Fold Enrich	PValue	Genes	List Total	Pop Hits	Pop Total	Bonferroni	Benjamini	FDR
SP_PIR_KEYWORDS	ionic channel	8	3.21285	1.96707	1.13E-01	KCNMB3, TTYH3, TRPV	246	318	19235	1.00000	0.71319	80.49342
SP_PIR_KEYWORDS	calcium channel	3	1.20482	3.84546	1.82E-01	TRPV1, TRPV5, CACNA	246	61	19235	1.00000	0.81523	93.57851
GOTERM_MF_FAT	GO:0005262~calcium channel activity	3	1.20482	2.86643	2.79E-01	TRPV1, TRPV5, CACNA	172	79	12983	1.00000	0.99720	98.95317
SP_PIR_KEYWORDS	calcium transport	3	1.20482	2.75968	2.95E-01	TRPV1, TRPV5, CACNA	246	85	19235	1.00000	0.87408	99.14849
GOTERM_MF_FAT	GO:0005261~cation channel activity	6	2.40964	1.64689	2.96E-01	KCNMB3, TRPV1, P2RX	172	275	12983	1.00000	0.99693	99.25231
GOTERM_MF_FAT	GO:0046873~metal ion transmembrane trans	6	2.40964	1.38078	4.35E-01	KCNMB3, TRPV1, P2RX	172	328	12983	1.00000	0.99912	99.96435
GOTERM_BP_FAT	GO:0006816~calcium ion transport	3	1.20482	1.54488	5.78E-01	TRPV1, TRPV5, CACNA	185	142	13528	1.00000	0.99914	99.99991
GOTERM_BP_FAT	GO:0030001~metal ion transport	7	2.81124	1.10080	6.10E-01	KCNMB3, TRPV1, SLC3	185	465	13528	1.00000	0.99929	99.99997
GOTERM_BP_FAT	GO:0015674~di-, tri-valent inorganic cation tr	3	1.20482	1.24644	6.94E-01	TRPV1, TRPV5, CACNA	185	176	13528	1.00000	0.99981	########

Annotation Cluster 45 Enrichment Score: 0.47267458116333533

Category	Term	Count	%	Fold Enrich	PValue	Genes	List Total	Pop Hits	Pop Total	Bonferroni	Benjamini	FDR
GOTERM_BP_FAT	GO:0006091~generation of precursor metabo	8	3.21285	1.86899	1.33E-01	MCHR1, GCK, LDHAL6E	185	313	13528	1.00000	0.99789	89.88712
GOTERM_BP_FAT	GO:0042592~homeostatic process	15	6.02410	1.46054	1.44E-01	KCNMB3, MCHR1, TRP	185	751	13528	1.00000	0.99811	91.83186
GOTERM_BP_FAT	GO:0048878~chemical homeostasis	11	4.41767	1.57103	1.60E-01	KCNMB3, MCHR1, ASG	185	512	13528	1.00000	0.99757	94.03106
GOTERM_BP_FAT	GO:0055080~cation homeostasis	7	2.81124	1.78976	1.94E-01	MCHR1, APP, CCR6, PY	185	286	13528	1.00000	0.99634	96.91251
GOTERM_BP_FAT	GO:0050801~ion homeostasis	8	3.21285	1.43030	3.22E-01	KCNMB3, MCHR1, APP	185	409	13528	1.00000	0.99662	99.81070
GOTERM_BP_FAT	GO:0030005~cellular di-, tri-valent inorganic c	5	2.00803	1.61067	3.73E-01	MCHR1, APP, CCR6, PY	185	227	13528	1.00000	0.99719	99.94619
GOTERM_BP_FAT	GO:0055066~di-, tri-valent inorganic cation ho	5	2.00803	1.52980	4.10E-01	MCHR1, APP, CCR6, PY	185	239	13528	1.00000	0.99763	99.97951
GOTERM_BP_FAT	GO:0055082~cellular chemical homeostasis	7	2.81124	1.34703	4.14E-01	KCNMB3, MCHR1, APP	185	380	13528	1.00000	0.99742	99.98191
GOTERM_BP_FAT	GO:0019725~cellular homeostasis	8	3.21285	1.25535	4.49E-01	KCNMB3, MCHR1, APP	185	466	13528	1.00000	0.99803	99.99329
GOTERM_BP_FAT	GO:0006874~cellular calcium ion homeostasi	4	1.60643	1.59835	4.50E-01	MCHR1, APP, CCR6, PY	185	183	13528	1.00000	0.99796	99.99352
GOTERM_BP_FAT	GO:0030003~cellular cation homeostasis	5	2.00803	1.43946	4.56E-01	MCHR1, APP, CCR6, PY	185	254	13528	1.00000	0.99785	99.99455
GOTERM_BP_FAT	GO:0055074~calcium ion homeostasis	4	1.60643	1.55584	4.74E-01	MCHR1, APP, CCR6, PY	185	188	13528	1.00000	0.99820	99.99682
GOTERM_BP_FAT	GO:0006875~cellular metal ion homeostasis	4	1.60643	1.49233	5.00E-01	MCHR1, APP, CCR6, PY	185	196	13528	1.00000	0.99804	99.99858
GOTERM_BP_FAT	GO:0055065~metal ion homeostasis	4	1.60643	1.42682	5.30E-01	MCHR1, APP, CCR6, PY	185	205	13528	1.00000	0.99876	99.99948
GOTERM_BP_FAT	GO:0006873~cellular ion homeostasis	6	2.40964	1.17312	5.79E-01	KCNMB3, MCHR1, APP	185	374	13528	1.00000	0.99906	99.99991

Annotation Cluster 46 Enrichment Score: 0.4691801618635848

Category	Term	Count	%	Fold Enrich	PValue	Genes	List Total	Pop Hits	Pop Total	Bonferroni	Benjamini	FDR
SP_PIR_KEYWORDS	vision	4	1.60643	2.25010	2.61E-01	GNAT1, SLC45A2, CAC	246	139	19235	1.00000	0.85419	98.38080
GOTERM_BP_FAT	GO:0007600~sensory perception	14	5.62249	1.26388	3.09E-01	GNAT1, SLC45A2, OR2	185	810	13528	1.00000	0.99662	99.74091
GOTERM_BP_FAT	GO:0050953~sensory perception of light stim	5	2.00803	1.69269	3.39E-01	CRYBB1, GNAT1, SLC4	185	216	13528	1.00000	0.99662	99.87215
GOTERM_BP_FAT	GO:0007601~visual perception	5	2.00803	1.69269	3.39E-01	CRYBB1, GNAT1, SLC4	185	216	13528	1.00000	0.99662	99.87215
SP_PIR_KEYWORDS	sensory transduction	9	3.61446	1.18272	4.88E-01	GNAT1, SLC45A2, OR2	246	595	19235	1.00000	0.95549	99.98919

Annotation Cluster 47 Enrichment Score: 0.4492954830695583

Category	Term	Count	%	Fold Enrich	PValue	Genes	List Total	Pop Hits	Pop Total	Bonferroni	Benjamini	FDR
GOTERM_BP_FAT	GO:0055002~striated muscle cell developme	3	1.20482	4.21871	1.57E-01	TNNT2, APP, CAPN3	185	52	13528	1.00000	0.99770	93.67357
GOTERM_BP_FAT	GO:0055001~muscle cell development	3	1.20482	3.91737	1.77E-01	TNNT2, APP, CAPN3	185	56	13528	1.00000	0.99661	95.64562
GOTERM_BP_FAT	GO:0051146~striated muscle cell differentiati	3	1.20482	2.49287	3.37E-01	TNNT2, APP, CAPN3	185	88	13528	1.00000	0.99673	99.86697
GOTERM_BP_FAT	GO:0042692~muscle cell differentiation	3	1.20482	1.81300	4.92E-01	TNNT2, APP, CAPN3	185	121	13528	1.00000	0.99805	99.99818
GOTERM_BP_FAT	GO:0007517~muscle organ development	4	1.60643	1.38624	5.51E-01	TNNT2, APP, MYL4, CA	185	211	13528	1.00000	0.99898	99.99975
GOTERM_BP_FAT	GO:0032989~cellular component morphogen	5	2.00803	0.92096	7.93E-01	TNNT2, SHROOM1, API	185	397	13528	1.00000	0.99995	########

Annotation Cluster 48 Enrichment Score: 0.4485736356518668

Category	Term	Count	%	Fold Enrich	PValue	Genes	List Total	Pop Hits	Pop Total	Bonferroni	Benjamini	FDR
INTERPRO	IPR001965:Zinc finger, PHD-type	3	1.20482	2.46800	3.42E-01	BRD1, TCF20, SP100	225	90	16659	1.00000	0.99443	99.75067
INTERPRO	IPR019786:Zinc finger, PHD-type, conserved	3	1.20482	2.38839	3.56E-01	BRD1, TCF20, SP100	225	93	16659	1.00000	0.99517	99.81939
SMART	SM00249:PHD	3	1.20482	2.31018	3.70E-01	BRD1, TCF20, SP100	131	90	9079	1.00000	0.98858	99.48991

Annotation Cluster 49 Enrichment Score: 0.4482117438212104

Category	Term	Count	%	Fold Enrich	PValue	Genes	List Total	Pop Hits	Pop Total	Bonferroni	Benjamini	FDR
INTERPRO	IPR001849:Pleckstrin homology	7	2.81124	1.87105	1.71E-01	FGD2, DAB2IP, KIF1A, /	225	277	16659	1.00000	0.95584	93.14169
SMART	SM00233:PH	7	2.81124	1.75140	2.06E-01	FGD2, DAB2IP, KIF1A, /	131	277	9079	1.00000	0.94878	92.79476
UP_SEQ_FEATURE	domain:DH	3	1.20482	3.47889	2.12E-01	FGD2, ARHGEF7, PLEK	246	67	19113	1.00000	0.99635	97.49379
INTERPRO	IPR000219:Dbl homology (DH) domain	3	1.20482	3.21913	2.37E-01	FGD2, ARHGEF7, PLEK	225	69	16659	1.00000	0.97645	97.94698
GOTERM_MF_FAT	GO:0005089~Rho guanyl-nucleotide exchang	3	1.20482	3.06010	2.55E-01	FGD2, ARHGEF7, PLEK	172	74	12983	1.00000	0.99779	98.33417
GOTERM_BP_FAT	GO:0051056~regulation of small GTPase me	6	2.40964	1.74106	2.59E-01	FGD2, DAB2IP, ARHGE	185	252	13528	1.00000	0.99585	99.20559
SMART	SM00325:RhoGEF	3	1.20482	3.01328	2.60E-01	FGD2, ARHGEF7, PLEK	131	69	9079	1.00000	0.95807	96.76570
GOTERM_BP_FAT	GO:0051336~regulation of hydrolase activity	7	2.81124	1.51890	3.11E-01	GNAT1, FGD2, TNNT2,	185	337	13528	1.00000	0.99649	99.75098
GOTERM_BP_FAT	GO:0046578~regulation of Ras protein signal	5	2.00803	1.74106	3.17E-01	FGD2, ARHGEF7, ACAF	185	210	13528	1.00000	0.99643	99.78302
GOTERM_MF_FAT	GO:0005088~Ras guanyl-nucleotide exchang	3	1.20482	2.57327	3.23E-01	FGD2, ARHGEF7, PLEK	172	88	12983	1.00000	0.99790	99.56289
SP_PIR_KEYWORDS	GTPase activation	4	1.60643	1.96707	3.29E-01	DAB2IP, ACAP1, PLEKH	246	159	19235	1.00000	0.88709	99.57009
UP_SEQ_FEATURE	domain:PH	5	2.00803	1.63914	3.61E-01	DAB2IP, KIF1A, ARHGE	246	237	19113	1.00000	0.99969	99.90174
GOTERM_BP_FAT	GO:0035023~regulation of Rho protein signal	3	1.20482	2.21589	3.91E-01	FGD2, ARHGEF7, PLEK	185	99	13528	1.00000	0.99697	99.96605
GOTERM_BP_FAT	GO:0032318~regulation of Ras GTPase activ	3	1.20482	2.10936	4.15E-01	FGD2, ACAP1, TBC1D2	185	104	13528	1.00000	0.99729	99.98217
GOTERM_MF_FAT	GO:0005083~small GTPase regulator activity	5	2.00803	1.37742	4.89E-01	FGD2, ARHGEF7, ACAF	172	274	12983	1.00000	0.99907	99.99125
GOTERM_BP_FAT	GO:0043087~regulation of GTPase activity	3	1.20482	1.78352	5.01E-01	FGD2, ACAP1, TBC1D2	185	123	13528	1.00000	0.99798	99.99862
GOTERM_MF_FAT	GO:0005096~GTPase activator activity	4	1.60643	1.37241	5.57E-01	DAB2IP, ACAP1, PLEKH	172	220	12983	1.00000	0.99933	99.99880
GOTERM_MF_FAT	GO:0005085~guanyl-nucleotide exchange fac	3	1.20482	1.48979	5.98E-01	FGD2, ARHGEF7, PLEK	172	152	12983	1.00000	0.99945	99.99969
GOTERM_MF_FAT	GO:0030695~GTPase regulator activity	6	2.40964	1.12103	6.19E-01	FGD2, DAB2IP, ARHGE	172	404	12983	1.00000	0.99934	99.99985
GOTERM_MF_FAT	GO:0060589~nucleoside-triphosphatase regu	6	2.40964	1.09660	6.38E-01	FGD2, DAB2IP, ARHGE	172	413	12983	1.00000	0.99923	99.99993
GOTERM_MF_FAT	GO:0008047~enzyme activator activity	5	2.00803	1.12661	6.48E-01	APP, DAB2IP, ACAP1, F	172	335	12983	1.00000	0.99927	99.99995

Annotation Cluster 50 Enrichment Score: 0.40738304574015616

Category	Term	Count	%	Fold Enrich	PValue	Genes	List Total	Pop Hits	Pop Total	Bonferroni	Benjamini	FDR
SP_PIR_KEYWORDS	Serine protease inhibitor	3	1.20482	2.93216	2.71E-01	APP, ITIH1, SERPINA1	246	80	19235	1.00000	0.85189	98.66580
GOTERM_MF_FAT	GO:0004857~enzyme inhibitor activity	6	2.40964	1.67739	2.84E-01	HSPBP1, APP, PPP1R1	172	270	12983	1.00000	0.99675	99.03954
GOTERM_MF_FAT	GO:0004867~serine-type endopeptidase inhil	3	1.20482	2.46139	3.42E-01	APP, ITIH1, SERPINA1	172	92	12983	1.00000	0.99828	99.70769
SP_PIR_KEYWORDS	protease inhibitor	3	1.20482	2.19227	3.96E-01	APP, ITIH1, SERPINA1	246	107	19235	1.00000	0.92516	99.89798
GOTERM_MF_FAT	GO:0004866~endopeptidase inhibitor activity	3	1.20482	1.56171	5.72E-01	APP, ITIH1, SERPINA1	172	145	12983	1.00000	0.99942	99.99926
GOTERM_MF_FAT	GO:0030414~peptidase inhibitor activity	3	1.20482	1.48005	6.01E-01	APP, ITIH1, SERPINA1	172	153	12983	1.00000	0.99940	99.99973

Annotation Cluster 51 Enrichment Score: 0.40202590742904837

Category	Term	Count	%	Fold Enrich	PValue	Genes	List Total	Pop Hits	Pop Total	Bonferroni	Benjamini	FDR
GOTERM_CC_FAT	GO:0005773~vacuole	6	2.40964	1.81151	2.34E-01	SPACA3, PLA2G4F, ATI	168	252	12782	1.00000	0.79776	96.49053
GOTERM_CC_FAT	GO:0000323~lytic vacuole	4	1.60643	1.44234	5.16E-01	SPACA3, PLA2G4F, SL(	168	211	12782	1.00000	0.93542	99.98905
GOTERM_CC_FAT	GO:0005764~lysosome	4	1.60643	1.44234	5.16E-01	SPACA3, PLA2G4F, SL(	168	211	12782	1.00000	0.93542	99.98905

Annotation Cluster 52 Enrichment Score: 0.394606308834446

Category	Term	Count	%	Fold Enrich	PValue	Genes	List Total	Pop Hits	Pop Total	Bonferroni	Benjamini	FDR
SP_PIR_KEYWORDS	heme	4	1.60643	2.58483	2.00E-01	CYP2A13, CYB561, MB,	246	121	19235	1.00000	0.82601	95.27047
SP_PIR_KEYWORDS	iron	6	2.40964	1.64613	2.98E-01	CYP2A13, APP, ALOX1:	246	285	19235	1.00000	0.86889	99.20465
UP_SEQ_FEATURE	metal ion-binding site:Iron (heme axial ligand)	3	1.20482	2.37842	3.58E-01	CYP2A13, CYB561, CYI	246	98	19113	1.00000	0.99972	99.89557
GOTERM_MF_FAT	GO:0005506~iron ion binding	6	2.40964	1.47044	3.82E-01	CYP2A13, APP, ALOX1:	172	308	12983	1.00000	0.99859	99.87807
GOTERM_MF_FAT	GO:0020037~heme binding	3	1.20482	1.87147	4.75E-01	CYP2A13, MB, CYP4B1	172	121	12983	1.00000	0.99931	99.98736
GOTERM_MF_FAT	GO:0046906~tetrapyrrole binding	3	1.20482	1.75541	5.09E-01	CYP2A13, MB, CYP4B1	172	129	12983	1.00000	0.99926	99.99500
GOTERM_BP_FAT	GO:0055114~oxidation reduction	7	2.81124	0.80105	8.72E-01	CYP2A13, LDHAL6B, AI	185	639	13528	1.00000	0.99999	########

Annotation Cluster 53 Enrichment Score: 0.3797669152173389

Category	Term	Count	%	Fold Enrich	PValue	Genes	List Total	Pop Hits	Pop Total	Bonferroni	Benjamini	FDR
UP_SEQ_FEATURE	domain:SH2	3	1.20482	2.42797	3.49E-01	TNS4, FGR, GRAPL	246	96	19113	1.00000	0.99969	99.86960
SP_PIR_KEYWORDS	SH2 domain	3	1.20482	2.11327	4.14E-01	TNS4, FGR, GRAPL	246	111	19235	1.00000	0.93051	99.93219
INTERPRO	IPR000980:SH2 motif	3	1.20482	2.00108	4.41E-01	TNS4, FGR, GRAPL	225	111	16659	1.00000	0.99809	99.97625
SMART	SM00252:SH2	3	1.20482	1.87312	4.74E-01	TNS4, FGR, GRAPL	131	111	9079	1.00000	0.99677	99.93466

Annotation Cluster 54 Enrichment Score: 0.37820747201209426

Category	Term	Count	%	Fold Enrich	PValue	Genes	List Total	Pop Hits	Pop Total	Bonferroni	Benjamini	FDR

GOTERM_BP_FAT	GO:0051605~protein maturation by peptide b	3	1.20482	2.55085	3.27E-01	FCN2, CUZD1, C1S	185	86	13528	1.00000	0.99624	99.83084
GOTERM_BP_FAT	GO:0016485~protein processing	3	1.20482	1.95869	4.52E-01	FCN2, CUZD1, C1S	185	112	13528	1.00000	0.99791	99.99381
GOTERM_BP_FAT	GO:0051604~protein maturation	3	1.20482	1.79814	4.96E-01	FCN2, CUZD1, C1S	185	122	13528	1.00000	0.99801	99.99842

Annotation Cluster 55 Enrichment Score: 0.37301980450997557

Category	Term	Count	%	Fold Enrich	PValue	Genes	List Total	Pop Hits	Pop Total	Bonferroni	Benjamini	FDR
GOTERM_CC_FAT	GO:0030017~sarcomere	3	1.20482	2.32908	3.67E-01	TNNT2, MYL4, ANK1	168	98	12782	1.00000	0.88596	99.68327
GOTERM_CC_FAT	GO:0030016~myofibril	3	1.20482	2.05631	4.27E-01	TNNT2, MYL4, ANK1	168	111	12782	1.00000	0.90624	99.90912
GOTERM_CC_FAT	GO:0044449~contractile fiber part	3	1.20482	2.01991	4.36E-01	TNNT2, MYL4, ANK1	168	113	12782	1.00000	0.90334	99.92544
GOTERM_CC_FAT	GO:0043292~contractile fiber	3	1.20482	1.88636	4.71E-01	TNNT2, MYL4, ANK1	168	121	12782	1.00000	0.91379	99.96669

Annotation Cluster 56 Enrichment Score: 0.3558487168090873

Category	Term	Count	%	Fold Enrich	PValue	Genes	List Total	Pop Hits	Pop Total	Bonferroni	Benjamini	FDR
GOTERM_CC_FAT	GO:0005792~microsome	6	2.40964	1.92616	1.99E-01	CYP2A13, VCP, AQP8,	168	237	12782	1.00000	0.77845	93.83891
GOTERM_CC_FAT	GO:0042598~vesicular fraction	6	2.40964	1.87090	2.15E-01	CYP2A13, VCP, AQP8,	168	244	12782	1.00000	0.77574	95.22095
SP_PIR_KEYWORDS	microsome	3	1.20482	2.21295	3.92E-01	CYP2A13, TAPBPL, CYI	246	106	19235	1.00000	0.92520	99.88712
GOTERM_CC_FAT	GO:0005624~membrane fraction	10	4.01606	0.94046	7.38E-01	CYP2A13, APP, VCP, F	168	809	12782	1.00000	0.98775	########
GOTERM_CC_FAT	GO:0000267~cell fraction	13	5.22088	0.91328	7.65E-01	AQP8, GNRH2, COMT,	168	1083	12782	1.00000	0.99017	########
GOTERM_CC_FAT	GO:0005626~insoluble fraction	10	4.01606	0.90683	7.75E-01	CYP2A13, APP, VCP, F	168	839	12782	1.00000	0.99078	########

Annotation Cluster 57 Enrichment Score: 0.32247011545797327

Category	Term	Count	%	Fold Enrich	PValue	Genes	List Total	Pop Hits	Pop Total	Bonferroni	Benjamini	FDR
GOTERM_BP_FAT	GO:0007254~JNK cascade	3	1.20482	3.71819	1.91E-01	RIPK1, DUSP10, MAPK	185	59	13528	1.00000	0.99692	96.74365
GOTERM_BP_FAT	GO:0031098~stress-activated protein kinase	3	1.20482	3.48211	2.11E-01	RIPK1, DUSP10, MAPK	185	63	13528	1.00000	0.99521	97.81845
GOTERM_BP_FAT	GO:0000165~MAPKKK cascade	3	1.20482	1.19224	7.17E-01	RIPK1, DUSP10, MAPK	185	184	13528	1.00000	0.99985	########
GOTERM_BP_FAT	GO:0007243~protein kinase cascade	4	1.60643	0.79053	8.83E-01	SGK2, RIPK1, DUSP10,	185	370	13528	1.00000	1.00000	########
GOTERM_BP_FAT	GO:0033554~cellular response to stress	5	2.00803	0.64597	9.53E-01	SP100, VCP, RIPK1, DU	185	566	13528	1.00000	1.00000	########

Annotation Cluster 58 Enrichment Score: 0.3198952403170382

Category	Term	Count	%	Fold Enrich	PValue	Genes	List Total	Pop Hits	Pop Total	Bonferroni	Benjamini	FDR
INTERPRO	IPR000210:BTB/POZ-like	4	1.60643	1.65453	4.31E-01	KLHL38, KCTD1, RHOB	225	179	16659	1.00000	0.99824	99.96888
INTERPRO	IPR011333:BTB/POZ fold	4	1.60643	1.63624	4.41E-01	KLHL38, KCTD1, RHOB	225	181	16659	1.00000	0.99833	99.97596
SMART	SM00225:BTB	4	1.60643	1.54872	4.75E-01	KLHL38, KCTD1, RHOB	131	179	9079	1.00000	0.99517	99.93538
INTERPRO	IPR013069:BTB/POZ	3	1.20482	1.74898	5.11E-01	KLHL38, RHOBTB2, BT	225	127	16659	1.00000	0.99947	99.99653
UP_SEQ_FEATURE	domain:BTB	3	1.20482	1.64145	5.46E-01	KLHL38, KCTD1, BTBD	246	142	19113	1.00000	1.00000	99.99950

Annotation Cluster 59 Enrichment Score: 0.30175832227998134

Category	Term	Count	%	Fold Enrich	PValue	Genes	List Total	Pop Hits	Pop Total	Bonferroni	Benjamini	FDR
SP_PIR_KEYWORDS	cell division	6	2.40964	1.77707	2.51E-01	SEPT4, CETN2, NEDD9	246	264	19235	1.00000	0.85195	98.05340
GOTERM_CC_FAT	GO:0005819~spindle	4	1.60643	2.07029	3.01E-01	APP, PLEKHG6, NEDD	168	147	12782	1.00000	0.83936	98.89858
SP_PIR_KEYWORDS	cell cycle	8	3.21285	1.35689	3.63E-01	SEPT4, CROCC, CDK2	246	461	19235	1.00000	0.91125	99.78597
GOTERM_BP_FAT	GO:0051301~cell division	6	2.40964	1.48727	3.75E-01	SEPT4, CETN2, NEDD9	185	295	13528	1.00000	0.99713	99.94884
GOTERM_BP_FAT	GO:0007049~cell cycle	12	4.81928	1.13079	4.88E-01	SEPT4, APP, CROCC, I	185	776	13528	1.00000	0.99802	99.99797
GOTERM_BP_FAT	GO:0022403~cell cycle phase	7	2.81124	1.23640	4.96E-01	APP, CDK2AP1, CETN2	185	414	13528	1.00000	0.99809	99.99839
GOTERM_BP_FAT	GO:0000278~mitotic cell cycle	6	2.40964	1.18580	5.69E-01	APP, CDK2AP1, CETN2	185	370	13528	1.00000	0.99907	99.99987
GOTERM_BP_FAT	GO:0000280~nuclear division	4	1.60643	1.32953	5.78E-01	CETN2, NEDD9, NEK6,	185	220	13528	1.00000	0.99910	99.99991
GOTERM_BP_FAT	GO:0007067~mitosis	4	1.60643	1.32953	5.78E-01	CETN2, NEDD9, NEK6,	185	220	13528	1.00000	0.99910	99.99991
GOTERM_BP_FAT	GO:0000087~M phase of mitotic cell cycle	4	1.60643	1.30579	5.91E-01	CETN2, NEDD9, NEK6,	185	224	13528	1.00000	0.99910	99.99994
GOTERM_BP_FAT	GO:0048285~organelle fission	4	1.60643	1.27728	6.06E-01	CETN2, NEDD9, NEK6,	185	229	13528	1.00000	0.99930	99.99997
GOTERM_BP_FAT	GO:0000279~M phase	5	2.00803	1.11131	6.58E-01	CETN2, NEDD9, LFNG,	185	329	13528	1.00000	0.99961	########
SP_PIR_KEYWORDS	mitosis	3	1.20482	1.28182	6.80E-01	CETN2, NEDD9, NEK6	246	183	19235	1.00000	0.98889	99.99998
GOTERM_BP_FAT	GO:0022402~cell cycle process	7	2.81124	0.90597	7.86E-01	APP, CDK2AP1, CETN2	185	565	13528	1.00000	0.99995	########

Annotation Cluster 60 Enrichment Score: 0.256997565562394

Category	Term	Count	%	Fold Enrich	PValue	Genes	List Total	Pop Hits	Pop Total	Bonferroni	Benjamini	FDR
GOTERM_BP_FAT	GO:0016310~phosphorylation	15	6.02410	1.37108	2.01E-01	SGK2, FGR, ATP6V1B1	185	800	13528	1.00000	0.99522	97.28533
INTERPRO	IPR001245:Tyrosine protein kinase	4	1.60643	2.50983	2.12E-01	FGR, RIPK1, NEK6, CSI	225	118	16659	1.00000	0.96968	96.71786

SMART	SM00219:TyrKc	4	1.60643	2.34933	2.39E-01	FGR, RIPK1, NEK6, CSI	131	118	9079	1.00000	0.95784	95.55803
SP_PIR_KEYWORDS	phosphotransferase	5	2.00803	1.94505	2.54E-01	GCK, FGR, RIPK1, PRK	246	201	19235	1.00000	0.85132	98.17908
GOTERM_BP_FAT	GO:0006468~protein amino acid phosphoryla	12	4.81928	1.31558	2.99E-01	SGK223, APP, SGK2, F	185	667	13528	1.00000	0.99663	99.67305
GOTERM_BP_FAT	GO:0006793~phosphorus metabolic process	16	6.42570	1.20246	3.45E-01	SGK2, FGR, DUSP10, A	185	973	13528	1.00000	0.99618	99.89147
GOTERM_BP_FAT	GO:0006796~phosphate metabolic process	16	6.42570	1.20246	3.45E-01	SGK2, FGR, DUSP10, A	185	973	13528	1.00000	0.99618	99.89147
GOTERM_MF_FAT	GO:0004713~protein tyrosine kinase activity	4	1.60643	1.81886	3.72E-01	SGK223, FGR, NEK6, C	172	166	12983	1.00000	0.99860	99.84762
INTERPRO	IPR008266:Tyrosine protein kinase, active sit	3	1.20482	2.28990	3.76E-01	SGK223, FGR, CSF1R	225	97	16659	1.00000	0.99616	99.88336
SP_PIR_KEYWORDS	transferase	20	8.03213	1.12182	4.14E-01	MOGAT2, SGK2, FGR, I	246	1394	19235	1.00000	0.93291	99.93145
SP_PIR_KEYWORDS	tyrosine-protein kinase	3	1.20482	2.11327	4.14E-01	SGK223, FGR, CSF1R	246	111	19235	1.00000	0.93051	99.93219
UP_SEQ_FEATURE	active site:Proton acceptor	10	4.01606	1.18078	4.67E-01	SGK223, SGK2, LDHAL	246	658	19113	1.00000	0.99998	99.99399
UP_SEQ_FEATURE	binding site:ATP	8	3.21285	1.14679	5.45E-01	SGK223, SGK2, GCK, F	246	542	19113	1.00000	1.00000	99.99949
UP_SEQ_FEATURE	domain:Protein kinase	7	2.81124	1.15963	5.59E-01	SGK223, SGK2, FGR, R	246	469	19113	1.00000	1.00000	99.99969
SP_PIR_KEYWORDS	nucleotide-binding	22	8.83534	1.02029	5.76E-01	GNAT1, SEPT4, SGK2,	246	1686	19235	1.00000	0.97391	99.99918
UP_SEQ_FEATURE	nucleotide phosphate-binding region:ATP	13	5.22088	1.04993	5.81E-01	SGK2, FGR, ABCB6, SC	246	962	19113	1.00000	1.00000	99.99985
GOTERM_MF_FAT	GO:0032559~adenyl ribonucleotide binding	20	8.03213	1.00845	6.05E-01	SGK2, FGR, TRPV1, MY	172	1497	12983	1.00000	0.99934	99.99975
INTERPRO	IPR000719:Protein kinase, core	7	2.81124	1.08882	6.21E-01	SGK223, SGK2, FGR, R	225	476	16659	1.00000	0.99993	99.99991
GOTERM_MF_FAT	GO:0001883~purine nucleoside binding	21	8.43373	0.99009	6.35E-01	GNAT1, SGK2, FGR, TF	172	1601	12983	1.00000	0.99938	99.99992
GOTERM_MF_FAT	GO:0032555~purine ribonucleotide binding	24	9.63855	0.98670	6.36E-01	GNAT1, SEPT4, SGK2,	172	1836	12983	1.00000	0.99930	99.99992
GOTERM_MF_FAT	GO:0032553~ribonucleotide binding	24	9.63855	0.98670	6.36E-01	GNAT1, SEPT4, SGK2,	172	1836	12983	1.00000	0.99930	99.99992
GOTERM_MF_FAT	GO:0001882~nucleoside binding	21	8.43373	0.98333	6.48E-01	GNAT1, SGK2, FGR, TF	172	1612	12983	1.00000	0.99918	99.99995
SP_PIR_KEYWORDS	kinase	9	3.61446	1.02285	6.53E-01	SGK223, SGK2, GCK, F	246	688	19235	1.00000	0.98621	99.99995
SP_PIR_KEYWORDS	magnesium	6	2.40964	1.06142	6.67E-01	ATP9A, ITGAE, COMT,	246	442	19235	1.00000	0.98767	99.99997
GOTERM_MF_FAT	GO:0004672~protein kinase activity	8	3.21285	0.99647	6.92E-01	SGK223, SGK2, FGR, R	172	606	12983	1.00000	0.99938	99.99999
GOTERM_MF_FAT	GO:0030554~adenyl nucleotide binding	20	8.03213	0.95729	6.96E-01	SGK2, FGR, TRPV1, MY	172	1577	12983	1.00000	0.99936	99.99999
GOTERM_MF_FAT	GO:0017076~purine nucleotide binding	24	9.63855	0.94452	7.20E-01	GNAT1, SEPT4, SGK2,	172	1918	12983	1.00000	0.99956	########
SP_PIR_KEYWORDS	atp-binding	16	6.42570	0.94348	7.20E-01	SGK2, FGR, MYO7B, TF	246	1326	19235	1.00000	0.99231	########
INTERPRO	IPR017441:Protein kinase, ATP binding site	6	2.40964	0.97635	7.36E-01	SGK223, SGK2, FGR, T	225	455	16659	1.00000	0.99999	########
GOTERM_MF_FAT	GO:0005524~ATP binding	18	7.22892	0.91990	7.58E-01	SGK2, FGR, MYO7B, TF	172	1477	12983	1.00000	0.99974	########
SP_PIR_KEYWORDS	ATP	3	1.20482	0.99395	8.05E-01	GCK, FGR, CSF1R	246	236	19235	1.00000	0.99642	########
GOTERM_MF_FAT	GO:0004674~protein serine/threonine kinase	5	2.00803	0.87770	8.23E-01	SGK2, RIPK1, ITGAE, T	172	430	12983	1.00000	0.99986	########
GOTERM_MF_FAT	GO:0000166~nucleotide binding	26	10.44177	0.87419	8.49E-01	SEPT4, FGR, TRPV1, M	172	2245	12983	1.00000	0.99991	########
GOTERM_MF_FAT	GO:0000287~magnesium ion binding	5	2.00803	0.83498	8.51E-01	ATP9A, ITGAE, COMT,	172	452	12983	1.00000	0.99991	########
INTERPRO	IPR008271:Serine/threonine protein kinase, a	4	1.60643	0.83661	8.58E-01	SGK2, RIPK1, TSSK4, N	225	354	16659	1.00000	1.00000	########
INTERPRO	IPR017442:Serine/threonine protein kinase-re	4	1.60643	0.82496	8.65E-01	SGK2, RIPK1, TSSK4, N	225	359	16659	1.00000	1.00000	########
INTERPRO	IPR002290:Serine/threonine protein kinase	3	1.20482	0.85761	8.66E-01	SGK2, TSSK4, NEK6	225	259	16659	1.00000	1.00000	########
SP_PIR_KEYWORDS	serine/threonine-protein kinase	4	1.60643	0.82090	8.67E-01	SGK2, RIPK1, TSSK4, N	246	381	19235	1.00000	0.99880	########
SMART	SM00220:S_TKc	3	1.20482	0.80276	8.90E-01	SGK2, TSSK4, NEK6	131	259	9079	1.00000	1.00000	########

Annotation Cluster 61 Enrichment Score: 0.2384226590198531

Category	Term	Count	%	Fold Enrich	PValue	Genes	List Total	Pop Hits	Pop Total	Bonferroni	Benjamini	FDR
SP_PIR_KEYWORDS	Protease	8	3.21285	1.29241	4.30E-01	APP, MMP21, KLK3, AD	246	484	19235	1.00000	0.93668	99.95289
GOTERM_MF_FAT	GO:0008233~peptidase activity	9	3.61446	1.18352	4.87E-01	APP, MMP21, KLK3, AD	172	574	12983	1.00000	0.99919	99.99067
SP_PIR_KEYWORDS	zymogen	4	1.60643	1.51827	4.90E-01	MMP21, KLK3, ADAM3C	246	206	19235	1.00000	0.95440	99.98973
SP_PIR_KEYWORDS	Serine protease	3	1.20482	1.72480	5.19E-01	KLK3, RHBDL2, C1S	246	136	19235	1.00000	0.96196	99.99538
GOTERM_MF_FAT	GO:0004175~endopeptidase activity	6	2.40964	1.20772	5.52E-01	MMP21, KLK3, ADAM3C	172	375	12983	1.00000	0.99938	99.99862
GOTERM_MF_FAT	GO:0004252~serine-type endopeptidase activ	3	1.20482	1.47044	6.05E-01	KLK3, RHBDL2, C1S	172	154	12983	1.00000	0.99925	99.99976
GOTERM_MF_FAT	GO:0008236~serine-type peptidase activity	3	1.20482	1.27218	6.83E-01	KLK3, RHBDL2, C1S	172	178	12983	1.00000	0.99955	99.99999
GOTERM_MF_FAT	GO:0017171~serine hydrolase activity	3	1.20482	1.25804	6.89E-01	KLK3, RHBDL2, C1S	172	180	12983	1.00000	0.99948	99.99999
GOTERM_MF_FAT	GO:0070011~peptidase activity, acting on L-a	6	2.40964	0.82495	8.55E-01	MMP21, KLK3, ADAM3C	172	549	12983	1.00000	0.99991	########

Annotation Cluster 62 Enrichment Score: 0.23038944039136383

Category	Term	Count	%	Fold Enrich	PValue	Genes	List Total	Pop Hits	Pop Total	Bonferroni	Benjamini	FDR
GOTERM_BP_FAT	GO:0007610~behavior	9	3.61446	1.40324	3.07E-01	MCHR1, APP, CCR6, IL	185	469	13528	1.00000	0.99675	99.73110
GOTERM_BP_FAT	GO:0019226~transmission of nerve impulse	5	2.00803	1.04463	7.05E-01	KCNMB3, APP, P2RX3,	185	350	13528	1.00000	0.99983	########
GOTERM_BP_FAT	GO:0007267~cell-cell signaling	8	3.21285	0.97499	7.13E-01	APP, PTGIR, P2RX3, TS	185	600	13528	1.00000	0.99985	########
GOTERM_BP_FAT	GO:0007268~synaptic transmission	4	1.60643	0.98153	7.75E-01	APP, P2RX3, APBA2, C	185	298	13528	1.00000	0.99994	########

Annotation Cluster 63	Enrichment Score: 0.19092556837955257											
Category	Term	Count	%	Fold Enrich	PValue	Genes	List Total	Pop Hits	Pop Total	Bonferroni	Benjamini	FDR
GOTERM_BP_FAT	GO:0010324~membrane invagination	4	1.60643	1.32953	5.78E-01	ASGR2, APP, FCN2, CC	185	220	13528	1.00000	0.99910	99.99991
GOTERM_BP_FAT	GO:0006897~endocytosis	4	1.60643	1.32953	5.78E-01	ASGR2, APP, FCN2, CC	185	220	13528	1.00000	0.99910	99.99991
GOTERM_BP_FAT	GO:0016192~vesicle-mediated transport	8	3.21285	1.01562	6.73E-01	PREB, ASGR2, APP, AN	185	576	13528	1.00000	0.99970	########
GOTERM_BP_FAT	GO:0016044~membrane organization	5	2.00803	0.95964	7.66E-01	PREB, ASGR2, APP, FC	185	381	13528	1.00000	0.99993	########

Annotation Cluster 64	Enrichment Score: 0.17154057874361137											
Category	Term	Count	%	Fold Enrich	PValue	Genes	List Total	Pop Hits	Pop Total	Bonferroni	Benjamini	FDR
GOTERM_BP_FAT	GO:0007283~spermatogenesis	5	2.00803	1.18708	6.07E-01	TXNDC2, CETN2, AQP7	185	308	13528	1.00000	0.99928	99.99997
GOTERM_BP_FAT	GO:0048232~male gamete generation	5	2.00803	1.18708	6.07E-01	TXNDC2, CETN2, AQP7	185	308	13528	1.00000	0.99928	99.99997
GOTERM_BP_FAT	GO:0048609~reproductive process in a multic	7	2.81124	1.05107	6.54E-01	TXNDC2, CETN2, AQP7	185	487	13528	1.00000	0.99960	########
GOTERM_BP_FAT	GO:0032504~multicellular organism reproduc	7	2.81124	1.05107	6.54E-01	TXNDC2, CETN2, AQP7	185	487	13528	1.00000	0.99960	########
GOTERM_BP_FAT	GO:0019953~sexual reproduction	6	2.40964	0.95796	7.51E-01	DNALI1, TXNDC2, CETN	185	458	13528	1.00000	0.99991	########
GOTERM_BP_FAT	GO:0007276~gamete generation	5	2.00803	0.92562	7.90E-01	TXNDC2, CETN2, AQP7	185	395	13528	1.00000	0.99995	########

Annotation Cluster 65	Enrichment Score: 0.15747117835020613											
Category	Term	Count	%	Fold Enrich	PValue	Genes	List Total	Pop Hits	Pop Total	Bonferroni	Benjamini	FDR
INTERPRO	IPR015943:WD40/YVTN repeat-like	6	2.40964	1.48080	3.77E-01	PREB, SEMA4G, PLXNI	225	300	16659	1.00000	0.99573	99.88719
UP_SEQ_FEATURE	repeat:WD 3	4	1.60643	1.17276	6.63E-01	PREB, DCAF12L1, FBX\	246	265	19113	1.00000	1.00000	########
UP_SEQ_FEATURE	repeat:WD 1	4	1.60643	1.13424	6.85E-01	PREB, DCAF12L1, FBX\	246	274	19113	1.00000	1.00000	########
UP_SEQ_FEATURE	repeat:WD 2	4	1.60643	1.13424	6.85E-01	PREB, DCAF12L1, FBX\	246	274	19113	1.00000	1.00000	########
SP_PIR_KEYWORDS	wd repeat	4	1.60643	1.13320	6.86E-01	PREB, DCAF12L1, FBX\	246	276	19235	1.00000	0.98917	99.99999
INTERPRO	IPR019775:WD40 repeat, conserved site	4	1.60643	1.06917	7.23E-01	PREB, DCAF12L1, FBX\	225	277	16659	1.00000	0.99999	########
UP_SEQ_FEATURE	repeat:WD 4	3	1.20482	0.92863	8.35E-01	DCAF12L1, FBXW12, PI	246	251	19113	1.00000	1.00000	########
INTERPRO	IPR001680:WD40 repeat	3	1.20482	0.83504	8.76E-01	PREB, DCAF12L1, PPP:	225	266	16659	1.00000	1.00000	########
SMART	SM00320:WD40	3	1.20482	0.78164	8.99E-01	PREB, DCAF12L1, PPP:	131	266	9079	1.00000	1.00000	########

Annotation Cluster 66	Enrichment Score: 0.15173013453018194											
Category	Term	Count	%	Fold Enrich	PValue	Genes	List Total	Pop Hits	Pop Total	Bonferroni	Benjamini	FDR
GOTERM_CC_FAT	GO:0030141~secretory granule	5	2.00803	2.11343	2.09E-01	APP, SPACA3, LACRT,	168	180	12782	1.00000	0.77505	94.72788
GOTERM_CC_FAT	GO:0044433~cytoplasmic vesicle part	3	1.20482	1.22059	7.05E-01	APP, CUZD1, SERPINA	168	187	12782	1.00000	0.98533	99.99998
GOTERM_CC_FAT	GO:0016023~cytoplasmic membrane-bounde	5	2.00803	0.69167	9.33E-01	APP, SPACA3, LACRT,	168	550	12782	1.00000	0.99953	########
GOTERM_CC_FAT	GO:0031988~membrane-bounded vesicle	5	2.00803	0.66975	9.43E-01	APP, SPACA3, LACRT,	168	568	12782	1.00000	0.99966	########
GOTERM_CC_FAT	GO:0031410~cytoplasmic vesicle	5	2.00803	0.59255	9.71E-01	APP, SPACA3, LACRT,	168	642	12782	1.00000	0.99991	########
GOTERM_CC_FAT	GO:0031982~vesicle	5	2.00803	0.56779	9.78E-01	APP, SPACA3, LACRT,	168	670	12782	1.00000	0.99995	########

Annotation Cluster 67	Enrichment Score: 0.13514567945625372											
Category	Term	Count	%	Fold Enrich	PValue	Genes	List Total	Pop Hits	Pop Total	Bonferroni	Benjamini	FDR
GOTERM_BP_FAT	GO:0006928~cell motion	7	2.81124	1.07762	6.31E-01	APP, DNALI1, CCR6, IL:	185	475	13528	1.00000	0.99948	99.99999
GOTERM_BP_FAT	GO:0016477~cell migration	4	1.60643	1.05977	7.29E-01	IL16, ARHGEF7, PSG2,	185	276	13528	1.00000	0.99987	########
GOTERM_BP_FAT	GO:0051674~localization of cell	4	1.60643	0.95276	7.92E-01	IL16, ARHGEF7, PSG2,	185	307	13528	1.00000	0.99995	########
GOTERM_BP_FAT	GO:0048870~cell motility	4	1.60643	0.95276	7.92E-01	IL16, ARHGEF7, PSG2,	185	307	13528	1.00000	0.99995	########

Annotation Cluster 68	Enrichment Score: 0.13501951054469422											
Category	Term	Count	%	Fold Enrich	PValue	Genes	List Total	Pop Hits	Pop Total	Bonferroni	Benjamini	FDR
INTERPRO	IPR008957:Fibronectin, type III-like fold	3	1.20482	1.20717	7.11E-01	MYBPH, COL12A1, MYE	225	184	16659	1.00000	0.99999	########
INTERPRO	IPR003961:Fibronectin, type III	3	1.20482	1.16905	7.28E-01	MYBPH, COL12A1, MYE	225	190	16659	1.00000	0.99999	########
SMART	SM00060:FN3	3	1.20482	1.09429	7.61E-01	MYBPH, COL12A1, MYE	131	190	9079	1.00000	0.99997	99.99999

Annotation Cluster 69	Enrichment Score: 0.11638516720182036											
Category	Term	Count	%	Fold Enrich	PValue	Genes	List Total	Pop Hits	Pop Total	Bonferroni	Benjamini	FDR
GOTERM_BP_FAT	GO:0001932~regulation of protein amino acid	4	1.60643	1.69074	4.15E-01	RIPK1, PRKAR1B, LACI	185	173	13528	1.00000	0.99715	99.98228
GOTERM_BP_FAT	GO:0042325~regulation of phosphorylation	6	2.40964	0.94151	7.64E-01	APP, PPP1R14D, RIPK1	185	466	13528	1.00000	0.99993	########
GOTERM_BP_FAT	GO:0031399~regulation of protein modificatio	4	1.60643	0.99152	7.69E-01	RIPK1, PRKAR1B, LACI	185	295	13528	1.00000	0.99993	########
GOTERM_BP_FAT	GO:0019220~regulation of phosphate metabo	6	2.40964	0.90463	7.94E-01	APP, PPP1R14D, RIPK1	185	485	13528	1.00000	0.99995	########
GOTERM_BP_FAT	GO:0051174~regulation of phosphorus metab	6	2.40964	0.90463	7.94E-01	APP, PPP1R14D, RIPK1	185	485	13528	1.00000	0.99995	########

GOTERM_BP_FAT	GO:0045859~regulation of protein kinase acti	4	1.60643	0.84782	8.52E-01	APP, RIPK1, PRKAR1B,	185	345	13528	1.00000	0.99999	########
GOTERM_BP_FAT	GO:0043549~regulation of kinase activity	4	1.60643	0.81932	8.68E-01	APP, RIPK1, PRKAR1B,	185	357	13528	1.00000	0.99999	########
GOTERM_BP_FAT	GO:0051338~regulation of transferase activity	4	1.60643	0.78628	8.85E-01	APP, RIPK1, PRKAR1B,	185	372	13528	1.00000	1.00000	########
GOTERM_BP_FAT	GO:0032268~regulation of cellular protein me	5	2.00803	0.77135	8.90E-01	APP, RIPK1, PRKAR1B,	185	474	13528	1.00000	1.00000	########

Annotation Cluster 70 Enrichment Score: 0.11397802536112175

Category	Term	Count	%	Fold Enrich	PValue	Genes	List Total	Pop Hits	Pop Total	Bonferroni	Benjamini	FDR
UP_SEQ_FEATURE	repeat:LRR 3	4	1.60643	1.03940	7.41E-01	ISLR, SYNE2, PRAMEF	246	299	19113	1.00000	1.00000	########
UP_SEQ_FEATURE	repeat:LRR 1	4	1.60643	0.97730	7.77E-01	ISLR, SYNE2, PRAMEF	246	318	19113	1.00000	1.00000	########
UP_SEQ_FEATURE	repeat:LRR 2	4	1.60643	0.97423	7.79E-01	ISLR, SYNE2, PRAMEF	246	319	19113	1.00000	1.00000	########
SP_PIR_KEYWORDS	leucine-rich repeat	4	1.60643	0.97132	7.81E-01	ISLR, SYNE2, PRAMEF	246	322	19235	1.00000	0.99589	########

Annotation Cluster 71 Enrichment Score: 0.09543261880748943

Category	Term	Count	%	Fold Enrich	PValue	Genes	List Total	Pop Hits	Pop Total	Bonferroni	Benjamini	FDR
GOTERM_MF_FAT	GO:0042802~identical protein binding	8	3.21285	0.94353	7.45E-01	APP, CLDN18, SP100, T	172	640	12983	1.00000	0.99968	########
GOTERM_MF_FAT	GO:0042803~protein homodimerization activi	4	1.60643	0.90398	8.20E-01	SP100, THRA, P2RX3, (	172	334	12983	1.00000	0.99987	########
GOTERM_MF_FAT	GO:0046983~protein dimerization activity	6	2.40964	0.83560	8.47E-01	JDP2, SP100, THRA, P2	172	542	12983	1.00000	0.99992	########

Annotation Cluster 72 Enrichment Score: 0.09323713945277244

Category	Term	Count	%	Fold Enrich	PValue	Genes	List Total	Pop Hits	Pop Total	Bonferroni	Benjamini	FDR
GOTERM_BP_FAT	GO:0051259~protein oligomerization	4	1.60643	1.68102	4.19E-01	VCP, TRPV5, COLEC12	185	174	13528	1.00000	0.99721	99.98414
GOTERM_BP_FAT	GO:0070271~protein complex biogenesis	5	2.00803	0.72400	9.17E-01	VCP, TRPV5, COLEC12	185	505	13528	1.00000	1.00000	########
GOTERM_BP_FAT	GO:0006461~protein complex assembly	5	2.00803	0.72400	9.17E-01	VCP, TRPV5, COLEC12	185	505	13528	1.00000	1.00000	########
GOTERM_BP_FAT	GO:0065003~macromolecular complex asser	5	2.00803	0.54981	9.82E-01	VCP, TRPV5, COLEC12	185	665	13528	1.00000	1.00000	########
GOTERM_BP_FAT	GO:0043933~macromolecular complex subur	5	2.00803	0.51496	9.89E-01	VCP, TRPV5, COLEC12	185	710	13528	1.00000	1.00000	########

Annotation Cluster 73 Enrichment Score: 0.09171881125317635

Category	Term	Count	%	Fold Enrich	PValue	Genes	List Total	Pop Hits	Pop Total	Bonferroni	Benjamini	FDR
GOTERM_BP_FAT	GO:0051345~positive regulation of hydrolase	3	1.20482	1.22555	7.03E-01	TNNT2, MYL4, VCP	185	179	13528	1.00000	0.99983	########
GOTERM_BP_FAT	GO:0044093~positive regulation of molecular	7	2.81124	0.87350	8.14E-01	TNNT2, MYL4, SP100, \	185	586	13528	1.00000	0.99997	########
GOTERM_BP_FAT	GO:0043085~positive regulation of catalytic a	5	2.00803	0.70312	9.27E-01	TNNT2, MYL4, VCP, RIF	185	520	13528	1.00000	1.00000	########

Annotation Cluster 74 Enrichment Score: 0.08821045522597847

Category	Term	Count	%	Fold Enrich	PValue	Genes	List Total	Pop Hits	Pop Total	Bonferroni	Benjamini	FDR
GOTERM_BP_FAT	GO:0006917~induction of apoptosis	5	2.00803	1.14257	6.37E-01	FGD2, APP, ARHGEF7,	185	320	13528	1.00000	0.99950	99.99999
GOTERM_BP_FAT	GO:0012502~induction of programmed cell de	5	2.00803	1.13901	6.39E-01	FGD2, APP, ARHGEF7,	185	321	13528	1.00000	0.99950	99.99999
GOTERM_BP_FAT	GO:0043065~positive regulation of apoptosis	5	2.00803	0.85028	8.41E-01	FGD2, APP, ARHGEF7,	185	430	13528	1.00000	0.99999	########
GOTERM_BP_FAT	GO:0043068~positive regulation of programm	5	2.00803	0.84439	8.45E-01	FGD2, APP, ARHGEF7,	185	433	13528	1.00000	0.99999	########
GOTERM_BP_FAT	GO:0010942~positive regulation of cell death	5	2.00803	0.84051	8.47E-01	FGD2, APP, ARHGEF7,	185	435	13528	1.00000	0.99999	########
GOTERM_BP_FAT	GO:0042981~regulation of apoptosis	8	3.21285	0.72761	9.26E-01	FGD2, APP, VCP, ARH(	185	804	13528	1.00000	1.00000	########
GOTERM_BP_FAT	GO:0043067~regulation of programmed cell c	8	3.21285	0.72044	9.31E-01	FGD2, APP, VCP, ARH(	185	812	13528	1.00000	1.00000	########
GOTERM_BP_FAT	GO:0010941~regulation of cell death	8	3.21285	0.71778	9.32E-01	FGD2, APP, VCP, ARH(	185	815	13528	1.00000	1.00000	########

Annotation Cluster 75 Enrichment Score: 0.08616135533222814

Category	Term	Count	%	Fold Enrich	PValue	Genes	List Total	Pop Hits	Pop Total	Bonferroni	Benjamini	FDR
UP_SEQ_FEATURE	nucleotide phosphate-binding region:GTP	4	1.60643	1.05349	7.32E-01	GNAT1, SEPT4, RHOBT	246	295	19113	1.00000	1.00000	########
GOTERM_MF_FAT	GO:0003924~GTPase activity	3	1.20482	1.07321	7.70E-01	GNAT1, SEPT4, SEPT9	172	211	12983	1.00000	0.99975	########
SP_PIR_KEYWORDS	gtp-binding	4	1.60643	0.95065	7.93E-01	GNAT1, SEPT4, RHOBT	246	329	19235	1.00000	0.99645	########
GOTERM_MF_FAT	GO:0005525~GTP binding	4	1.60643	0.81164	8.72E-01	GNAT1, SEPT4, RHOBT	172	372	12983	1.00000	0.99994	########
GOTERM_MF_FAT	GO:0019001~guanyl nucleotide binding	4	1.60643	0.79039	8.83E-01	GNAT1, SEPT4, RHOBT	172	382	12983	1.00000	0.99996	########
GOTERM_MF_FAT	GO:0032561~guanyl ribonucleotide binding	4	1.60643	0.79039	8.83E-01	GNAT1, SEPT4, RHOBT	172	382	12983	1.00000	0.99996	########

Annotation Cluster 76 Enrichment Score: 0.07772291798072627

Category	Term	Count	%	Fold Enrich	PValue	Genes	List Total	Pop Hits	Pop Total	Bonferroni	Benjamini	FDR
GOTERM_MF_FAT	GO:0009055~electron carrier activity	3	1.20482	1.02465	7.92E-01	CYP2A13, ALDH4A1, CY	172	221	12983	1.00000	0.99984	########
SP_PIR_KEYWORDS	oxidoreductase	6	2.40964	0.83478	8.47E-01	CYP2A13, LDHAL6B, AI	246	562	19235	1.00000	0.99821	########
GOTERM_BP_FAT	GO:0055114~oxidation reduction	7	2.81124	0.80105	8.72E-01	CYP2A13, LDHAL6B, AI	185	639	13528	1.00000	0.99999	########

Annotation Cluster 77 Enrichment Score: 0.06282467897970034

Category	Term	Count	%	Fold Enrich	PValue	Genes	List Total	Pop Hits	Pop Total	Bonferroni	Benjamini	FDR
GOTERM_BP_FAT	GO:0051046~regulation of secretion	3	1.20482	1.08600	7.64E-01	GCK, LACRT, NEUROD	185	202	13528	1.00000	0.99993	########
GOTERM_BP_FAT	GO:0060341~regulation of cellular localization	3	1.20482	0.88457	8.54E-01	GCK, LACRT, NEUROD	185	248	13528	1.00000	0.99999	########
GOTERM_BP_FAT	GO:0010604~positive regulation of macromol	6	2.40964	0.51196	9.92E-01	APP, SP100, THRA, GC	185	857	13528	1.00000	1.00000	########

Annotation Cluster 78 Enrichment Score: 0.05970860988235009

Category	Term	Count	%	Fold Enrich	PValue	Genes	List Total	Pop Hits	Pop Total	Bonferroni	Benjamini	FDR
SP_PIR_KEYWORDS	protein transport	6	2.40964	0.96731	7.43E-01	PREB, ACAP1, SNX8, A	246	485	19235	1.00000	0.99414	########
GOTERM_BP_FAT	GO:0008104~protein localization	10	4.01606	0.82907	8.55E-01	TNS4, PREB, PPY, VCP	185	882	13528	1.00000	0.99999	########
GOTERM_BP_FAT	GO:0015031~protein transport	7	2.81124	0.67175	9.51E-01	PREB, PPY, VCP, ACAP	185	762	13528	1.00000	1.00000	########
GOTERM_BP_FAT	GO:0045184~establishment of protein localiza	7	2.81124	0.66563	9.54E-01	PREB, PPY, VCP, ACAP	185	769	13528	1.00000	1.00000	########

Annotation Cluster 79 Enrichment Score: 0.056039274662799544

Category	Term	Count	%	Fold Enrich	PValue	Genes	List Total	Pop Hits	Pop Total	Bonferroni	Benjamini	FDR
UP_SEQ_FEATURE	short sequence motif:Nuclear localization sigr	7	2.81124	1.67343	2.42E-01	TCF20, SP100, SGK2, N	246	325	19113	1.00000	0.99716	98.61416
SP_PIR_KEYWORDS	activator	6	2.40964	0.90220	7.95E-01	PREB, MEAF6, TCF20,	246	520	19235	1.00000	0.99619	########
GOTERM_MF_FAT	GO:0043565~sequence-specific DNA binding	7	2.81124	0.87047	8.17E-01	JDP2, THRA, FLI1, NEU	172	607	12983	1.00000	0.99988	########
SP_PIR_KEYWORDS	DNA binding	3	1.20482	0.68992	9.33E-01	THRA, FLI1, RORC	246	340	19235	1.00000	0.99983	########
GOTERM_MF_FAT	GO:0003700~transcription factor activity	7	2.81124	0.54193	9.91E-01	JDP2, THRA, FLI1, NEU	172	975	12983	1.00000	1.00000	########
GOTERM_MF_FAT	GO:0030528~transcription regulator activity	10	4.01606	0.49922	9.99E-01	PREB, TCF20, JDP2, SI	172	1512	12983	1.00000	1.00000	########
GOTERM_BP_FAT	GO:0006355~regulation of transcription, DNA	13	5.22088	0.53616	9.99E-01	JDP2, THRA, SP100, R(	185	1773	13528	1.00000	1.00000	########
GOTERM_BP_FAT	GO:0051252~regulation of RNA metabolic prc	13	5.22088	0.52433	9.99E-01	JDP2, THRA, SP100, R(	185	1813	13528	1.00000	1.00000	########
SP_PIR_KEYWORDS	dna-binding	12	4.81928	0.50230	9.99E-01	PREB, TCF20, JDP2, SI	246	1868	19235	1.00000	1.00000	########
SP_PIR_KEYWORDS	transcription regulation	12	4.81928	0.46313	1.00E+00	PREB, MEAF6, TCF20, .	246	2026	19235	1.00000	1.00000	########
GOTERM_BP_FAT	GO:0045449~regulation of transcription	19	7.63052	0.53416	1.00E+00	MEAF6, JDP2, THRA, S	185	2601	13528	1.00000	1.00000	########
SP_PIR_KEYWORDS	Transcription	12	4.81928	0.45306	1.00E+00	PREB, MEAF6, TCF20, .	246	2071	19235	1.00000	1.00000	########
GOTERM_MF_FAT	GO:0003677~DNA binding	15	6.02410	0.48573	1.00E+00	JDP2, THRA, SP100, AI	172	2331	12983	1.00000	1.00000	########
SP_PIR_KEYWORDS	nucleus	32	12.85141	0.58420	1.00E+00	MEAF6, SEPT4, JDP2, ˜	246	4283	19235	1.00000	1.00000	########
GOTERM_BP_FAT	GO:0006350~transcription	12	4.81928	0.41765	1.00E+00	PREB, MEAF6, TCF20, .	185	2101	13528	1.00000	1.00000	########

Annotation Cluster 80 Enrichment Score: 0.051628304333218834

Category	Term	Count	%	Fold Enrich	PValue	Genes	List Total	Pop Hits	Pop Total	Bonferroni	Benjamini	FDR
GOTERM_CC_FAT	GO:0005635~nuclear envelope	3	1.20482	1.11341	7.52E-01	SLC2A9, DNASE1, SYN	168	205	12782	1.00000	0.98904	########
GOTERM_CC_FAT	GO:0031967~organelle envelope	5	2.00803	0.61358	9.65E-01	SLC2A9, DNASE1, TME	168	620	12782	1.00000	0.99987	########
GOTERM_CC_FAT	GO:0031975~envelope	5	2.00803	0.61160	9.65E-01	SLC2A9, DNASE1, TME	168	622	12782	1.00000	0.99986	########

Annotation Cluster 81 Enrichment Score: 0.015703460336288787

Category	Term	Count	%	Fold Enrich	PValue	Genes	List Total	Pop Hits	Pop Total	Bonferroni	Benjamini	FDR
GOTERM_BP_FAT	GO:0006397~mRNA processing	3	1.20482	0.68340	9.35E-01	APOBEC2, APP, CELF2	185	321	13528	1.00000	1.00000	########
GOTERM_BP_FAT	GO:0016071~mRNA metabolic process	3	1.20482	0.59290	9.63E-01	APOBEC2, APP, CELF2	185	370	13528	1.00000	1.00000	########
GOTERM_BP_FAT	GO:0006396~RNA processing	3	1.20482	0.40105	9.96E-01	APOBEC2, APP, CELF2	185	547	13528	1.00000	1.00000	########

Annotation Cluster 82 Enrichment Score: 0.01523364117875514

Category	Term	Count	%	Fold Enrich	PValue	Genes	List Total	Pop Hits	Pop Total	Bonferroni	Benjamini	FDR
GOTERM_MF_FAT	GO:0016563~transcription activator activity	5	2.00803	0.92052	7.93E-01	PREB, TCF20, SP100, T	172	410	12983	1.00000	0.99982	########
GOTERM_MF_FAT	GO:0046983~protein dimerization activity	6	2.40964	0.83560	8.47E-01	JDP2, SP100, THRA, P2	172	542	12983	1.00000	0.99992	########
GOTERM_MF_FAT	GO:0016564~transcription repressor activity	3	1.20482	0.71661	9.23E-01	JDP2, SP100, THRA	172	316	12983	1.00000	0.99999	########
GOTERM_BP_FAT	GO:0045893~positive regulation of transcripti	4	1.60643	0.61320	9.60E-01	APP, SP100, THRA, NE	185	477	13528	1.00000	1.00000	########
GOTERM_BP_FAT	GO:0051254~positive regulation of RNA meta	4	1.60643	0.60810	9.62E-01	APP, SP100, THRA, NE	185	481	13528	1.00000	1.00000	########
GOTERM_BP_FAT	GO:0045944~positive regulation of transcripti	3	1.20482	0.59130	9.64E-01	APP, THRA, NEUROD1	185	371	13528	1.00000	1.00000	########
GOTERM_BP_FAT	GO:0051172~negative regulation of nitrogen (	4	1.60643	0.56358	9.74E-01	JDP2, SP100, THRA, C(	185	519	13528	1.00000	1.00000	########
GOTERM_BP_FAT	GO:0051173~positive regulation of nitrogen c	5	2.00803	0.56774	9.78E-01	APP, SP100, THRA, NE	185	644	13528	1.00000	1.00000	########
GOTERM_BP_FAT	GO:0010557~positive regulation of macromol	5	2.00803	0.55905	9.80E-01	APP, SP100, THRA, GC	185	654	13528	1.00000	1.00000	########
GOTERM_BP_FAT	GO:0031327~negative regulation of cellular b	4	1.60643	0.52139	9.84E-01	JDP2, SP100, THRA, G(	185	561	13528	1.00000	1.00000	########
GOTERM_BP_FAT	GO:0045941~positive regulation of transcripti	4	1.60643	0.51861	9.84E-01	APP, SP100, THRA, NE	185	564	13528	1.00000	1.00000	########

GOTERM_BP_FAT	GO:0031328~positive regulation of cellular bi	5	2.00803	0.53375	9.85E-01	APP, SP100, THRA, GC	185	685	13528	1.00000	1.00000	########
GOTERM_BP_FAT	GO:0009890~negative regulation of biosynthe	4	1.60643	0.51047	9.86E-01	JDP2, SP100, THRA, G	185	573	13528	1.00000	1.00000	########
GOTERM_BP_FAT	GO:0009891~positive regulation of biosynthe	5	2.00803	0.52607	9.87E-01	APP, SP100, THRA, GC	185	695	13528	1.00000	1.00000	########
GOTERM_BP_FAT	GO:0010628~positive regulation of gene expr	4	1.60643	0.50344	9.87E-01	APP, SP100, THRA, NE	185	581	13528	1.00000	1.00000	########
GOTERM_BP_FAT	GO:0016481~negative regulation of transcript	3	1.20482	0.47794	9.87E-01	JDP2, SP100, THRA	185	459	13528	1.00000	1.00000	########
GOTERM_BP_FAT	GO:0006357~regulation of transcription from	5	2.00803	0.50292	9.91E-01	APP, JDP2, SP100, THF	185	727	13528	1.00000	1.00000	########
GOTERM_BP_FAT	GO:0045935~positive regulation of nucleobas	4	1.60643	0.46875	9.92E-01	APP, SP100, THRA, NE	185	624	13528	1.00000	1.00000	########
GOTERM_BP_FAT	GO:0010604~positive regulation of macromol	6	2.40964	0.51196	9.92E-01	APP, SP100, THRA, GC	185	857	13528	1.00000	1.00000	########
GOTERM_BP_FAT	GO:0010629~negative regulation of gene exp	3	1.20482	0.43526	9.93E-01	JDP2, SP100, THRA	185	504	13528	1.00000	1.00000	########
GOTERM_BP_FAT	GO:0045934~negative regulation of nucleoba	3	1.20482	0.42846	9.93E-01	JDP2, SP100, THRA	185	512	13528	1.00000	1.00000	########
GOTERM_BP_FAT	GO:0010558~negative regulation of macromo	3	1.20482	0.40105	9.96E-01	JDP2, SP100, THRA	185	547	13528	1.00000	1.00000	########
GOTERM_BP_FAT	GO:0010605~negative regulation of macromo	3	1.20482	0.29887	1.00E+00	JDP2, SP100, THRA	185	734	13528	1.00000	1.00000	########

Annotation Cluster 83 Enrichment Score: 0.011518328251924394

Category	Term	Count	%	Fold Enrich	PValue	Genes	List Total	Pop Hits	Pop Total	Bonferroni	Benjamini	FDR
GOTERM_BP_FAT	GO:0006508~proteolysis	11	4.41767	0.76316	9.16E-01	MMP21, VCP, KLK3, FB	185	1054	13528	1.00000	1.00000	########
GOTERM_BP_FAT	GO:0009057~macromolecule catabolic proce	7	2.81124	0.65540	9.59E-01	DNASE1, SPACA3, VCF	185	781	13528	1.00000	1.00000	########
SP_PIR_KEYWORDS	ubl conjugation pathway	4	1.60643	0.61447	9.59E-01	VCP, FBXO17, HERC6,	246	509	19235	1.00000	0.99996	########
GOTERM_BP_FAT	GO:0019941~modification-dependent protein	4	1.60643	0.50958	9.86E-01	VCP, FBXO17, HERC6,	185	574	13528	1.00000	1.00000	########
GOTERM_BP_FAT	GO:0043632~modification-dependent macron	4	1.60643	0.50958	9.86E-01	VCP, FBXO17, HERC6,	185	574	13528	1.00000	1.00000	########
GOTERM_BP_FAT	GO:0051603~proteolysis involved in cellular p	4	1.60643	0.48750	9.90E-01	VCP, FBXO17, HERC6,	185	600	13528	1.00000	1.00000	########
GOTERM_BP_FAT	GO:0044257~cellular protein catabolic proces	4	1.60643	0.48507	9.90E-01	VCP, FBXO17, HERC6,	185	603	13528	1.00000	1.00000	########
GOTERM_BP_FAT	GO:0044265~cellular macromolecule cataboli	5	2.00803	0.50431	9.90E-01	DNASE1, VCP, FBXO17	185	725	13528	1.00000	1.00000	########
GOTERM_BP_FAT	GO:0030163~protein catabolic process	4	1.60643	0.47025	9.92E-01	VCP, FBXO17, HERC6,	185	622	13528	1.00000	1.00000	########

Annotation Cluster 84 Enrichment Score: 0.0027689702304179047

Category	Term	Count	%	Fold Enrich	PValue	Genes	List Total	Pop Hits	Pop Total	Bonferroni	Benjamini	FDR
GOTERM_CC_FAT	GO:0044451~nucleoplasm part	4	1.60643	0.54835	9.78E-01	BRD1, MEAF6, CCR6, S	168	555	12782	1.00000	0.99994	########
GOTERM_CC_FAT	GO:0005730~nucleolus	5	2.00803	0.54501	9.83E-01	MEAF6, SP100, VCP, N	168	698	12782	1.00000	0.99997	########
GOTERM_CC_FAT	GO:0005654~nucleoplasm	5	2.00803	0.43131	9.98E-01	BRD1, MEAF6, CCR6, S	168	882	12782	1.00000	1.00000	########
GOTERM_CC_FAT	GO:0043233~organelle lumen	13	5.22088	0.54345	9.98E-01	CES3, MEAF6, BRD1, S	168	1820	12782	1.00000	1.00000	########
GOTERM_CC_FAT	GO:0031974~membrane-enclosed lumen	13	5.22088	0.53291	9.99E-01	CES3, MEAF6, BRD1, S	168	1856	12782	1.00000	1.00000	########
GOTERM_CC_FAT	GO:0070013~intracellular organelle lumen	11	4.41767	0.47044	1.00E+00	BRD1, MEAF6, CES3, C	168	1779	12782	1.00000	1.00000	########
GOTERM_CC_FAT	GO:0031981~nuclear lumen	8	3.21285	0.41977	1.00E+00	BRD1, MEAF6, CCR6, S	168	1450	12782	1.00000	1.00000	########

Annotation Cluster 85 Enrichment Score: 6.80208657524169E-4

Category	Term	Count	%	Fold Enrich	PValue	Genes	List Total	Pop Hits	Pop Total	Bonferroni	Benjamini	FDR
GOTERM_CC_FAT	GO:0044429~mitochondrial part	3	1.20482	0.38361	9.97E-01	TMEM173, ALDH4A1, A	168	595	12782	1.00000	1.00000	########
SP_PIR_KEYWORDS	mitochondrion	4	1.60643	0.37592	9.99E-01	SEPT4, TMEM173, ALD	246	832	19235	1.00000	1.00000	########
GOTERM_CC_FAT	GO:0005739~mitochondrion	5	2.00803	0.34997	1.00E+00	TMEM173, GCK, RIPK1	168	1087	12782	1.00000	1.00000	########
